# Chemically-Mediated Interactions Between Macroalgae, Their Fungal Endophytes, and Protistan Pathogens

**DOI:** 10.3389/fmicb.2018.03161

**Published:** 2018-12-21

**Authors:** Marine Vallet, Martina Strittmatter, Pedro Murúa, Sandrine Lacoste, Joëlle Dupont, Cedric Hubas, Gregory Genta-Jouve, Claire M. M. Gachon, Gwang Hoon Kim, Soizic Prado

**Affiliations:** ^1^Muséum National d'Histoire Naturelle, Unité Molécules de Communication et Adaptation des Micro-organismes, UMR 7245, CP 54, Paris, France; ^2^The Scottish Association for Marine Science, Scottish Marine Institute, Oban, United Kingdom; ^3^Institut Systématique Evolution Biodiversité (ISYEB), Muséum National d'Histoire Naturelle, CNRS, Sorbonne Université, EPHE, Paris, France; ^4^Unité Biologie des organismes et écosystèmes aquatiques (UMR BOREA), Muséum national d'Histoire Naturelle, Sorbonne Université, Université de Caen Normandie, Université des Antilles, CNRS, IRD; Station Marine de Concarneau, Concarneau, France; ^5^Université Paris Descartes, Laboratoire de Chimie-Toxicologie Analytique et Cellulaire (C-TAC), UMR CNRS 8638, COMETE, Paris, France; ^6^Department of Biology, Kongju National University, Kongju, South Korea

**Keywords:** fungal endophytes, macroalgae, protistan pathogens, secondary metabolites, metabolome, molecular interactions, pyrenocines

## Abstract

Filamentous fungi asymptomatically colonize the inner tissues of macroalgae, yet their ecological roles remain largely underexplored. Here, we tested if metabolites produced by fungal endophytes might protect their host against a phylogenetically broad spectrum of protistan pathogens. Accordingly, the cultivable fungal endophytes of four brown algal species were isolated and identified based on LSU and SSU sequencing. The fungal metabolomes were tested for their ability to reduce the infection by protistan pathogens in the algal model *Ectocarpus siliculosus*. The most active metabolomes effective against the oomycetes *Eurychasma dicksonii* and *Anisolpidium ectocarpii*, and the phytomixid *Maullinia ectocarpii* were further characterized chemically. Several pyrenocines isolated from *Phaeosphaeria* sp. AN596H efficiently inhibited the infection by all abovementioned pathogens. Strikingly, these compounds also inhibited the infection of *nori* (*Pyropia yezoensis*) against its two most devastating oomycete pathogens, *Olpidiopsis pyropiae*, and *Pythium porphyrae*. We thus demonstrate that fungal endophytes associated with brown algae produce bioactive metabolites which might confer protection against pathogen infection. These results highlight the potential of metabolites to finely-tune the outcome of molecular interactions between algae, their endophytes, and protistan pathogens. This also provide proof-of-concept toward the applicability of such metabolites in marine aquaculture to control otherwise untreatable diseases.

## Introduction

Macroalgae (seaweed) are important ecosystems engineers that contribute significantly to primary production in cold and temperate coastal seas and drive essential functions in nutrient cycling (Dayton, 1985). Macroalgae represent also a growing economic resource and their aquaculture has increased over the last decades, in particular for the Asian food market. In the last 25 years, the production of *Pyropia* (*nori*, formerly called *Porphyra*), the alga extensively used as sushi wrap in Asiatic cuisine, has more than tripled, mostly due to a rapid expansion in China and Korea. Accordingly, human consumption now represents 99% of the global algal market which keeps growing at an annual rate nearing 10% (Food and Agricultural Organization of the United Nations (FAO), 2014).

Like most eukaryotes, macroalgae are colonized by widely diverse microorganisms that interact with them throughout their life cycle (Singh and Reddy, 2015). Bacterial communities associated with seaweed have profound effects on their growth, defense, development, and nutrition (Egan et al., 2013). Epiphytic bacterial communities are essential to the morphological development of certain green algae (Wichard et al., 2015) and numerous studies demonstrated the contribution of bacteria to nutrient acquisition or defense by the production of vitamins (Wichard and Beemelmanns, 2018). These multiple interactions led to define macroalgae and their associated microbiota as a “superorganism,” called the holobiont (Egan et al., 2013).

Within this large microbial diversity, bacteria have been extensively studied, yet macroalgae also harbor a large diversity of fungi. The first report of an obligate mycophycobiosis between the Fucales *Ascophyllum nodosum, Pelvetia canaliculata*, and the fungal endosymbiont *Stigmidium ascophylli* (formerly *Mycosphaerella ascophylli*) dates back from more than a century (Cotton, 1907; Stanley, 1991), and it has been suggested that the symbiont may protect the algae host from desiccation, while obtaining nutrients in exchange (Garbary and Macdonald, 1995; Decker and Garbary, 2005). In the same vein, the fungal symbiont *Turgidosculum ulvae* colonizing the inner tissue of the green alga *Blidingia minima* can induce dark spots that are never consumed by the predatory gastropods of the host (Kohlmeyer and Volkmann-Kohlmeyer, 2003).

Many filamentous fungi can also asymptomatically colonize the algal inner tissues without causing any apparent damage or disease (Porras-Alfaro and Bayman, 2011; Debbab et al., 2012). These asymptomatic marine fungi remain mostly underexplored and only few reports can be found in the literature (Fries, 1979; Zuccaro et al., 2003, 2008; Harvey and Goff, 2010; Loque et al., 2010; Jones and Pang, 2012). However, thanks to recent DNA sequencing efforts, fungal diversity in marine substrata has been unraveled and now constitutes the second biggest known marine reservoir of fungi after sponges (Rateb and Ebel, 2011). This diversity encompasses mutualistic symbionts, opportunistic pathogens, parasites, and saprophytes (Zuccaro and Mitchell, 2005; Jones and Pang, 2012; Richards et al., 2012; Rédou et al., 2016). Recent studies related based on culture and molecular methods showed that ascomycetes are dominant endophytes of algae (Zuccaro et al., 2003, 2008; Flewelling et al., 2013a,b; Godinho et al., 2013).

In terrestrial plants, some well-known rhizosphere bacteria or root-colonizing fungi (i.e., *Fusarium* or *Trichoderma*) protect plants against phytopathogenic fungi or oomycetes (Haas and Defago, 2005; Terhonen et al., 2016). These mutualistic symbionts are often closely related to pathogens, and the beneficial nature of the interaction may depend on factors such as nutrition (Hiruma et al., 2016), age (Swett et al., 2017), or temperature (Tellenbach and Sieber, 2012). Similar examples are known in the phyllosphere; for example, cocoa trees inoculated with endophytes isolated from healthy leaves showed increased resistance to a *Phytophthora* pathogen (Arnold et al., 2003). The plant-endophyte coevolution hypothesis (Ji et al., 2009) suggests that endophytes might benefit plants by producing bioactive secondary metabolites. Indeed, endophytes isolated from *Pezicula* were shown to produce fungicidal metabolites toxic to the pathogens of their host (Arnold et al., 2003). Conversely, some endophytes may be latent or opportunistic pathogens when they exhibit virulence factors or produce toxic metabolites. Hence, the asymptomatic fungal colonization of plant organs would be the result of a balance between endophytic virulence and defense responses, preventing the development of disease (Schulz et al., 1999). In the phycosphere, endophytes are also able to produce bioactive antimicrobial metabolites (Zhang et al., 2009; Singh et al., 2015). It is thus plausible to assume that some secondary metabolites may mediate a mutualistic relationship, and may have a protective role toward other algae-associated microbiotes such as pathogens.

Macroalgae, comparable with plants in this context, are indeed subject to numerous biotic stressors (Gachon et al., 2010; Thomas et al., 2014) such as viral, bacterial, fungal, oomycete, chytrid pathogens, and algal colonization in the form of endo- or epiphytes. In the seaweed industry, the oomycetes *Olpidiopsis porphyrae* and *Pythium porphyrae* are the most destructive pathogens of laver (Kim et al., 2014). As the industry quickly develops and intensifies, pathogen outbreaks are becoming a growing cause of concern. A recent *Olpidiopsis* outbreak in Korea was estimated to have reduced sale volumes by a quarter, notwithstanding the cost of disease management (Kim et al., 2014; Gachon et al., 2017). Crop protection measures are at best partially effective, and novel treatments are needed.

In brown algae, the obligate endobiotic oomycetes *Eurychasma dicksonii* and *Anisolpidium ectocarpii* are frequently found in the field (Sparrow, 1903) and able to infect more than 45 and 28 brown algal species in laboratory cultures, respectively (Gachon et al., 2010, 2017). They are amongst very few eukaryotic pathogens of algae that can be cultivated under laboratory conditions, including in the filamentous model brown seaweed *Ectocarpus siliculosus*.

The parasite *Maullinia ectocarpii* belongs to the Phytomyxea, a phylogenetically distant group of Rhizaria, can infect four different algae orders, including the Ectocarpales and Laminariales (Maier et al., 2000). This parasite is closely related to *Maullinia braseltonii*, an economically significant pathogen of bull kelp (Murúa et al., 2017). Therefore, these model pathogens constitute a powerful tool to investigate the responses of brown algae to infection, as their broad host range suggests that successful infection involves the overcoming core, conserved immune defenses. These pathogens can be co-cultivated with their host and used as a pathosystem to perform functional bioassays. Compared to the economically-important kelp sporophytes, *Ectocarpus* is an excellent model organism, the microbiota of which can be easily manipulated (Müller et al., 2008; Tapia et al., 2016). As *Ectocarpus* is mostly composed of thin filaments, the commonly found endosymbionts are bacteria. The availability of its genome sequence as well as the possible powerful applications of genomics and genetics allow molecular and functional investigations (Peters et al., 2004).

Here, we hypothesized that secondary metabolites produced by endophytic fungi associated to brown algae may protect their host against several protistan pathogens of seaweeds, i.e., *E. dicksonii, A. ectocarpii*, and *M. ectocarpii*. We thus characterized the fungal endophytic strains isolated from *A. nodosum, P. canaliculata, Laminaria digitata*, and *Saccharina latissima* and demonstrated that some of them are able to protect the algal model *E. siliculosus* against pathogenic infection through the production of fungal metabolites belonging to the pyrenocine series (Figure S1). The pathosystem Eurychasma/Ectocarpus is one of the very few *in vivo* bioassay available to test the effect of substances against protistan pathogens of macroalgae. This broad-spectrum protection effect of the pyrenocines expanded to the most economically important oomycetes infecting the red seaweed Pyropia, *Olpidiopsis pyropiae*, and *P. porphyrae*. Notably, the pyrenocines are active against all tested pathogens but are also algicidal for the host *E. siliculosus* at higher concentration. These findings suggest for the first time that brown algae-derived endophytes may shape the infection outcome of algal pathogens by chemically protecting their host through the production of small chemicals cues.

## Materials and Methods

Laminariales *L. digitata* (LD) and *S. latissima* (SL) and two Fucales *A. nodosum* (AN) and *Pelvetia caniculata* (PC) according to the sampling data (Table S1). Three individuals of each species were collected during maxima low tide and processed within 2 h of collection. Algae organs of 5 cm^2^ (receptacles, thalli, stipes, fronds, and holdfasts) were excised and surface-sterilized by sequential immersion in Ethanol 70% (30 s), in NaOCl 0.1% (30 s), and washed three times (30 s) in sterilized sea water (Kjer et al., 2010; Kientz et al., 2011). Algal segments were plated in multiples onto 4,200 plates representing 10 solid media (3% purified agar, pH 7.5, 80% sterilized sea water) with the internal tissues in contact with the medium (about 200 segments per organ, 5,600 per individual, 16,800 per species-host, 1,400 per medium type) and solidified with 20 g.L^−1^ of purified agar (except for PDA). These media included Corn Flour Agar (Cornflour, La Vie Claire^©^, 10 g.L^−1^), Biomalt Agar 1 (Biomalt, Villa Natura^©^, 1 g.L^−1^), Biomalt Agar 2 (Biomalt, Villa Natura^©^, 20 g.L^−1^), Potato Dextrose Agar (Potato Dextrose Agar, Conda^©^, 20 g.L^−1^), Yeast Extract Agar 1 (glucose 1 g.L^−1^, soymeal peptone 0.5 g.L^−1^, yeast extract 0.08 g.L^−1^), Yeast Extract Agar 2 (glucose 0.1 g.L^−1^, soymeal peptone 0.05 g.L^−1^, yeast extract 0.01 g.L^−1^), Malt Extract Agar (malt extract 20 g.L^−1^, glucose 20 g.L^−1^, peptone 1 g.L^−1^), Tubaki Agar (glucose 30 g.L^−1^, yeast extract 1 g.L^−1^, peptone 1 g.L^−1^, K_2_HPO_4_ 1 g.L^−1^, MgSO_4_ 0.5 g.L^−1^, FeSO_4_ 0.01 g.L^−1^), Provasoli Agar (Na_2_ β-glycero PO_4_.5H_2_O 50 g.L^−1^, NaNO_3_ 35 g.L^−1^, Iron-EDTA (Fe(NH_4_)_2_(SO_4_)_2_.6H_2_O 0.7 g.L^−1^, Na_2_EDTA 0.6 g.L^−1^), Vitamin B_12_ 0.01 g.L^−1^, Thiamine 0.5 g.L^−1^, Biotin 0.005 g.L^−1^, PII trace metals (Na_2_EDTA 1 g.L^−1^, H_3_BO_3_ 1.12 g.L^−1^, MnSO_4_H_2_O 0.12 g.L^−1^, ZnSO_4_.7H_2_O 0.022 g.L^−1^, CoSO_4_.7H_2_O 0.005 g.L^−1^), *Stigmidium* Fries Agar (KNO_3_ 0.72 g.L^−1^, K_2_HPO_4_ 1.2 g.L^−1^, vitamin solution 1 mL (thiamin 100 mg.mL^−1^, biotin 25 mg.mL^−1^), MgSO_4_.7H_2_O 0.7 g.L^−1^, NaCl 10 g.L^−1^, glucose 20 g.L^−1^, traces element solution 1 mL (CaCl_2_ 100 mg.mL^−1^, ZnSO_4_.7H_2_O 4.43 mg.mL^−1^, MnSO_4_.4H_2_O 4.03 mg.mL^−1^, Ferric citrate C_6_H_5_FeO_7_ 4 mg.mL^−1^). Plates were incubated at 18°C under ambient light and checked daily for endophytic growth up to 3 months. The percentages of infection per algal organ and per algal species were calculated by applying the following formulae: number of isolates recovered / total number of algal pieces deposited on agar medium.

### Sequencing and Molecular Identification of Algae-Associated Fungal Endophytes

**Figure 1:**
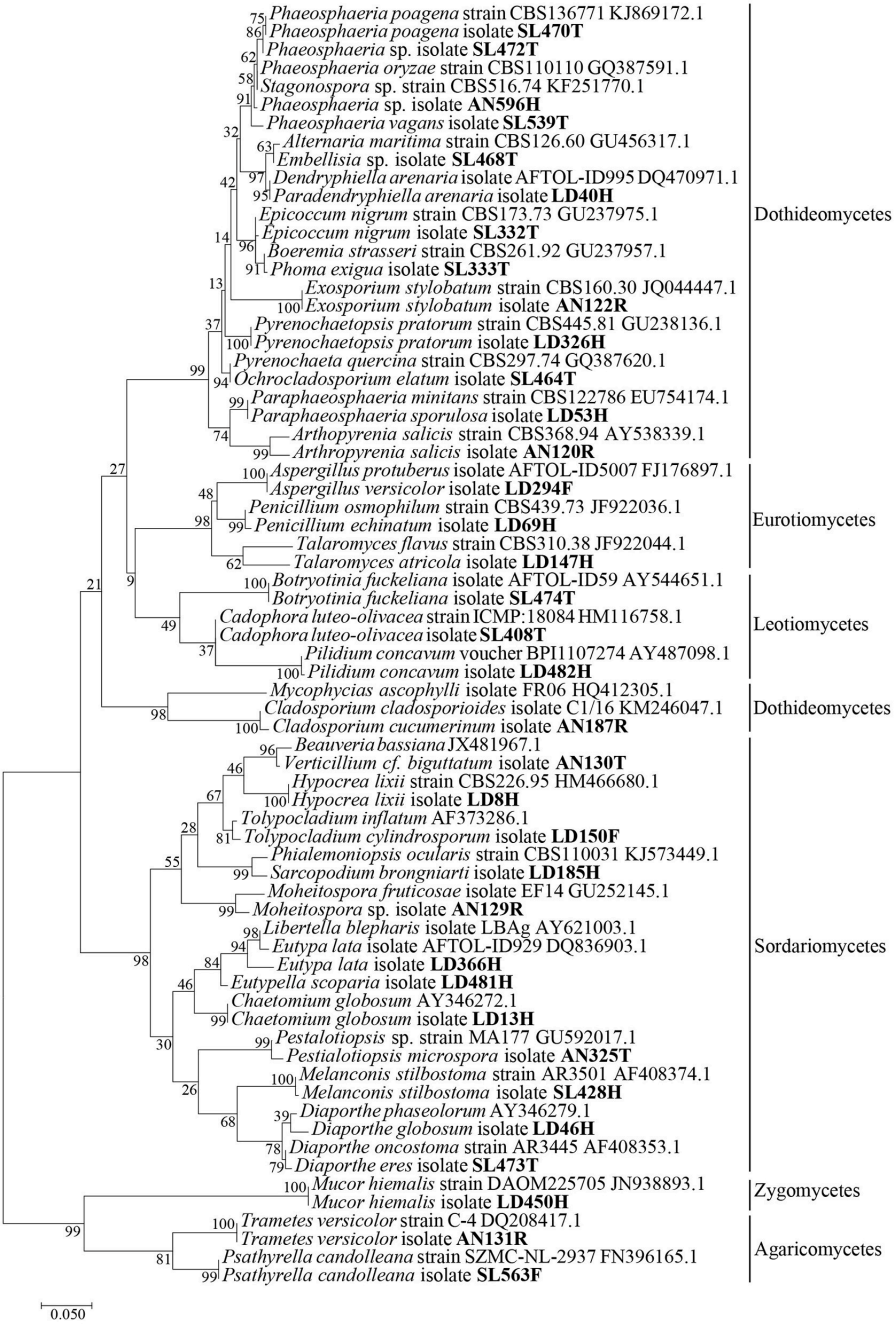
Phylogenetic affinities based on LSU sequences of fungal endophytes isolates from the four brown algae sampled from French and Scottish sites. Isolate code are indicated in bold and accession numbers from reference sequences are marked in gray. Scale bar indicates 10% estimated sequence divergence.

Fungal genomic DNA was extracted from fresh mycelium grown on different solid media. Extractions were performed using dneasy Plant Mini Kit (QIAGEN, Ltd. Crawley, UK) following the manufacturer's instructions. Different sets of primers were used to amplify different marker genes: LROR/LR6 primers (Vilgalys and Hester, 1990; Vilgalys and Lin Sun, 1994) were used to amplify a 600 base pair portion at the 5′ end of the nuclear ribosomal DNA large subunit, ITS4/ITS5 (White et al., 1990) were used to amplify the internal transcribed spacer region of the Ribosomal RNA operon, Bt2a/Bt2b (Glass and Donaldson, 1995) for a part of the 5' end of the beta tubulin coding gene when the ITS sequences were not informative enough to discriminate species of *Aspergillus, Penicillium*, and *Talaromyces*. PCR amplifications were performed according to previous studies (Lopez-Villavicencio et al., 2010; Langenfeld et al., 2013). PCR products were purified and sequenced using the dideoxy termination (Sanger) reaction by Eurofins MWG Operon® in Germany. Sequences were assembled with CodonCode Aligner v. 3.7.1. (Codon Code Corporation), checked by visual inspection of the chromatograms and edited if necessary. Molecular operational taxonomic units (MOTU) were defined using an arbitrary but commonly used threshold of 3% intra-specific variability (Altschul et al., 1990; Nilsson et al., 2008; Tamura et al., 2013). MOTU were identified using the Blastn alignment tool (megablast algorithm optimized for highly similar sequences and excluding uncultured/environmental sample sequences) at http://blast.ncbi.nlm.nih.gov/Blast.cgi (Altschul et al., 1990). Best hits were carefully examined and sequences from taxonomic reference strains (AFTOL, CBS, DAOM, NRRL) were chosen preferably to attribute species names (≥97% of sequence similarities). Sequences were aligned using the ClustalW tool available on MEGA version 6.0 software (Tamura et al., 2013). The phylogenetic tree was build using the same software, under a maximum likelihood framework. We used model test (Posada and Crandall, 2001) to choose the best nucleotide substitution model, namely GTR+I+G. Support for the branches was determined from bootstrap analysis of 1,000 resampled datasets (Figure 1). Sequences were deposited in GenBank under the accession numbers MH397587-MH397621 (28S) and MH397623-MH397663 (ITS) (Alignments S1–S3).

### Microorganisms, Fermentation, Extraction

From the 99 isolates obtained, 70 were able to yield enough biomass for subsequent experiments. To build the extracts library, these strains were inoculated in a 12 cm2 Petri dish containing 60 mL (agar 1.5%) of their respective isolation medium. The inoculation was made with spore suspension (100 μL, 10^4^ spores.mL^−1^) or from crushed mycelial suspension (1 cm2 in 2 mL of sterile ASW) for non-sporulating fungi. After incubation at 18°C for 27 days under a 12 h light:12 h dark photoperiod, whole fungal culture (mycelium and 60 mL agar) were cut in 1 cm2 pieces and extracted by ethyl acetate (3 × 200 mL) for 3 × 1 h under sonication. The organic phases were filtrated, dried over anhydrous MgSO_4_ and concentrated under vacuum to yield crude extracts.

### Pyrenocines Isolation and Identification

The fungi *Phaeosphaeria* sp. AN596H was grown in 5 L Erlenmeyer flasks (5 × 600 mL of TUA inoculated with 5 mL fungal suspension in sterile sterile deionized water) at 18°C for 27 days under a 12 h light:12 h dark photoperiod. Extraction was performed with ethyl acetate (analytical grade, 3 × 5 L) under a mechanical agitation, organic phases were dried by anhydrous MgSO_4_, filtered, and concentrated under low vacuum to yield 2.3 g of a brown solid extract. The extract was solubilized in methanol and subjected to Sephadex® LH-20 column chromatography (Sigma-Aldrich, Germany) with a 100% methanol elution. The active fraction (82 mg) was further purified by preparative HPLC (Agilent PrepHT, column Eclipse XDB-C18 21.2 × 150 mm, 5 μm) under a flow rate of 10 mL.min^−1^ of analytical grade solvents buffer A: 95% water 5% Acetonitrile, B: 95% Acetonitrile 5% water. This lead to the isolation of pyrenocines A (R_t_: 13 min, 7 mg), B (R_t_: 8 min, 5.5 mg), E (R_t_: 14 min, 1 mg), and pyrenochaetic acid C (R_t_: 23 min, 1.8 mg) along with the novel pyrenocine S (R_t_ 19 min, 2 mg). The infrared spectra were recorded on a Shimadzu FTIR-8400S spectrophotometer. Mass spectra were acquired on an API Q-STAR PULSAR mass spectrometer (Applied Biosystem, Bruker). NMR experiments were performed with Bruker advance III 400 and 600 MHz spectrometers (Wissenbourg, France). Chemical shifts are expressed in δ (ppm) and were referred to the residual non-deuterated solvent signal.

### Disease Resistance Assays Using Algae-Pathogen Pathosystems

Female gametophytes of *Macrocystis pyrifera* CCAP1323/1 infected with either *E. dicksonii* CCAP 4018/1 or CCAP 4018/3 were maintained in half-strength Provasoli medium at 15°C under daylight-type fluorescent lamps (10 μmol.m^−2^.s^−1^, 12 h light:12 h dark photoperiod). Batches of fast-growing cultures *E. siliculosus* CCAP 1310/4 were produced in 200 mL flasks. *E. siliculosus* was harvested in 70 μm pore-size nylon mesh (Cell strainer, Falcon) and equivalent amounts were co-incubated in six-well polystyrene plates containing infected *M. pyrifera* CCAP 1323/1 in 7 mL of half-strength Provasoli liquid medium for 16–20 days. The infections with *A. ectocarpi* CCAP 4001/1 and *Maullinia ectocarpi* CCAP1538/1 were performed with the same hosts during 3 and 10 days of co-incubation, respectively. The library of fungal extracts (1 μg.mL^−1^ in dimethyl sulfoxide DMSO) was evaluated for their antipathogenic effect on the different pathogens. In all experiments, three controls were performed and consisted in incubating *Ectocarpus* alone (control uninfected), with parasites (control infected), and with addition of 1% DMSO (control infected + DMSO 1%). The activity of purified compounds (0.1 μg.mL^−1^ in 1% DMSO) was assessed with the same procedure. After samples anonymization, filamentous *E. siliculosus* were delicately collected with extra thin needles (size 10–15, Hemline®) and briskly transferred in 100 μl of sterile seawater between slide and coverslip. Samples were maintained in a moist chamber and analyzed in the following hour. The infection of *E. siliculosus* filaments by protistan parasitoids was assessed under a microscope Axiovert2plus Zeiss (DIC, Plan Apochromat. 20 × 0.75) and pictures taken with camera Axocam HRc were analyzed with AxioVisio software (version 4.7). A scoring scale was defined by a (0) score if no infected cell was observed and (1, 2, 3) scores, respectively, for 1–10, 11–100, or superior to 100 infected cells were observed. The *Ectocarpus* samples were then frozen at −80°C in RNAlater stabilization reagent (QIAGEN). An identical protocol was used to evaluate the toxic activity of the pyrenocines on the algae *E. siliculosus* alone at 1 μg.mL^−1^ in 1% DMSO.

### Evaluation Against Oomycete Pathogen *Olpidiopsis Pyropia*

Gametophytic blades of *Pyropia yezoensis* infected with *O. pyropiae* were maintained in half-strength Provasoli medium at 15°C under daylight-type fluorescent lamps (10 μmol.m^−2^·s^−1^, 12 h photoperiod). The set up for the bioassay was exactly as those for evaluation against pathogens of *E. siliculosus* in six-well plates. The pyrenocines and pyrenochaetic acid C were added to the culture at the final concentration of 1 μg.ml^−1^ (in DMSO 1%). Immediately after addition, infected area were observed for 10 min with an Olympus BX53 microscope (Olympus, Tokyo, Japan) and videos were recorded by a mounted Olympus DP72 camera.

### Pathogen Quantification With qPCR

The relative abundance of the SSU rRNA for *E. siliculosus* and for *E. dicksonii* was quantified by qPCR using the validated primers pair (CG64/CG65) and (CG60/CG61), respectively (Amagata et al., 1998), as described in Gachon et al. (2009). PCR reactions were carried out in triplicate on a Quantica thermocycler (Techne-Barloworld) or a LightCycler® Roche (version 96SW1.1). The PCR mix for 1 sample contained 10 μL 2x Mesagreen qPCR MasterMix Plus for SYBR® Assay, 1 μL of each primer (final concentration 300 nM), 8 μL DNA template for 20 μL volume total. After a 10 min denaturation step at 95°C, samples were run for 45 cycles of 15 s at 95°C and 1 min at 60°C, followed by a dissociation curve. The disease scores were calculated by subtracting the threshold value obtained in the DNA amplification curve from the pathogen with that of the algae amplification curve. The disease scores were compared statistically by a one-way ANOVA with *post-hoc* Tukey HSD test.

## Results

### Diversity and Phylogenetic Relationships of Filamentous Fungi Associated With Brown Macroalgae

The diversity of endophytes was evaluated as previously described for endophytic isolation associated with macro-algae (Zuccaro et al., 2008), using a stringent disinfection protocol to avoid surface-associated microbial contamination. Concisely, 99 clonal isolates in total were purified from the 2,100 algae organs segments (Table 1), of which 30 were isolated from *L. digitata* (10 from Scotland and 20 from France), 17 from *S. latissima* in Oban, 49 strains from *A. nodosum* (5 from Scotland and 44 from France), and 3 from *P. canaliculata*. The frequency of colonies retrieved was low, with 99 positive segments over 2,100 incubated (4.7%), and was irregular, ranging from 0 to 29% per algae organ and 1–14.6% per algae species (Table 1).

**Table 1:** Distribution of isolates and MOTU (Molecular Operational Taxonomic Units) in the algal organs according to the sampling sites.

**Host**	**Sampling site**	**Nb of isolates/Nb of MOTU**	**Nb of isolates/Nb of MOTU**	**Nb of isolates/Nb of MOTU**	**Nb of isolates/Nb of MOTU**	**Total Nb of isolates**	**Nb of singletons**	**Nb of different MOTUs**	**Percentage of infection per algae**
		**Thallus**	**Frond**	**Receptacle**	**Holdfast**			
SL	Oban	15/14	1/1		1/1	17	12	16	5.6
PC	Oban	1/1		0/0	2/1	3	0	1	1
LD	Oban	0/0	0/0		10/9	10	5	9	3.3
LD	Roscoff	0/0	2/2		18/14	20	10	16	6.6
AN	Oban	4/4		0/0	1/1	5	2	5	1.6
AN	Roscoff	15/2		29/7	0/0	44	6	8	14.6

*A total of 100 tissue segments per algal organ were analyzed for each of the 6 host-site. Codes are Nb, number; SL, Saccharina latissima; PC, Pelvetia canaliculata; LD, Laminaria digitata; AN, Ascophyllum nodosum*.

**Figure 2:**
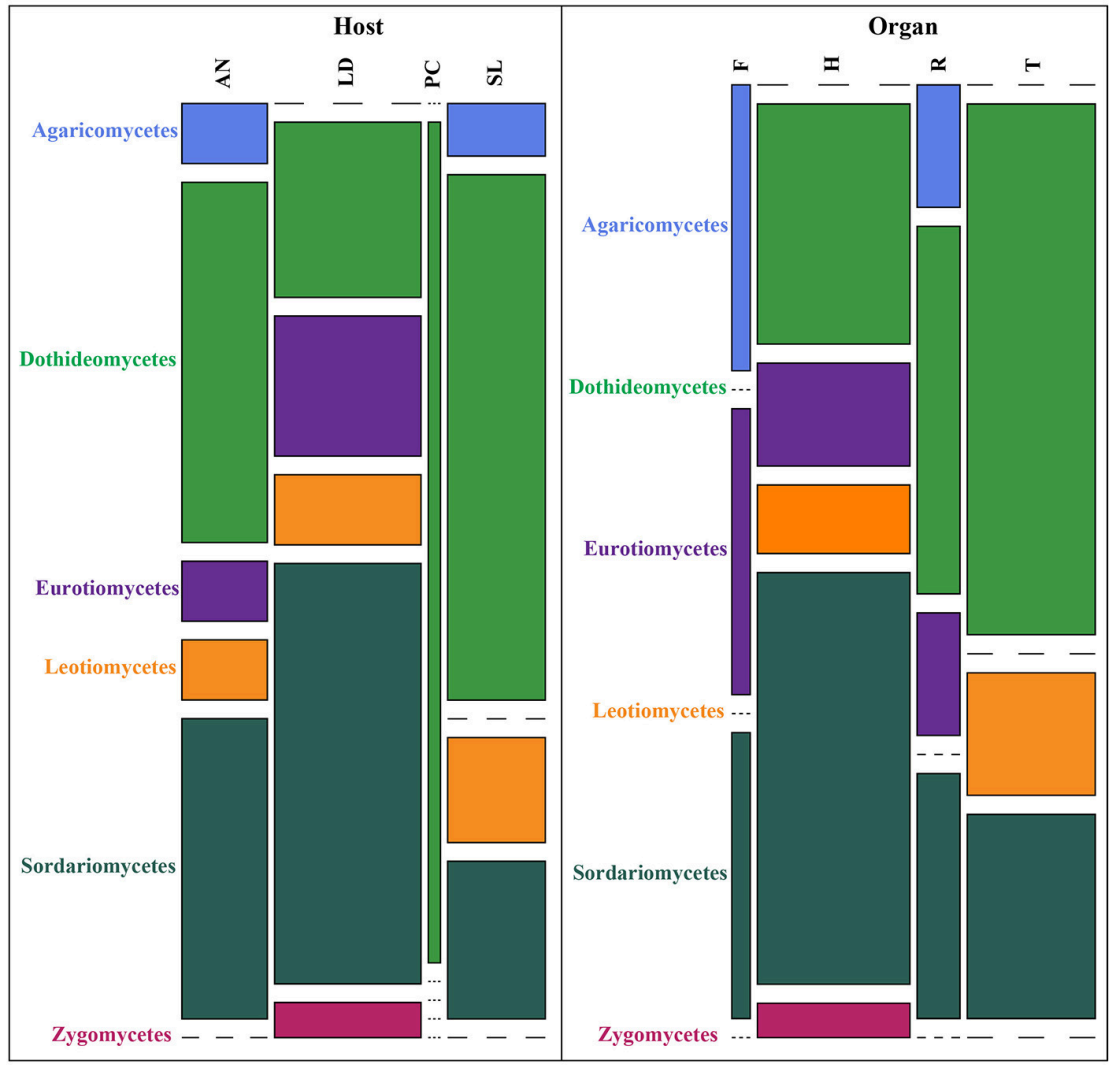
Taxonomic assemblages of the fungal classes determined according to the different host-algae species and algal organs. Each class is displayed by a different color. Height of the bars represents the % of each fungal class according to the host-algae species or the algal organs. Width of the bars represents the total number of OTUs according to the host-algae species or the algal organs. Codes for host species and algal organs are SL, *Saccharina latissima*; PC, *Pelvetia canaliculata*; LD, *Laminaria digitata*; AN, *Ascophyllum nodosum*; F, Frond; H, Holdfast; R, Receptacle; T, Thallus.

The receptacles were infected only in French *A. nodosum* (29% of the segments), while no fungi was isolated from the receptacles of Scottish Fucales. The most infected algae organs were the thalli (35 isolates) and holdfasts (32 isolates) but isolation success was irregular according to the algae species. Each algal segment yielded only one fungal strain at most. Among the 99 fungal isolates, 45 MOTU were identified, of which 35 were singletons (Table S2). An overview of phylogenetic relationships of the algicolous fungal isolates with referent fungal strains (Table S3) is summarized in a tree reconstructed based on LSU rDNA sequences alignment (Figure 1). Eurotiomycetes were further investigated by targeting the Tubuline marker (SI. Alignment 2) while all the fungal isolates identity was confirmed by sequencing the ITS region (SI. Alignment 3). The taxonomic assemblages and prevalence of the fungal classes recovered from the different host species and per algal organs were summarized in a mosaic-plot representation (Figure 2). Two MOTU were found to be predominant in this study, i.e., an unknown marine ascomycete related to *Moheitospora* sp. and the obligate marine fungus *Paradendryphiella arenaria*, representing, respectively, 37.4 and 8.1% of the isolates. The MOTU were phylogenetically diverse; most of them belong to Ascomycota (93.3%) and very few to Basidiomycota (4.4%) and Zygomycota (2.2%). The fungal community of the Ascomycota was dominated by Dothideomycetes (37.8%), with Pleosporales as the most abundant order, followed by Sordariomycetes (35.6%), Eurotiomycetes (11.1%), and Leotiomycetes (6.7%) (Figure 2). In the sequence analysis, the taxonomic diversity was high but MOTU were mostly present as singletons (16 MOTU/17 isolates from *S. latissima* in Oban, 9 MOTU/10 isolates from *L. digitata* in Oban, 16 MOTU/20 isolates from *L. digitata* in Roscoff). The exception was in *A. nodosum* harvested in Roscoff where a lower diversity was observed with 8 MOTU identified among 44 isolates. However, most of the isolates (37 isolates: 14 in the thalli and 23 in the receptacles) belonged to same MOTU (Table S2). According to the sequence analysis, the fungi is related with 98–99% of identity to several Sordariomycetes marine fungi, *Moheitospora fruticosae, Juncigena adarca*, and *M. chaetosa* previously described by Kohlmeyer (Abdel-Wahab et al., 2010) or Jones (Singh and Reddy, 2015). Only one MOTU, the obligate marine fungus *P. arenaria*, was present in all algae species.

### Screen of Fungal Metabolome Inhibiting Infection by Oomycete and Phytomixid Pathogens

**Figure 3:**
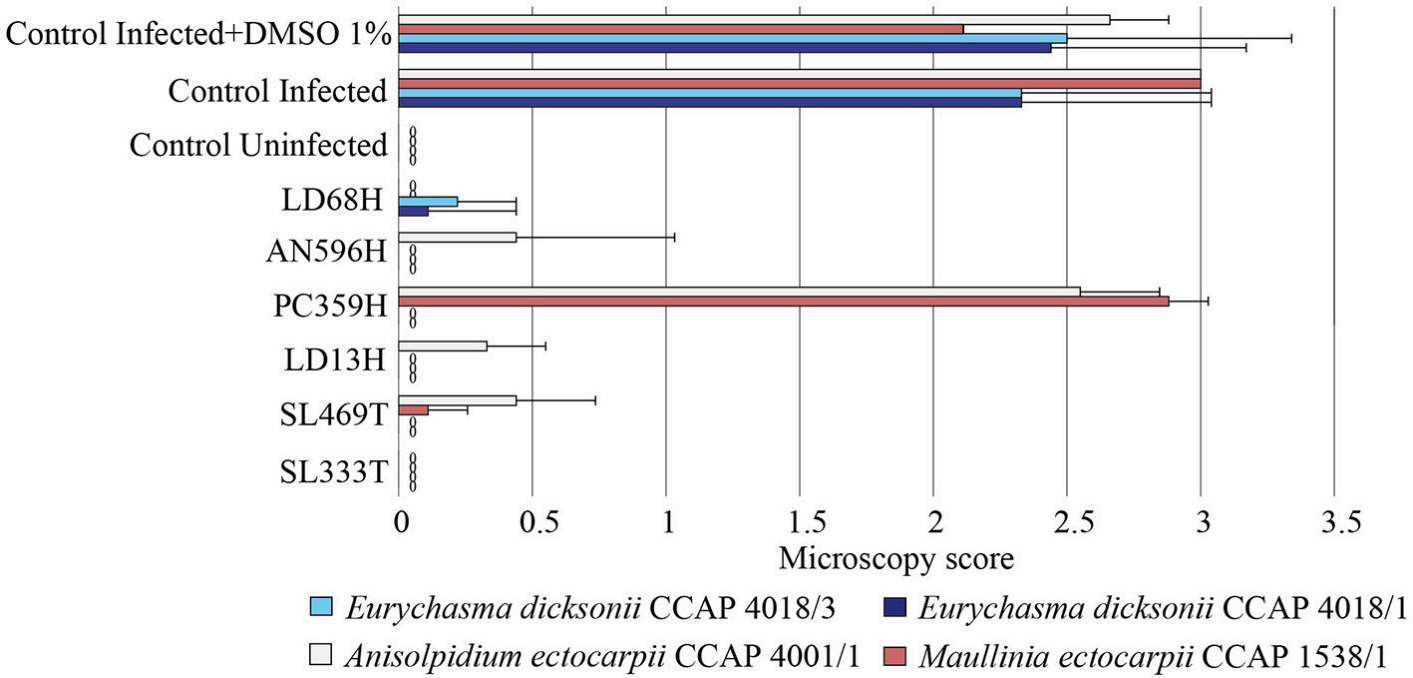
Antipathogenic activities of the fungal extracts LD68H *(Penicillium janczewskii)*, AN596H (*Phaeosphaeria* sp.), PC359H *(Paradendryphiella arenaria)*, LD13H (*Chaetomium globosum)*, SL469T (*Chaetomium globosum)*, and SL333T (*Phoma exigua)* assessed on the infection of *Ectocarpus siliculosus* by *Eurychasma dicksonii* (CCAP 4018/1, CCAP 4018/3), *Anisolpidium ectocarpii* (CCAP 4001/1), and *Maullinia ectocarpii* (CCAP 1538/1). Controls consisted of algae alone (control uninfected), algae with parasite treatment (control infected), algae with parasite treatment, and 1% DMSO (control infected + DMSO 1%). Mean microscopy score values ± SE are displayed for biological triplicates.

To assess the functional role of the isolated fungi against pathogens of seaweed, their extracts were tested against the pathosystem *Ectocarpus*/parasites. The brown seaweed *E. siliculosus* was thus infected by the oomycetes *E. dicksonii, A. ectocarpii*, or the phytomixid *M. ectocarpii* in presence of the fungal crude extracts. DMSO 1% was used as control and did not display any effect on the parasites' infection. This initial screening allowed the identification of five fungal extracts (*Penicillium janczewskii* LD68H, *Phaeosphaeria* sp. AN596H, *Chaetomium globosum* LD13H, *C. globosum* SL469 T, and *Phoma exigua* SL333T) which inhibit the infection by all the pathogens at 10 μg.mL^−1^ with a broad-range spectrum (Figure 3). However, the *P. arenaria* PC359H extract displayed a specific activity only against the two strains of *E. dicksonii*.

### Molecular Quantification of *Eurychasma Dicksonii* Infection

**Figure 4:**
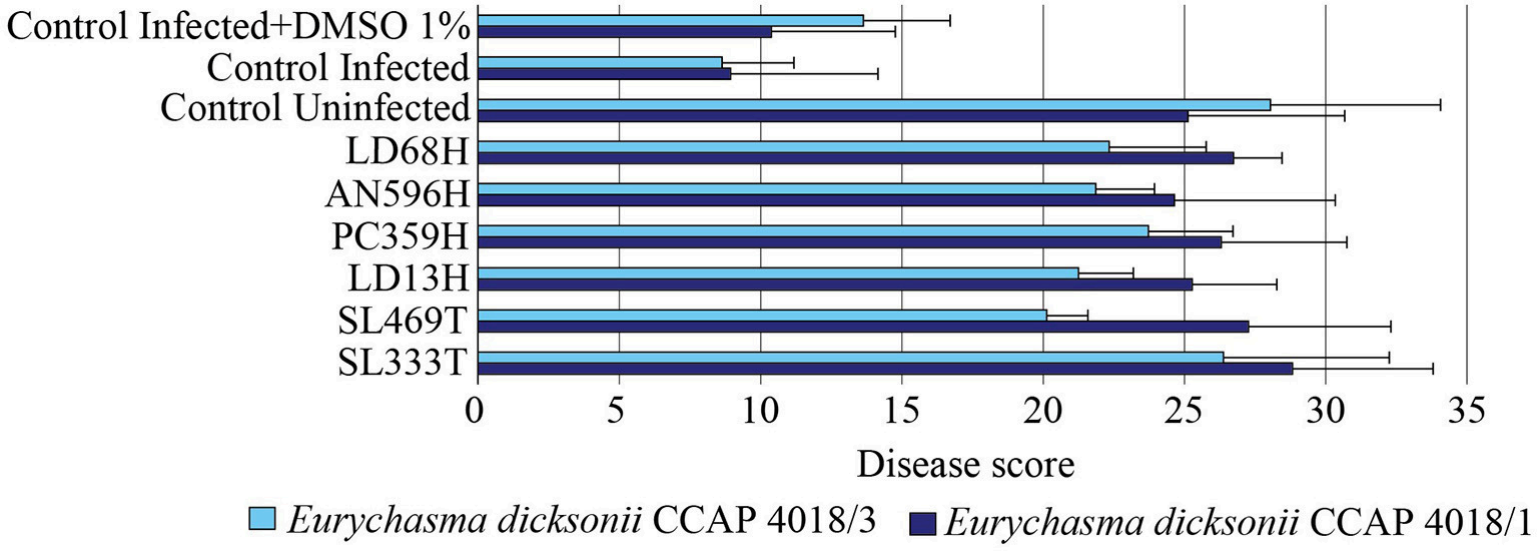
Antipathogenic activities of the fungal extracts LD68H *(Penicillium janczewskii)*, AN596H (*Phaeosphaeria* sp.), PC359H *(Paradendryphiella arenaria)*, LD13H (*Chaetomium globosum)*, SL469T (*Chaetomium globosum)*, and SL333T (*Phoma exigua)* assessed by qPCR quantification of the infection of *Ectocarpus siliculosus* by *Eurychasma dicksonii* (CCAP 4018/1, CCAP 4018/3). Controls consisted of algae alone (control uninfected), algae with parasite treatment (control infected), algae with parasite treatment and 1% DMSO (control infected + DMSO 1%). Mean disease score ± SE are displayed for biological triplicates.

In order to corroborate the microscopy results, the infection was also quantified by RT-qPCR for the experiments with *E. dicksonii* strains CCAP4018/1 and CCAP4018/3. The relative abundance of DNA from both host and pathogen was calculated (Gachon et al., 2009). A low disease score (<20) corresponds to a high infection while a high score (>20) indicate a lowly-infected to uninfected algae. A linear regression analysis showed a positive correlation (*R*^2^ = 0.715) between the results obtained with the microscopy and qPCR evaluation methods (Figure S2). The results showed that the six most potent extracts unveiled by the microscopy assessment displayed high disease scores (Figure 4), confirmed further by one-way ANOVA with *post-hoc* Tukey HSD test (*F* = 10.97, *p* < 0.001). Hence, the inhibition against tested algal pathogens by crude extracts obtained from *Phaeosphaeria* sp. AN596H, *P. janczewskii* LD68H, *P. arenaria* PC359H, *C. globosum* SL469T, *C. globosum* LD13H, and *P. exigua* SL333T were confirmed by two independent quantification methods.

### Secondary Metabolites Produced by Algicolous Fungi *Phaeosphaeria* sp. AN596H

**Figure 5:**
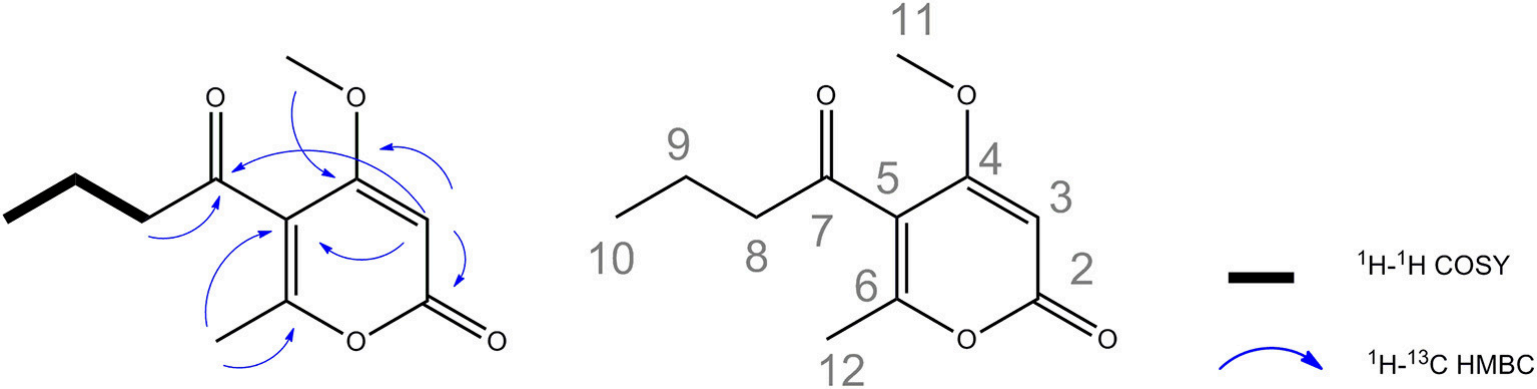
Key HMBC and COSY correlations of the new pyrenocine S.

With respect to the potent antipathogenic activities, the identity of active compounds was investigated by a bioassay-guided fractionation method for the six fungal extracts. We succeed to recover enough quantity of the active fraction for further characterization only for the fungi *Phaeosphaeria* sp. AN596H. The fungal extract was subjected to successive chromatographic separation and yielded the pure compounds **(1**-**5)** (Figure S3). These compounds were structurally characterized by spectral comparison with the literature and belong to the pyrenocines family (Sato et al., 1979, 1981a,b; Amagata et al., 1998). Pyrenocines A and B were the predominant compounds isolated, among the other minor pyrenocines E, C and pyrenochaetic acid C. The assignment of the absolute configuration of pyrenocines B and E relied on the comparison of the theoretically calculated experimental values of optical rotations from the present study (Figure S3). In the course of this work, a new minor compound was also characterized and named pyrenocine S (Figure S3). This compound with the molecular formula C_11_H_15_O_4_ was deduced from the molecular peak at *m*/*z* 211.0942 [M+H]^+^ (calc. 211.0970) and *m*/*z* 233.0792 [M+Na]^+^ (calc. 233.0790) in high-resolution mass spectrometry. The ^1^H NMR spectrum (Table S4, Figure S4) of **1** showed signals for three methyl groups, including a triplet at δ_H_ 0.95 (3H, J = 7.4 Hz, H-10), a singlet at δ_H_ 2.20 (3H, H-12), and a methoxy at δ_H_ 3.91 (3H, H-11), two methylene at δ_H_ 1.65 (2H, J = 7.4 Hz, H-9), and 2.73 (2H, J = 7.2 Hz, H-8) and an ethylenic proton resonating at δ_H_ 5.64 (1H, H-3). ^13^C NMR spectrum of **1** (Table S4, Figure S5) indicated the presence of five sp^3^ carbons: three methyls (δ_C_ 13.9, 18.2, and 57.4) and two methylenes (δ_C_ 18.4 and 47.6). ^13^C NMR spectrum of **1** also displayed one sp^2^ methine (δ_C_ 88.5, C-3) and five sp^2^ carbons (including two carbonyls at δ_C_ 165.3 and 202.6, two oxygenated at δ_C_ 163.4, and 170.7 and a quaternary at δ_C_ 117.4). ^1^H-^1^H COSY spectrum was indicative of a spin system between the both methylenes H-8, H-9 and the methyl CH_3_-10 (Figure 5). The HMBC correlations (Figure 5 and Figure S6) between the methine H-3 and C-4 (δ_C_ 170.7)/C-5 (δ_C_ 117.4)/C-2 (δ_C_ 165.3) as well as the correlations between the methyl CH_3_-12 (δ_H_ 2.20) and C-6 (δ_C_ 163.4)/C-5 (δ_C_ 117.4) suggested the presence of a methyl-pyran-2-one moiety in the structure. Moreover, HMBC correlation between methoxy (δ_H_ 3.91) and C-4 (δ_C_ 170.7) allowed to connect the methoxy group on C-4. Finally, HMBC correlations of methylenes CH_2_-8 and CH_2_-9 with the ketone C-7 at δ_C_ 202.6 ppm as well as the HMBC correlations between H-3 and C-7 confirm the attachment of the aliphatic chain on C-5 of the cycle. All these data revealed the structure is a new pyrenocine for which the name pyrenocine S was proposed. All identified compounds were thus further tested against the pathosystem *Ectocarpus*/parasites.

### Antiparasitic Activity of Pyrenocines and Pyrenochaetic Acid C

**Figure 6:**
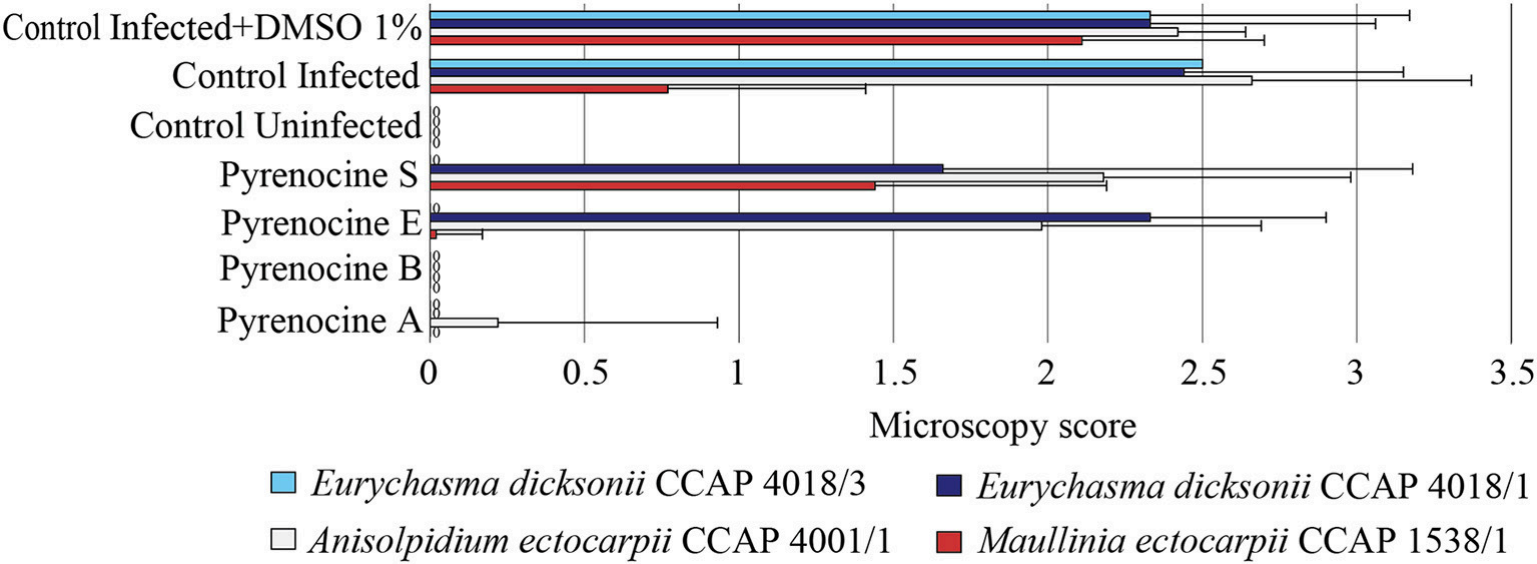
Antipathogenic activities of the isolated assessed on the infection of *Ectocarpus siliculosus* by the pathogens *Eurychasma dicksonii* (CCAP4018/1, CCAP4018/3), *Anisolpidium ectocarpii* (CCAP 4001/1), and *Maullinia ectocarpii* (CCAP 1538/1). Controls consisted of algae alone (control uninfected), algae with parasite treatment (control infected), algae with parasite treatment and 1% DMSO (control infected + DMSO 1%). Mean microscopy score values ± SE are displayed for biological triplicates.

To address the activity of the pyrenocines against protistan pathogens of brown algae, we screened compounds **1-4** for their potential inhibitory effect on the infection of *E. siliculosus* by *E. dicksonii, A. ectocarpii*, and *M. ectocarpii* (Figure 6). The pyrenocines A and B displayed a strong antipathogenic effect at 0.1 μg.ml^−1^ (0.4 μM) with a broad range spectrum in our assessment, while the other minor compounds did not have any effect except pyrenocines E and S active only against the strain *E. dicksonii* CCAP 418/3 (Figure 6). These results were confirmed by the qPCR evaluation for pyrenocine A (one-way ANOVA, *F* = 11.91, *p* < 0.001). It is worth noting that in the course of our investigation, the pyrenocines were also tested at 10-fold higher concentration (4 μM) and induced the death of algal host cells (Figure S7).

### Protective Effect of Pyrenocines and Pyrenochaceatic Acid Against Oomycete Pathogens of Laver

The pyrenocines and pyrenochaetic acid C were tested against the oomycetes *O. pyropiae* and *P. porphyrae* in the algae *P. yezoensis*. Pyrenocine A yielded a total inhibition of the infection of the host alga at 1 μg.ml^−1^ (48 μM) while the pyrenochaetic acid C stopped the growth of *P. porphyrae* mycelium and induced its degeneration in 3 days. For instance, the collapse of zoosporangia of *O. pyropia* could be clearly observed with microscopy only few second after treatment with pyrenochaetic acid C (SI. Movie 1). Altogether, none of the added compounds induced any detectable changes in morphology, growth, and reproduction of the seaweed host *P. yezoensis*. These results clearly demonstrated the broad range effect of pyrenocines against protistan pathogens of seaweed.

## Discussion

This study highlighted a high phylogenetic diversity (45 MOTU) among the cultivable fungal endophytic community associated with the inner tissues of brown algae *L. digitata, S. latissima, A. nodosum*, and *P. caniculata*. The fungal species belonged to the Ascomycota, especially the Sordariomycetes and the Dothideomycetes (Figure 1). The proportion of taxa recovered within the main orders and classes were relatively similar to the ones described for marine fungi associated to plants or algae, in comparison with the public SSU rRNA reference sequences (Panzer et al., 2015). The prevalence of cultivable endophytes was very low (4.7% overall) in comparison to the one commonly observed for terrestrial plants, as for example, fungi were successfully isolated from 99% of the *Cephalotaxus harringtonia* leaves tested (Langenfeld et al., 2013). The present findings are also consistent with the few existing surveys of cultivable endophytes associated with algae (Zuccaro et al., 2008; Flewelling et al., 2013a), but perhaps also due to our stringent surface disinfection protocol. However, only a few *Penicillium* and *Aspergillus* strains were isolated in this study in contrast to the fungal community associated with *S. latissima* harvested in the Atlantic Coast of Canada (Flewelling et al., 2013b).

Additionally, the taxonomic diversity and abundance of isolates differed between the algal organs tested, suggesting a potential tissue, and host preference. This pattern of fungal colonization may be explained by the differences in chemical composition and defense in algal species and organs (Megan et al., 2001; Cosse et al., 2009; Thomas et al., 2014). Notably, none of the fungi isolated from the brown algae *A. nodosum* was identified as *Stigmidium ascophylli*, despite its being a well-documented Dothideomycetes mycophycobiont (Fries, 1979; Garbary and Gautam, 1989; Garbary et al., 1991; Stanley, 1991; Garbary and London, 1995; Garbary and Macdonald, 1995). However, a predominant fungus forming pink colonies similar to the cultivable one described by Fries (Fries, 1979) has been recovered from this algae and was identified as a Sordariomycete related to *M. fruticosae, J. adarca*, and *Marinokulati chaetosa*, that were previously described by Kohlmeyer (Abdel-Wahab et al., 2010) or Jones (Singh and Reddy, 2015). Our results also suggest that a second endophytic fungus may occur alongside *Mycophycias ascophylli* as a predominant colonizer of *A. nodosum*. A thorough year-long sampling across the coasts would certainly help in determining the natural prevalence of these fungal endophytes in the macroalgal community.

Aside from the cosmopolitan *P. arenaria*, which occurred in all four species investigated, few marine fungi *sensu stricto* or unknown species have been isolated (Michaelis et al., 1987; Cruz dela, 2006). Instead, most of the recovered strains are closely related to terrestrial phytopathogens (37%), endophytes (21%), or a miscellaneous group of lignivore, soil-borne, and air-borne saprophytes (28%) according to previous in the literature. Several genera, i.e., *Acremonium, Coniothyrium, Botryotinia, Phaeospharia*, and *Cordiceps* have not been previously isolated from marine algal hosts (Flewelling et al., 2013a; Godinho et al., 2013,b). In particular, we retrieved sequences matching the phytopathogens *P. exigua* and *Botryotinia fuckeliana*. One hypothesis is therefore that these strains might be opportunistic pathogens, perhaps able to colonize an otherwise compromised alga. Nonetheless, known plant pathogens were found to live as endophytes in the liverwort *Marchantia polymorpha* and were able to confer growth benefits in a laboratory bioassay (Nelson et al., 2018). Hence, further research is required to ascertain the type of the interaction between these recovered fungi and brown algae, which could either be mutualist or pathogen. It should be borne in mind that these interactions may also depend upon the host genetic background, as shown recently for bacterial endosymbiont of insects (Cass et al., 2016).

Among the six fungal extracts that prevented or strongly inhibited infection by all three protistan pathogens of brown algae tested, the one obtained from *Phaeosphaeria* sp. AN596H lead to the successful chemical characterization of the active fraction. This fungal genus was found predominant in endophytes recovered from cold-adapted marine macrophytes (Zhang and Yao, 2015). Furthermore, a strain of *Phaeosphaeria spartinae* was isolated from the red alga *Ceramium* sp. and produced unusual polyketides and steroids (Elsebai et al., 2008; Elsebai et al., 2013). However, the ecological role of the fungus and its metabolites remain unknown.

Here, the fungal strain *Phaeosphaeria* sp AN596H was recovered from *A. nodosum* and five polyketides from the pyrenocine family were characterized. The pyrenocines A and B inhibit the infection of *E. siliculosus* by the protistan pathogens at the concentration of 0.1 μg.ml^−1^. Pyrenocine A was first reported from the phytopathogen *Pyrenochaeta terrestris* (Sato et al., 1981a,b) and recently from the sponge-derived endophyte *Penicillium paxilli* (Toledo et al., 2014). The cytotoxic and antimicrobial properties of the pyrenocines has been reported in terrestrial ecosystems as, for example, pyrenocine A inhibits phytopathogens, and gram-positive bacteria at micromolar concentrations (Sparace et al., 1987; Rukachaisirikul et al., 2007; Toledo et al., 2014). This compound also displayed algicidal activity on *E. siliculosus* at 1 μg.ml^−1^, but not on the red algae *P. yezoensis* at a similar concentration. Therefore, we hypothesize that at low concentration, fungal pyrenocines may confer protection to the alga against protistan pathogens, while being toxic at higher concentrations to some seaweed. However, the physiological concentrations at which pyrenocines were produced within the host *A. nodosum* are unknown and will have to be further investigated. The minor compounds pyrenocine E, pyrenochaetic acid C, and the new natural product pyrenocine S were also identified in the active extract, but did not displayed a broad range activity against the different protistan parasites. Pyrenocines E and S were found active against the strain *E. dicksonii* CCAP 418/3 giving evidence for strain-specific effect. Difference of activity for pyrenocines compounds could be also explained by their structural difference on the lateral chain suggesting structure-activity relationships.

Altogether, these results constitute the first example of a possible chemical mediation involved in a molecular interaction within the algal microbiota. We suggest that the endophytic continuum paradigm defined *in planta* might also possibly occur in brown algae (Schulz et al., 1999; Schulz and Boyle, 2005). Pathosystem bioassays with the alga model *E. siliculosus* were performed to identify fungal extracts and later the pyrenocines which are bioactive against phylogenetically unrelated pathogens. Importantly, these compounds also inhibit the infection of the most widely cultivated cultivar of laver in Korea by its two most important pathogens, the oomycetes *O. pyropiae*, and *P. porphyrae*.

## Conclusions

These results provide for the first time evidence of fungal endophytes associated with brown macroalgae may protect their host *in vivo* through the production of small molecules. These data demonstrated that an active chemical defense produced by the algal microbiota may drive the infection success of pathogenic microbiotes in the phycosphere. Further studies using a broader range of algae species should be performed to evaluate the conservation of this chemical defense amidst hosts. Hence, these findings provide a proof-of-concept to pursue the detailed chemical characterization of the other bioactive extracts identified, with the view to identify novel molecules with application in seaweed crop protection.

## Author Contributions

MV, PM, and MS carried out the experiments. MV, JD, and SL analyzed the data of molecular taxonomy. GG-J performed optical rotation calculations. MV, SP, and CG wrote the manuscript with the help of CH, JD, and GHK. All authors conceived this study and approved the manuscript.

### Conflict of Interest Statement

The authors declare that the research was conducted in the absence of any commercial or financial relationships that could be construed as a potential conflict of interest.
