# Supplementary material for: Chemically-Mediated Interactions Between Macroalgae, Their Fungal Endophytes, and Protistan Pathogens
Source: Front Microbiol. 2018 Dec 21;9:3161. doi: 10.3389/fmicb.2018.03161 (PMC6309705; doi:10.3389/fmicb.2018.03161)
Supplement: Supplementary file 1 [file Data_Sheet_1.PDF]

Supplementary Material

Chemically-mediated interactions between macroalgae, their fungal endophytes, and protistan pathogens

Marine Vallet[1], Martina Strittmatter[2], Pedro Murúa[2], Sandrine Lacoste[3], Joelle Dupont[3], Cedric Hubas[4], Gregory Genta-Jouve[5], Gwang-Hoon Kim[6], Claire M.M. Gachon[2], Soizic Prado[1*]

*** Correspondence:**

Soizic Prado: sprado@mnhn.fr

Figure S1. Research scheme and workflow.

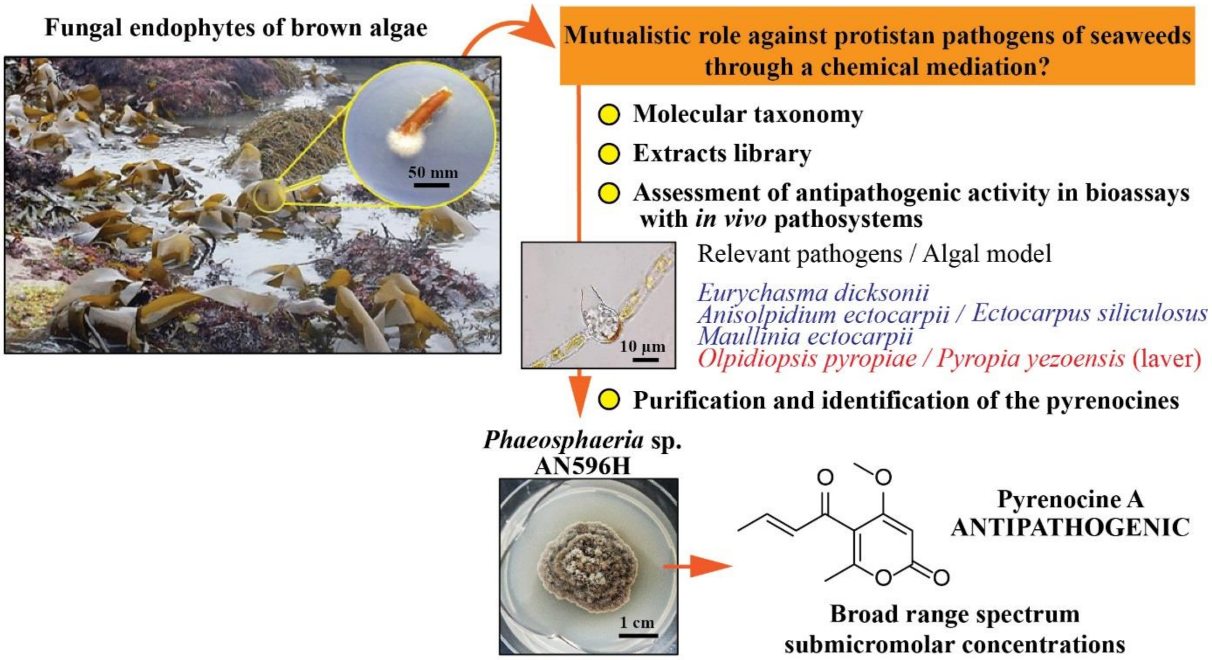

Table S1. Sampling design.

Sampling site	Roscoff, France	Oban, Scotland, UK
Date of collection	January 2013	July 2013
GPS coordinates N/W	48° 42.66' / 3° 54.02'	56° 19' 3.1 / 5° 35' 1.2
Algae collected (3 individuals per site/per species)	*A. nodosum*, *L. digitata*	*A. nodosum*, *L. digitata*, *S. latissima*, *P. canaliculata*
Organs collected from Laminariales (100 per individual)	Frond, Thallus, Holdfast, Stipe	
Organs collected from Fucales (100 per individual)	Receptacle, Thallus, Holdfast	

SI. Table S2. Identification of endophytes by the top BLAST match of their ITS rDNA or Tubulin sequences (*Aspergillus* sp., *Penicillium* sp.) and distribution of isolates in the different hosts and organs according to the sampling sites. N°: number. LD: *Laminaria saccharina* (previously *Laminaria digitata*), SL: *Saccharina latissima*, AN: *Ascophyllum nodosum*, PC: *Pelvetia canaliculata*. Ro: Roscoff, Ob: Oban. H: Holdfast, S: stipe, R: receptacle, T: thallus, F: frond.

**Fungal species**	**Classification Class/order**	**N° of isolates/host /site/organ***	**Percentage of identity**	**GenBank N°/Isolate N°**
Chaetomium globosum	Sordariomycetes Sordariales	1/LD/Ro/H<br>1/LD/Ob/H	100	KC109754/LD13H<br>KC109754/SL469T
Diaporthe phaseolorum	Sordariomycetes Diaporthales	1/LD/Ro/H	99	EU272538.1/LD46H
Diaporthe eres	Sordariomycetes Diaporthales	1/SL/Ob/T	99	KC343073.1/SL473T
Phomopsis mali	Sordariomycetes Diaporthales	1/LD/Ro/H	99	AB665315.1/LD14H
Melanconis stilbostoma	Sordariomycetes Diaporthales	1/SL/Ob/H	99	AY577814.1/SL428H
Tolypocladium cylindrosporum	Sordariomycetes Hypocreales	1/LD/Ro/F	99	AB208110.1/LD150F
Hypocrea lixii	Sordariomycetes Hypocreales	1/LD/Ro/H	100	GU566194.1/LD8H
Cordiceps brongniartii	Sordariomycetes Hypocreales	2/LD/Ro/H<br>1/SL/Ob/T	99	AB258367.1/LD144H

Sarcopodium oculorum	Sordariomycetes Hypocreales	1/LD/Ro/H	99	EF063140/LD185H
Eutypa consobrina	Sordariomycetes Xylariales	1/LD/Ro/H	99	EU552126.1/LD54H
Eutypa lata	Sordariomycetes Xylariales	1/LD/Ob/H	99	HQ288221.1/LD366H
Eutypella scoparia	Sordariomycetes Xylariales	1/LD/Ob/H	98	EU436688.1/LD481H
Pestialotiopsis microspora	Sordariomycetes Xylariales	1/AN/Ob/T	98	AF377292.1/AN325T
Marinokulati chaetosa *Moheitospora fruticulosae* *Juncigena adarca*	Sordariomycetes	14/AN/R/T<br>23/AN/R/R	99/98<br>98	KJ866832/AN129R<br>GU252145.1/AN129R<br>EF027726/AN129R<br>(LSU rDNA)
Verticillium cf. *biguttatum*	Sordariomycetes Plectosphaerellaceae	1/AN/Ro/T	99	EF641856.1/AN130T
Parasphaeosphaeria sporulosa	Dothideomycetes Pleosporales	2/LD/Ro/H	99	JX496074.1/LD53H
Paraphaeosphaeria neglecta	Dothideomycetes Pleosporales	1/LD/Ob/H<br>1/LD/Ro/H	99	JX496067.1/LD449H
Pyrenochaetopsis pratorum	Dothideomycetes Pleopsorales	2/LD/Ob/H	99	NR_111623.1/LD326H
Phoma exigua	Dothideomycetes Pleopsorales	1/SL/Ob/T	99	EU167567.1/SL333T
Microsphaeropsis olivacea	Dothideomycetes Pleopsorales	2/LD/Ro/H<br>1/AN/Ob/T	99	JX681101.1/LD50H
Paradendryphiella arenaria	Dothideomycetes Pleopsorales	2/LD/Ro/H<br>1/AN/Ob/T<br>2/SL/Ob/T<br>2/PC/Ob/H<br>1/PC/Ob/T	99	HQ649989/LD40H
Brunneiperidium gracilentum	Sordariomycetes Xylariales	1/AN/Ro/R	100%	KP297401.1/AN44R
Exosporium stylobatum	Dothideomycetes Pleosporales	1/AN/Ro/R	99	JQ044428.1/AN122R
Roussoella sp.	Dothideomycetes Pleosporales	1/AN/Ro/R	98	KX228312/AN120R
Ochrocladosporium elatum	Dothideomycetes Pleosporales	1/SL/Ob/T	95	GU248334.1/SL464T
Epiccocum nigrum	Dothideomycetes Pleosporales	1/SL/Ob/T	99	GU566259.1/SL332T
Phaeosphaeria poagena	Dothideomycetes Pleosporales	1/SL/Ob/T	99	KJ869114.1/SL470T
Phaeosphaeria sp.	Dothideomycetes Pleosporales	1/AN/Ob/H	98	KF251191.1/AN596H

Phaeosphaeria vagans	Dothideomycetes Pleosporales	1/SL/Ob/T	99	KF251193.1/SL539T
Phaeosphaeria sp.	Dothideomycetes Pleosporales	1/SL/Ob/T	99	KF251268.1/SL472T
Cladosporium cucumerinum	Dothideomycetes Capnodiales	1/AN/Ro/R<br>1/SL/Ob/T	99	KF156302.1/AN187R
Cladosporium cladosporioides	Dothideomycetes Capnodiales	1/SL/Ob/T	100	HM148035.1/SL405T
Aspergillus versicolor	Eurotiomycetes Eurotiales	1/LD/R/F	99	AJ937749.1/LD294F
Penicillium polonicum	Eurotiomycetes Eurotiales	1/LD/R/H	99	AY674306.1/LD330H
Penicillium janczewskii	Eurotiomycetes Eurotiales	1/LD/R/H	98	KJ834460/LD68H
Penicillium brevicompactum	Eurotiomycetes Eurotiales	1/AN/R/R	99	EU587353.1/AN312R
Talaromyces atricola	Eurotiomycetes Eurotiales	1/LD/R/H	97	KF196849.1/LD147H
Pilidium concavum	Leotiomycetes Helotiales	1/LD/Ob/H	99	AY487097.1/LD482H
Botryotinia fuckeliana	Leotiomycetes Helotiales	1/LD/Ob/H<br>1/SL/Ob/T<br>1/AN/Ob/T	100	DQ491491.1/SL474T
Cadophora luteo-olivacea	Leotiomycetes Helotiales	1/SL/Ob/T	99	JQ796751.1/SL408T
Ascomycetes	Ascomycetes	1/LD/Ob/H	97	AF261659/LD534H
Alternaria sp.	Dothideomycetes Pleosporales	1/SL/Ob/T	99	JN859370.1/SL468T
Psathyrella candolleana	Agaricomycetes Agaricales	1/SL/Ob/F	99	DQ494689.1/SL563F
Trametes versicolor	Agaricomycetes Polyporales	1/AN/Ro/R	99	JN164919.1/AN131R
Mucor hiemalis	Zygomycetes Mucorales	1/LD/Ob/H	100	JN942878.1/LD450H

SI. Table S3. Fungal species references selected for phylogenetic comparison with fungal isolates from the brown macroalgae.

**Fungal species identity**	**Accession number**	**Reference**
Phaeosphaeria poagena strain CBS 136771	KJ869172.1	*Persoonia* **32**, 184-306 (2014)
Phaeosphaeria oryzae strain CBS 110110	GQ387591.1	*Mycologia* **102** (5), 1066-81 (2010)
Stagonospora sp.	KF251770.1	*Studies in Mycology* **75** (1), 307-390 (2013)

strain CBS 516.74		
Alternaria maritima strain CBS 126.60	GU456317.1	*Studies in Mycology* **64**, 85-102S5 (2009)
Dendryphiella arenaria isolate AFTOL-ID995	DQ470971.1	*Mycologia* **98** (6), 1018-1028 (2006)
Epicoccum nigrum strain CBS 173.73	GU237975.1	*Studies in Mycology* **65**, 1-60 (2010)
Boeremia strasseri strain CBS 261.92	GU237957.1	*Studies in Mycology* **65**, 1-60 (2010)
Exosporium stylobatum strain CBS 160.30	JQ044447.1	*Persoonia* **27**, 130-162 (2011)
Pyrenochaetopsis pratorum strain CBS 445.81	GU238136.1	*Studies in Mycology* **65**, 1-60 (2010)
Pyrenochaeta quercina strain CBS 297.74	GQ387620.1	*Mycologia* **102** (5), 1066-81 (2010)
Paraphaeosphaeria minitans strain CBS 122786	EU754174.1	*Mycological Research* **113** (4), 508-519 (2009)
Roussoella solani strain CPC 26331	KX228312	Fungal Planet description sheets: 400-468
Penicillium osmophilum strain CBS 439.73	JF922036.1	*Fungal Biology* **115** (11), 1100-1111 (2011)
Talaromyces flavus strain CBS 310.38	JF922044.1	*Fungal Biology* **115** (11), 1100-1111 (2011)
Botryotinia fuckeliana isolate AFTOL-ID59	AY544651.1	*American Journal of Botany* **91** (10), 1446-80 (2004)
Cadophora luteo-olivacea strain ICMP:18084	HM116758.1	*Mycotaxon* **113**, 385-396 (2010)
Pilidium concavum voucher BPI1107274	AY487098.1	*Mycological Progress* **3** (4), 275-290 (2004)
Mycophycias ascophylli isolate FR06	HQ412305.1	*Botanica Marina* **54** (3), 325-334 (2011)
Tolypocladium inflatum	AF373286.1	*Mycological progress* **1** (1), 3-17 (2012)
Phialemoniopsis ocularis strain CBS 110031	KJ573449.1	*Journal of Clinical Microbiology* **52** (9), 3280-3289 (2014)
Moheitospora fruticosae isolate EF14	GU252145.1	*Mycological Progress* **9** (4), 537-558 (2010)
Libertella blepharis isolate LBAg	AY621003.1	*Journal of Phytopathology* **153** (4), 193-202 (2005)
Eutypa lata isolate AFTOL-ID929	DQ836903.1	*Mycologia* **98** (6), 1076-1087 (2006)
Chaetomium globosum	AY346272.1	*Mycologia* **96** (2), 368-387 (2004)
Pestalotiopsis sp. strain MA177	GU592017.1	Buatong, Thesis, Prince of Songkla University (2010) http://kb.psu.ac.th/psukb/handle/2010/8793
Melanconis stilbostoma strain AR3501	AF408374.1	*Mycologia* **94** (6), 1017-1031 (2002)

Diaporthe phaseolorum	AY346279.1	*Mycologia* **96** (2), 368-387 (2004)
Diaporthe oncostoma strain AR3445	AF408353.1	*Mycologia* **94** (6), 1017-1031 (2002)
Mucor hiemalis strain DAOM225705	JN938893.1	*PNAS* **109** (16), 6241-6246 (2012)
Trametes versicolor strain C-4	DQ208417.1	*Journal of Microbiology* **44** (1), 29-34 (2006)
Psathyrella candolleana strain SZMC-NL-2937	FN396165.1	*Systematic Biology* **60** (3), 303-317 (2011)

Figure S2. Linear regression between disease scores and microscopy scores for the quantification of the infection by *E. dicksonii* (CCAP4018/1, CCAP4018/3).

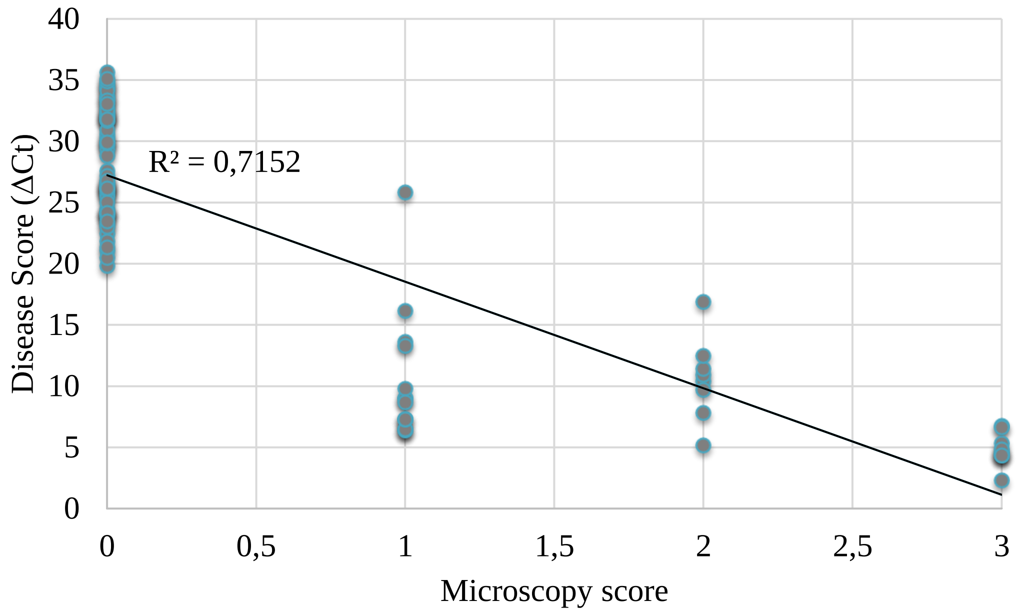

Figure S3. Structures of pyrenocine S **(1)** pyrenocine A **(2)** pyrenocine B **(3)** pyrenocine E **(4)** pyrenochaetic acid C (**5**).

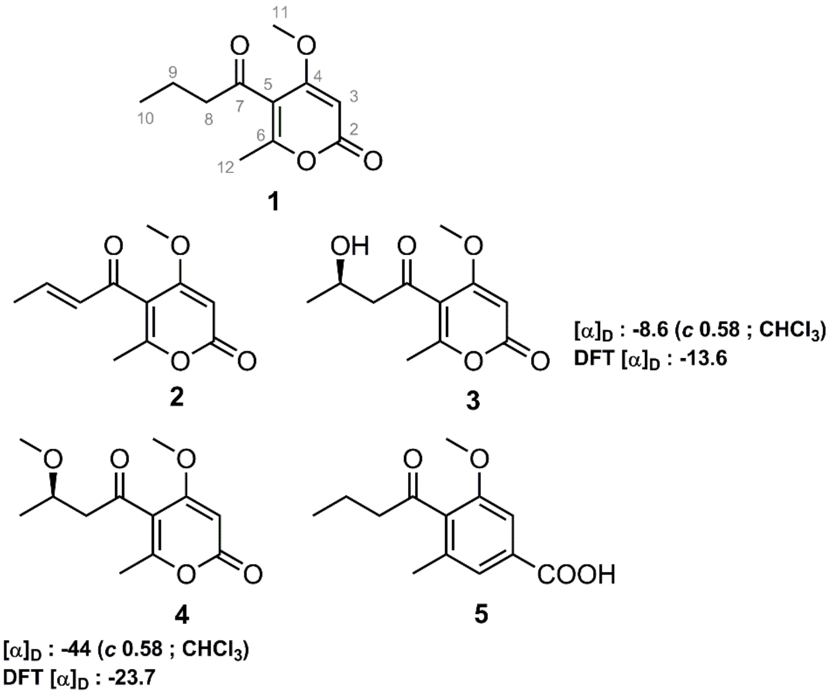

The assignment of the absolute configuration of **3** and **4** relied on the comparison of the theoretically calculated experimental values of optical rotations from the present study. All calculations have been performed using Gaussian 16W. All Optical rotation calculations have been performed on the most stable confomers (DeltaE < 1kcal/mol). 22 and 17 conformers were obtained for **3** and **4** respectively. The geometry optimization and optical rotation have been performed using the B3LYP method at the 6-31g(d) level. After Boltzmann weighting, the optical rotation were calculated for both compounds, -13.6 and -23.7 for **3** and **4** respectively.

Table S4. ^{1}H and ^{13}C NMR data of compound **1** (^{1}H and ^{13}C 400 MHz, 298K).

Position	δ_C	δ_H	Mult.	J (Hz)
2	165.3			
3	88.5	5.64	s	
4	170.7			
5	117.4			
6	163.4			
7	202.6			
8	47.6	2.73	t	7.2
9	18.4	1.65	m	-
10	13.9	0.95	t	7.4
11	57.4	3.91	s	
12	18.2	2.20	s	

Figure S4. ^{1}H NMR spectrum of Pyrenocine S in CD_3OD (400 MHz, 298K).

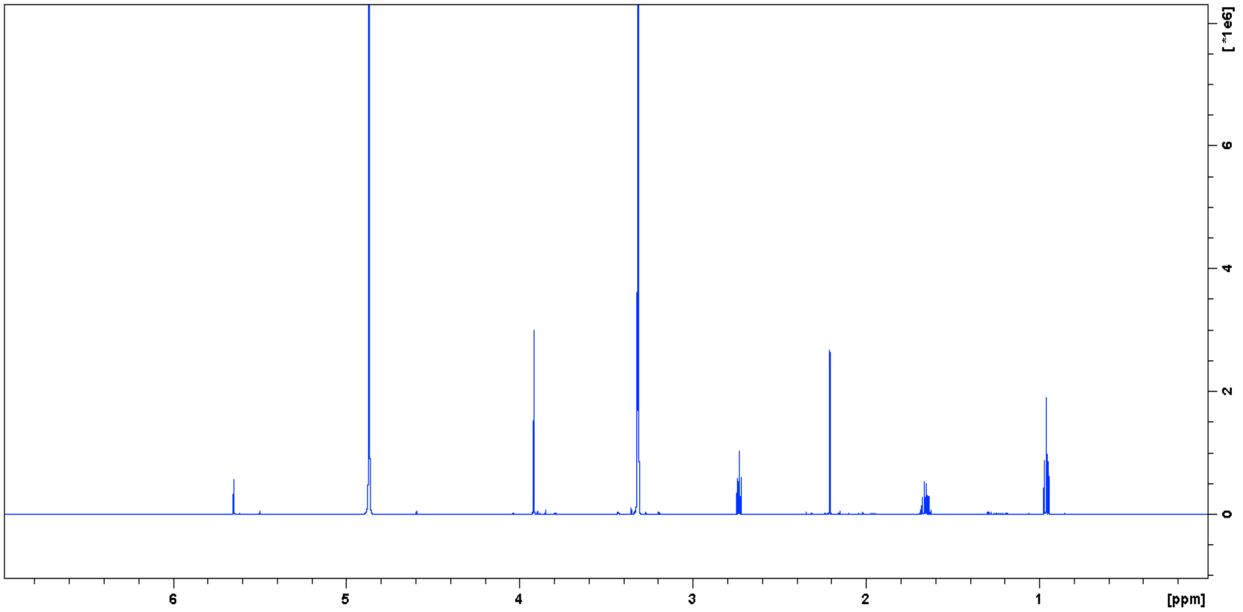

Figure S5. ^{13}C NMR spectrum of Pyrenocine S in CD_3OD (400 MHz, 298K).

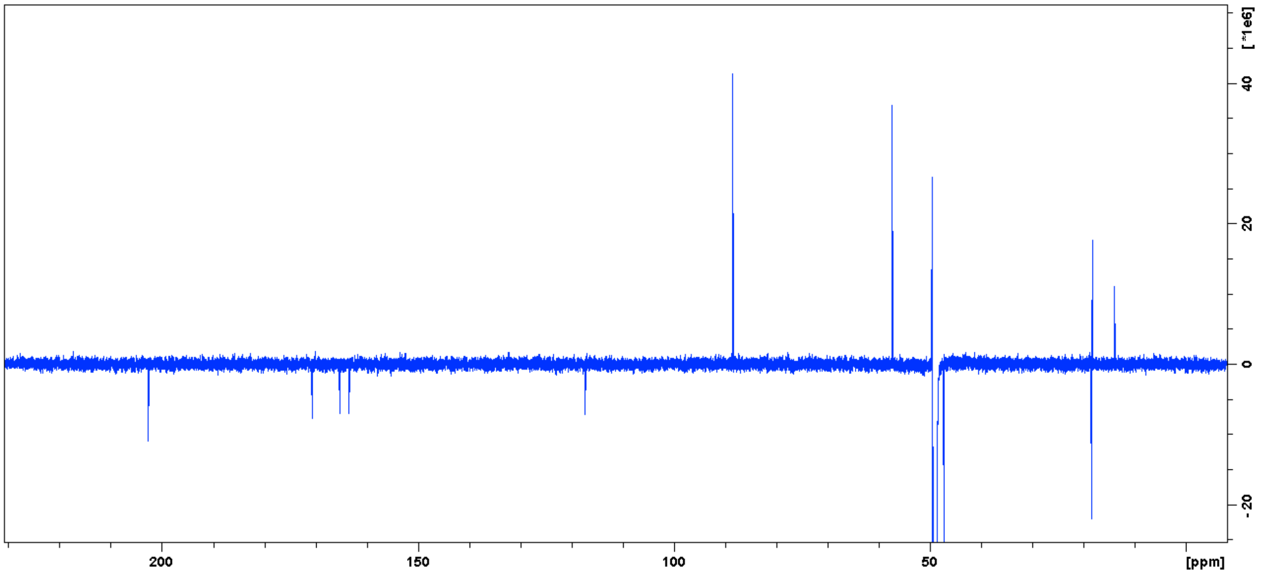

Figure S6. HMBC spectrum of Pyrenocine S in CD_3OD (600 MHz, 298K).

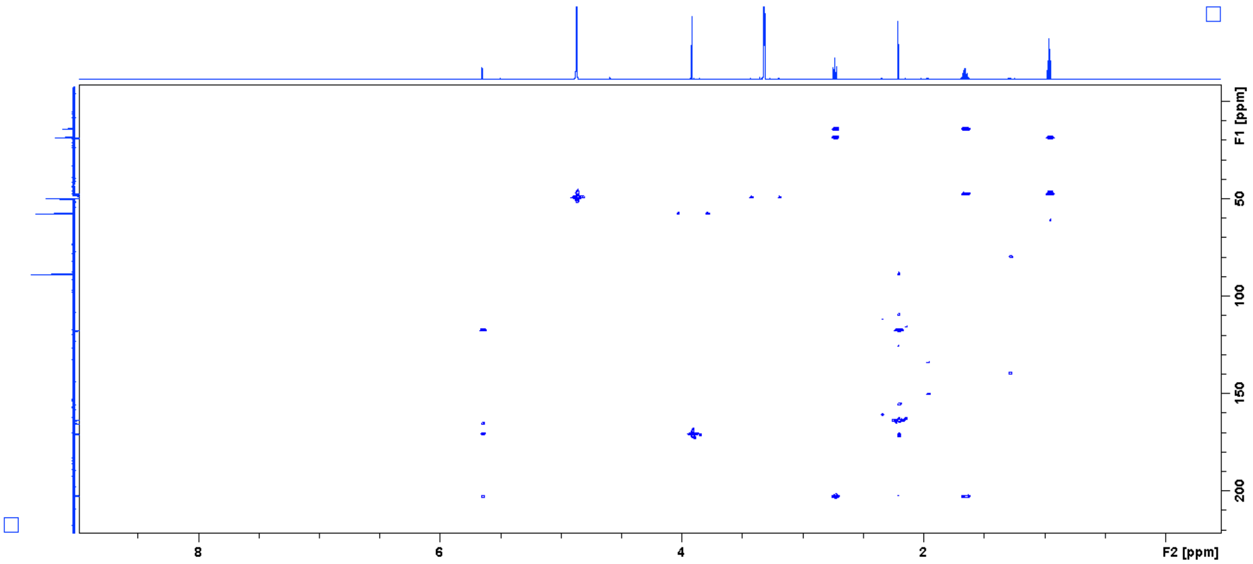

Figure S7. Toxic activity of pyrenocine A on *Ectocarpus siliculosus*. (A) Control with DMSO 1 % (B) Addition of pyrenocine A at 1 µg.mL^{-1}.

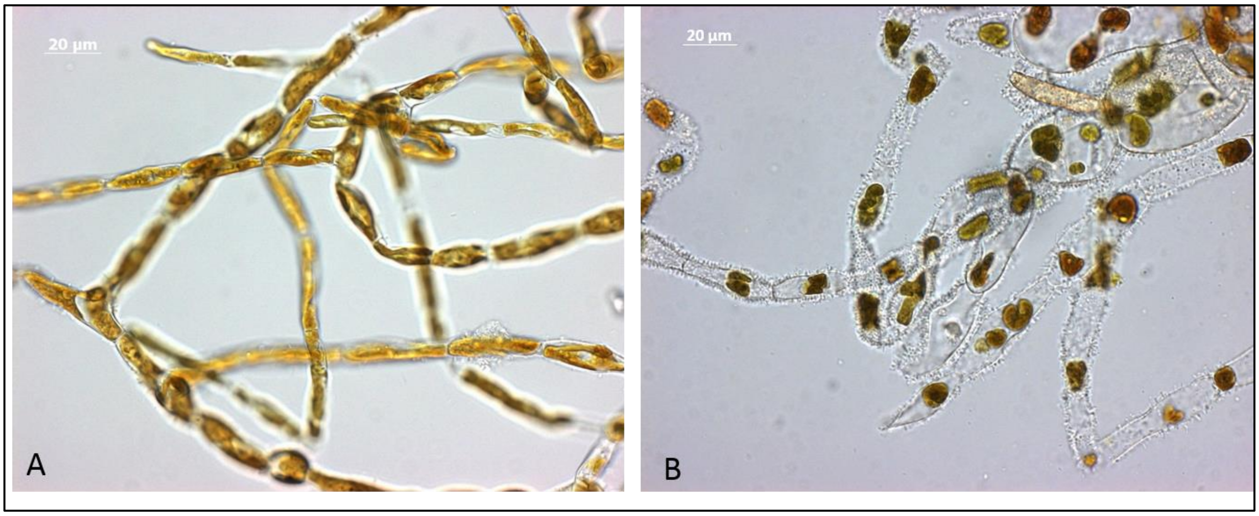

SI. Movie 1. Collapse of *Olpidiopsis* zoosporangia (cells highlighted by red arrows) few seconds after treatment with Pyrenochaetic acid C at 1 µg.mL^{-1}. Video speed was increased by 5.5 times.

movie.wmv

Alignment 1. 28 S

>Mucor_hiemalis_strain_DAOM225705_JN938893.1

ATTGGCCAGAGCTGTTGT-CTTTTGTGATACATTTT-
CAAAGAGTCAGGTTGTTTGGGAATGCAGCCTAAATTGGGTGGTAAATCTCACCTAAAGC
TAAATATTTGCGAGAGACC-
GATAGCGAACAAGTACCGTGAGGGAAAGATGAAAAGAACTTTGAAAAGAGAGTTAAAC
AGTATGTGAAATTGTTAAAAGGGAACCGTTTGGAGCCAGACTGGCTTAATCGTAATCAA
TCTAGGCTTTGGCCTGGATGCACTTGCGGTTTATGCCGGCCAACGACAGTTTTGTTTGGG
AGAAAAAATTACATTGAATGTGGCCCCTC----GGG--GTGTTATAGCTTTGTAAAAAATAT-
CCTGGACTGGACTGAGGAACGCGGTTGTTG

>Mucor_hiemalis_isolate_LD450H

ATTGGCCAGAGCTGTTGT-CTTTTGTGATACATTTT-
CAAAGAGTCAGGTTGTTTGGGAATGCAGCCTAAATTGGGTGGTAAATCTCACCTAAAGC
TAAATATTTGCGAGAGACC-
GATAGCGAACAAGTACCGTGAGGGAAAGATGAAAAGAACTTTGAAAAGAGAGTTAAAC
AGTATGTGAAATTGTTAAAAGGGAACCGTTTGGAGCCAGACTGGCTTAATCGTAATCAA
TCTAGGCTTTGGCCTGGATGCACTTGCGGTTTATGCCGGCCAACGACAGTTTTGTTTGGG
AGAAAAAATTACATTGAATGTGGCCCCTC----GGG--GTGTTATAGCTTTGTAAAAAATAT-
CCTGGACTGGACTGAGGAACGCGGTTGTTG

>Trametes_versicolor_strain_C-4_DQ208417.1

TTTGACACGGACTACCAATGCTTTGTGATGCGCTCT-
CAAAGAGTCGCGTTGTTTGGGAATGCAGCGCAAAATGGGAGGTGAATTCCTTCTAAAGC
TAAATATTGGCGAGAGACC-
GATAGCGAACAAGTACCGTGAGGGAAAGATGAAAAGCACTTTGGAAAGAGAGTTAAAC
AGTACGTGAAATTGCTAAAAGGGAAACGCTTGAAGTCAGTCGCGTCGTCCGGAACTCAG
CTTTGCTTC--GGCTTAGTGCACTT-
TCCGGTTGACGGGCCAGCATCGATTTTGACCGCTGGAAAAGGGCTGGAGGAATGTGGCA
CCTTC--GGGT--GTGTTATAGCCTTCAGTCGCATAC-
AGCGGTTGGGATCGAGGAACGCGGATGCTG

>Trametes_versicolor_isolate_AN131R

TT-GACACGGACTACCAATGCTTTGTGATGCGCTCT-
CAAAGAGTCGCGTTGTTTGGGAATGCAGCGCAAAATGGGAGGTGAATTCCTTCTAAAGC
TAAATATTGGCGAGAGACC-
GATAGCGAACAAGTACCGTGAGGGAAAGATGAAAAGCACTTTGGAAAGAGAGTTAAAC
AGTACGTGAAATTGCTAAAAGGGAAACGCTTGAAGTCAGTCGCGTCGTCCGGAACTCAG
CTTTGCTTC--GGCTTAGTGCACTT-
TCCGGTTGACGGGCCAGCATCGATTTTGACCGCTGGAAAAGGGCTGGAGGAATGTGGCA
CCTTC--GGGT--GTGTTATAGCCTTCAGTCGCATAC-
AGCGGTTGGGATCGAGGAACGCGGATGCTG

>Psathyrella_candolleana_strain_SZMC-NL-2937_FN396165.1

TTTGACACGGACTACCGAGGCTTTGTGGTATGCTCT-
CAAAGAGTCGAGTTGTTTGGGAATGCAGCTCAAAATGGGTGGTAAATTCCATCTAAAGC
TAAATATTGGCGAGAGACC-
GATAGCGAACAAGTACCGTGAGGGAAAGATGAAAAGAACTTTGGAAAGAGAGTTAAAC
AGTACGTGAAATTGCTGAAAGGGAAACGCTTGAAGTCAGTCGCGTTGGCCGGGAATCAG
CCTTGCTTT--TGCTTGGTGTACTT-
TCTGGTTGACGGGCCAGCATCAGTTTTGACCGGTGGAAAAAGTTCAGGGGAATGTGGCA
TCTTC--GGGT--GTGTTATAGCCCTTGTTCGTATAC-
ATCGGTTGGGACTGAGGAACTCGGATGCTG

>Psathyrella_candolleana_isolate_SL563F

TTTGACACGGACTACCGAGGCTTTGTGGTATGCTCT-
CAAAGAGTCGAGTTGTTTGGGAATGCAGCTCAAAATGGGTGGTAAATTCCATCTAAAGC
TAAATATTGGCGAGAGACC-
GATAGCGAACAAGTACCGTGAGGGAAAGATGAAAAGAACTTTGGAAAGAGAGTTAAAC
AGTACGTGAAATTGCTGAAAGGGAAACGCTTGAAGTCAGTCGCGTTGGCCGGGAATCAG
CCTTGCTTT--TGCTTGGTGTACTT-
TCTGGTTGACGGGCCAGCATCAGTTTTGACCGGTGGAAAAAGTTCAGGGGAATGTGGCA
TCTTC--GGGT--GTGTTATAGCCCTTGTTCGTATAC-
ATCGGTTGGGACTGAGGAACTCGGATGCTG

>Talaromyces_flavus_strain_CBS310.38_JF922044.1

TGGGAT--GGGCGGCCGCGCCCGTGTGAAGCTCCTT-
CGACGAGTCGAGTTGTTTGGGAATGCAGCTCTAAGCGGGTGGTAAATTTCATCTAAAGC
TAAATACTGGCCGGAGACC-
GATAGCGCACAAGTAGAGTGATCGAAAGATGAAAAGCACTTTGAAAAGAGAGTTAAAC
AGCACGTGAAATTGTTGAAAGGGAAGCGTTGTCCACCAGACTCGCCCGGGGGGGTTCAG
CCGGCACTT--GTGCCGGTGTACTC-
CTCTCCGGGCGGGCCAGCATCGGTTTGGGCGGCTGGTGAAAGGCCCCGGGAATGTAACA
CCCCTCGGGGT--
GCCTTATAGCCCGGGGTGCCATACAGCCAGCCTGGACCGAGGCCCGCGGATGCTG

>Talaromyces_atricola_isolate_LD147H

TGGGAT--GGGTGGTCCCGCCCGTGTGAAGCTCCTT-
CGAAGAGTCGAGTTGTTTGGGAATGCAGCTCTAAGAGGGTGGTAAATTTCATCTAAAGC
TAAATATTGGCCGGAGACC-
GATAGCGCACAAGTAGAGTGATCGAAAGATGAAAAGCACTTTGAAAAGAGAGTTAAAC
AGCACGTGAAATTGTTGAAAGGGAAGCGTTGGCAACCAGACTCGCCCGGGAGGGCTCA
GCCGGCACGT--GTGCCGGTGCACTC-
CCCCCCGGGCGGGCCAGCGTCGGTTTGGGCGGTCGGTCAAAGGTCTTGGGAATGTAGCA
CCCTCCGGGGT--
GCCTTATAGCCCGAGGCGCCATGCGACCTGCCCGGACCGAGGAACGCGGACGCTG

>Aspergillus_protuberus_isolate_AFTOL-ID5007_FJ176897.1

TTGGGCA-GGGTGCCCGTGCCCGTGTGAAGCTCCTT-
CGACGAGTCGAGTTGTTTGGGAATGCAGCTCTAAATGGGTGGTAAATTTCATCTAAAGC
TAAATACCGGCCGGAGACC-
GATAGCGCACAAGTAGAGTGATCGAAAGATGAAAAGCACTTTGAAAAGAGAGTTAAAC
AGCACGTGAAATTGTTGAAAGGGAAGCGCTTGCGACCAGACTCGGCCTCG-
GGGTTCAGCCAGCATTC--GTGCTGGTGTACTT-
CCCCGGGGCCGGGCCAGCGTCGGTTCGGGCGGCCGGTCAAAGGCCCCAGGAATGTATCG
TCCTCCGGGAC--
GTCTTATAGCCTGGGGTGCAATGCGGCCAGCCTGGACCGAGGAACGCGGACGCTG

>Aspergillus_versicolor_isolate_LD294F

TTGGGCA-GGGTGCCCGTGCCCGTGTGAAGCTCCTT-
CGACGAGTCGAGTTGTTTGGGAATGCAGCTCTAAATGGGTGGTAAATTTCATCTAAAGC
TAAATACCGGCCGGAGACC-
GATAGCGCACAAGTAGAGTGATCGAAAGATGAAAAGCACTTTGAAAAGAGAGTTAAAC
AGCACGTGAAATTGTTGAAAGGGAAGCGCTTGCGACCAGACTCGGCCTCG-
GGGTTCAGCCAGCATTC--GTGCTGGTGTACTT-
CCCCGGGGCCGGGCCAGCGTCGGTTCGGGCGGCCGGTCAAAGGCCCCAGGAATGTATCG
TCCTCCGGGAC--
GTCTTATAGCCTGGGGTGCAATGCGGCCAGCCTGGACCGAGGAACGCGGACGCTG

>Penicillium_osmophilum_strain_CBS439.73_JF922036.1

TGGGATG-GGGTGTCCGCGCCCGTGTGAAGCTCCTT-
CGACGAGTCGAGTTGTTTGGGAATGCAGCTCTAAATGGGTGGTAAATTTCATCTAAAGC
TAAATATTGGCCGGAGACC-
GATAGCGCACAAGTAGAGTGATCGAAAGATGAAAAGCACTTTGAAAAGAGAGTTAAAA
AGCACGTGAAATTGTTGAAAGGGAAGCGCTTGCGACCAGACTCGCTCGCG-
GGGTTCAGCCGGCATTC--GTGCCGGTGTACTT-
CCCCGCGGGCGGGCCAGCGTCGGTTTGGGCGGTCGGTCAAAGGCCCTCGGAAGGTAACG
CCCCCAGGGGC--
GTCTTATAGCCGAGGGTGCAATGCGACCTGCCTAGACCGAGGAACGCGGACGCTG

>Penicillium_echinatum_isolate_LD69H

TGGGATG-GGGTGTCCGCGCCCGTGTGAAGCTCCTT-
CGACGAGTCGAGTTGTTTGGGAATGCAGCTCTAAATGGGTGGTAAATTTCATCTAAAGC
TAAATATTGGCCGGAGACC-
GATAGCGCACAAGTAGAGTGATCGAAAGATGAAAAGCACTTTGAAAAGAGAGTTAAAA
AGCACGTGAAATTGTTGAAAGGGAAGCGCTTGCGACCAGACTCGCTCGCG-
GGGTTCAGCCGGCATTC--GTGCCGGTGTACTT-
CCCCGCGGGCGGGCCAACGTCGGTTTGGGCGGTCGGTCAAAGGCCCTCGGAAGGTAACG
CCCCCAGGGGC--
GTCTTATAGCCGAGGGTGCAATGCGACCTGCCTAGACCGAGGAACGCGGACGTTG

>Cadophora_luteo-olivacea_strain_ICMP:18084_HM116758.1

TGTGACT-GGGTGCTTTCGCTCATGTGAAGCTCTTT-
CGACGAGTCGAGTTGTTTGGGAATGCAGCTCAAAATGGGTGGTAAATTTCATCTAAAGC
TAAATATTGGCCAGAGACC-
GATAGCGCACAAGTAGAGTGATCGAAAGATGAAAAGCACTTTGGAAAGAGAGTTAAAC
AGTACGTGAAATTGTTGAAAGGGAAGCGCTTGCAACCAGACTTGCGCGCTGATGATCAT
CCGGGCTTC--TGCCCGGTGCACTC-
GTCTGCGCTCAGGCCAGCATCGGTTTCGGTGGTGGGATAAAGGCCTTGGGAATGTAGCT
CCTCTCGGGGA--
GTGTTATAGCCCTCGGTGCAATGCCGCCTACCGGGACCGAGGACCGCGGATGCTG

>Cadophora_luteo-olivacea_isolate_SL408T

TGTGACT-GGGTGCTTTCGCTCATGTGAAGCTCTTT-
CGACGAGTCGAGTTGTTTGGGAATGCAGCTCAAAATGGGTGGTAAATTTCATCTAAAGC
TAAATATTGGCCAGAGACC-
GATAGCGCACAAGTAGAGTGATCGAAAGATGAAAAGCACTTTGGAAAGAGAGTTAAAC
AGTACGTGAAATTGTTGAAAGGGAAGCGCTTGCAACCAGACTTGCGCGCTGATGATCAT
CCGGGCTTC--TGCCCGGTGCACTC-
GTCTGCGCTCAGGCCAGCATCGGTTTCGGTGGTGGGATAAAGGCCTTGGGAATGTAGCT
CCTCTCGGGGA--
GTGTTATAGCCCTCGGTGCAATGCCGCCTACCGGGACCGAGGACCGCGGATGCTG

>Botryotinia_fuckeliana_isolate_AFTOL-ID59_AY544651.1

TGTGACT-GGATACCTATGCTCATGTGAAGCTCTTT-
CGACGAGTCGAGTTGTTTGGGAATGCAGCTCAAAATGGGAGGTATATTTCTTCTAAAGC
TAAATATTGGCCAGAGACC-
GATAGCGCACAAGTAGAGTGATCGAAAGATGAAAAGCACTTTGGAAAGAGAGTTAAAC
AGTACGTGAAATTGTTGAAAGGGAAGCGCTTGCAATCAGACTTGCAC-
TTGGTGTTCATCAGGGTCTCG-TACCCTGTGTACTT-
CATCAAGTTCAGGCCAGCATCAGTTTGAGTGGTTAGATAAAGGCTTAGAGAATGTGGCC
CTCTTCGGGGG--
GTGTTATAGCTCTAGGTGCAATGTAGCCTACTTGGACTGAGGACCGCGGATGCTG

>Botryotinia_fuckeliana_isolate_SL474T

TGTGACT-GGATACCTATGCTCATGTGAAGCTCTTT-
CGACGAGTCGAGTTGTTTGGGAATGCAGCTCAAAATGGGAGGTATATTTCTTCTAAAGC
TAAATATTGGCCAGAGACC-
GATAGCGCACAAGTAGAGTGATCGAAAGATGAAAAGCACTTTGGAAAGAGAGTTAAAC
AGTACGTGAAATTGTTGAAAGGGAAGCGCTTGCAATCAGACTTGCAC-
TTGGTGTTCATCAGGGTCTCG-TACCCTGTGTACTT-
CATCAAGTTCAGGCCAGCATCAGTTTGAGTGGTTAGATAAAGGCTTAGAGAATGTGGCC
CTCTTCGGGGG--
GTGTTATAGCTCTAGGTGCAATGTAGCCTACTTGGACTGAGGACCGCGGATGCTG

>Pilidium_concavum_voucher_BPI1107274_AY487098.1

CGTGGTC-GGCTGCTGGC-TTCGTGTAAAGCTCCTT-
CGACGAGTCGAGTTGTTTGGGAATGCAGCTCAAAATGGGTGGTAAATTTCATCTAAAGC
TAAATATTGGCCAGAGACC-
GATAGCGCACAAGTAGAGTGATCGAAAGATGAAAAGCACTTTGGAAAGAGAGTTAAAC
AGCACGTGAAATTGTTGAAAGGGAAGCGCTTGCAATCAGACTTGCGCGTAGGTAATCAA
GGGCGGTTCGCTGCCC--TGCACTT-
GCCTGCGCTCAGGCCAGCATCAGTTTTGGGGGTGGGATAAAGGCCGTGGGAATGTGGCT
CTTC----GGA--
GTGTTATAGCCCTCGGTGCCATGCCGCCTCCCGAGACTGAGGACCGCGGATGCTG

>Pilidium_concavum_isolate_LD482H

CGTGGTC-GGCTGCCTGC-TTCGTGTAAAGCTCCTT-
CGACGAGTCGAGTTGTTTGGGAATGCAGCTCAAAATGGGTGGTAAATTTCATCTAAAGC
TAAATATTGGCCAGAGACC-
GATAGCGCACAAGTAGAGTGATCGAAAGATGAAAAGCACTTTGGAAAGAGAGTTAAAC
AGCACGTGAAATTGTTGAAAGGGAAGCGCTTGCAATCAGACTTGCGCGTAGGTAATCAA
GGGCGGTTCGCTGCCC--TGCACTT-
GCCTGCGCTCAGGCCAGCATCAGTTTTGGGGGTGGGATAAAGGCCGTGGGAATGTGGCT
CTTC----GGA--
GTGTTATAGCCCTCGGTGCCATGCCGCCTCCCGAGACTGAGGACCGCGGATGCTG

>Libertella_blepharis_isolate_LBAg_AY621003.1

CG--GTT--GGACACTAAGCCTCTGTAAAGCTCCTT-
CGACGAGTCGAGTAGTTTGGGAATGCTGCTCTAAATGGGAGGTAAATTTCTTCTAAAGC
TAAATACCGGCCAGAGACC-
GATAGCGCACAAGTAGAGTGATCGAAAGATGAAAAGTACTTTGAAAAGAGGGTTAAAT
AGCACGTGAAATTGTTGAAAGGGAAGCGTTTATGACCAGACCTTTGCCGGGCGGATCAT
CCGGTGTTCT-TCACCGGTGCACTT-
CGCTCGGCTTAGGCCAGCATCGATTTCGGCAGGGGGACAAAGACCATGGGAACGTAGCT
CTCTTCGGGGA--GTGTTATAGCCCTAGGTGTAATAC-
CCTTGCGGGGATCGAGGTTCGCGGATGCTG

>Eutypella_scoparia_isolate_LD481H

CG--GTT--GGACACCAAGCCTCTGTAAAGCTCCTT-
CGACGAGTCGAGTAGTTTGGGAATGCTGCTCTAAATGGGAGGTAAATTTCTTCTAAAGC
TAAATACCGGCCAGAGACC-
GATAGCGCACAAGTAGAGTGATCGAAAGATGAAAAGTACTTTGAAAAGAGGGTTAAAT
AGCACGTGAAATTGTTGAAAGGGAAGCGTTTATGACCAGACCTTCGCCGGGCGGATCAT
CCGGTGTTCT-TCACCGGTGCACTT-
CGCCCGGCTTAGGCCAGCATCGGTTTTCGCAGGGGGATAAAGGCCCCGGGAACGTAGCT
CCCTCCGGGGA--GTGTTATAGCCCGGGACGTAATAC-
CCTTGCGGGGACCGAGGTTCGCGGATGCTG

>Eutypa_lata_isolate_AFTOL-ID929_DQ836903.1

CG--GTT--GGACACTAAGCCTCTGTAAAGCTCCTT-
CGACGAGTCGAGTAGTTTGGGAATGCTGCTCTAAATGGGAGGTAAATTTCTTCTAAAGC
TAAATACCGGCCAGAGACC-
GATAGCGCACAAGTAGAGTGATCGAAAGATGAAAAGTACTTTGAAAAGAGGGTTAAAT
AGCACGTGAAATTGTTGAAAGGGAAGCGTTTATGACCAGACCTTTGCCGGGCGGATCAT
CCGGTGTTCT-TCACCGGTGCACTT-
CGCTCGGCTTAGGCCAGCATCGATTTCTGGAGGGGGACAAAGACCATGGGAACGTAGCT
CTCTTCGGGGA--GTGTTATAGCCCTAGGTGTAATAC-
CCTTCCGGGGATCGAGGTTCGCGGATGCTG

>Eutypa_lata_isolate_LD366H

CG--GTT--GGACACCAAGCCCGTGTAAAGCTCCTT-
CGACGAGTCGAGTAGTTTGGGAATGCTGCTCTAAATGGGAGGTAAATTTCTTCTAAAGC
TAAATACCGGCCAGAGACCCGATAGCGCACAAGTAGAGTGATCGAAAGATGAAAAGTA
CTTTGAAAAGAGGGTTAAATAGCACGTGAAATTGTTGAAAGGGAAGCGTTTATGACCAG
ACCTTTGTCCGGCGGATCATCCGGTGTTCT-TCACCGGTGCACTT-
CGCCGGGCTCAGGCCAGCATCGATTTCCGTAGGGGGATAAAGGCCATGGGAACGTAGCT
CTCTTCGGGGA--GTGTTATAGCCCTAGGTGTAATAC-
CCTTATGGGGATCGAGGTTCGCGGATGCTG

>Pestalotiopsis_sp._strain_MA177_GU592017.1

CG--GTT--GAATGCCTAGCCTCTGTAAATCTCCTT-
CGACGAGTCGAGTAGTTTGGGAATGCTGCTCTAAATGGGAGGTAAATTTCTTCTAAAGC
TAAATATTGGCCAGAGACC-
GATAGCGCACAAGTAGAGTGATCGAAAGATGAAAAGCACTTTGAAAAGAGGGTTAAAT
AGCACGTGAAATTGTTGAAAGGGAAGGATTTGTGACCAGACTTTTTCTGGGCGGATCAT
CCGGGGTTCT--CTCCGGTGCACTT-
CGCCCAGTAAAGGCCAGCATCGGTTTTCGGCGTGGGATAAAAGCAGTAGGAATGTGGCT
CTCTACGGGGA--GTGTTATAGCCTATTGTATAATAC-
CGCGCTGGGGACCGAGGTTCGCGGATGCTG

>Pestialotiopsis_microspora_isolate_AN325T

CG--GTT--GAATGCCTAGCCTCTGTAAATCTCCTT-
CGACGAGTCGAGTAGTTTGGGAATGCTGCTCTAAATGGGAGGTAAATTTCTTCTAAAGC
TAAATATTGGCCAGAGACC-
GATAGCGCACAAGTAGAGTGATCGAAAGATGAAAAGCACTTTGAAAAGAGGGTTAAAT
AGCACGTGAAATTGTTGAAAGGGAAGGATTTGTGACCAGACTTTTTCTGGGCGGATCAT
CCGGGGTTCT--CTCCGGTGCACTT-
CGCCCAGTAAAGGCCAGCATCGGTTTTCGGCGCGGGATAAAAGCGGTAGGAATGTGGCT
CTCTACGGGGA--GTGTTATAGCCTATCGTATAATAC-
CGCGCTGAGGACCGAGGTTCGCGGATGCTG

>Cladosporium_cladosporioides_isolate_C1/16_KM246047.1

TGCGGTC--GGAAAGGTGCTCTACACGTAGCTCCTT-
CGACGAGTCGAGTTGTTTGGGAATGCAGCTCTAAATGGGAGGTAAATTTCTTCTAAAGC

TAAATATTGGCCAGAGACC-
GATAGCGCACAAGTAGAGTGATCGAAAGATGAAAAGCACTTTGGAAAGAGAGTTAAAA
AGCACGTGAAATTGTTAAAAGGGAAGGGATTGCAACCAGACTTGCTCGCGG-
TGTTCCGCCGGTCTTC--TGACCGGTCTACTC-
GCCGCGTTGCAGGCCAGCATCGTCTGGTGCCGCTGGATAA-
GACTTGAGGAATGTAGCTCCCTC--GGGA--GTGTTATAGC--
CTCTTGTGATGCAGCGAGCGCCGGGCGAGGTCCGCGGATGCTG

>Cladosporium_cucumerinum_isolate_AN187R

TGCGGTC--GGAAAGGCGCTCTATACGTAGCTCTTT-
CGACGAGTCGAGTTGTTTGGGAATGCAGCTCTAAATGGGAGGTAAATTTCTTCTAAAGC
TAAATATTGGCCAGAGACC-
GATAGCGCACAAGTAGAGTGATCGAAAGATGAAAAGCACTTTGGAAAGAGAGTTAAAA
AGCACGTGAAATTGTTAAAAGGGAAGGGATTGCAACCAGACTTGCTCGCGG-
TGTTCCGCCGGTCTTC--TGACCGGTCTACTC-
GCCGCGTTGCAGGCCAGCATCGTCTGGTGCCGCTGGATAA-
GACTTGAGGAATGTAGCTCCCTC--GGGA--GTGTTATAGC--
CTCTTGTGATGCAGCGAGCGCCGGGCGAGGTCCGCGGATGCTG

>Mycophycias_ascophylli_isolate_FR06_HQ412305.1

CGGGACC--GGCT-GGCACCTTCCACGTAGCTCCTT-
CGACGAGTCGAGTTGTTTGGGAATGCAGCTCTAAATGGGAGGTAAATTTCTTCTAAAGC
TAAATACCGGCCAGAGACC-
GATAGCGCACAAGTAGAGTGATCGAAAGATGAAAAGCACTTTGGAAAGAGAGTTAAAC
AGCACGTGAAATTGTTGAAAGGGAAGCGCTTGCAACCAGACTTGTCGGCGG-
CGTCCCCCTGGTCTTCT--GCCCAGGGAGC---
AGCCGCCGGTAGGCCAGCATCGTCCGGGGCCGCCGGACAAAGGCGTCGGGAATGTGGC
TCCCCTCGGGGA--
GCGTTATAGCCCGGCGCGCAATACGGTGCGTCCCGGGCGAGGTCCGCGGATGTTG

>Beauveria_bassiana_4JX481967.1

TG--GTC--GGACACCGAGCCTCTGTAAAGCTCCTT-
CGACGAGTCGAGTAGTTTGGGAATGCTGCTCAAAATGGGAGGTATATGTCTTCTAAAGC
TAAATATTGGCCAGAGACC-
GATAGCGCACAAGTAGAGTGATCGAAAGATGAAAAGCACTTTGAAAAGAGGGTTAAAA
AGTACGTGAAATTGTTGAAAGGGAAGCGCCTATGACCAGACTTGCGCCCGGTGAATCAC
CCAGCGTTCT--CGCTGGTGCACTT-
TGCCGGGCACAGGCCAGCATCAGTTCAGCGCGGGGGAGAAAGGCTTCGGGAATGTGGC
TCCCTC--GGGA--GTGTTATAGCCCGCTGCGTAATGC-
CCTGCGCCGGACTGAGGTACGCGGATGCTG

>Verticillium_cf._biguttattum_isolate_AN130T

TG--GTC--GGACACCGAGCCTCTGTAAAGCTCCTT-
CGACGAGTCGAGTAGTTTGGGAATGCTGCTCAAAATGGGAGGTATATGTCTTCTAAAGC

TAAATATTGGCCAGAGACC-
GATAGCGCACAAGTAGAGTGATCGAAAGATGAAAAGCACTTTGAAAAGAGGGTTAAAA
AGTACGTGAAATTGTTGAAAGGGAAGCGCCTATGACCAGACTTGGGCCCGGTGAATCAT
CCAGCGTTCT--CGCTGGTGCACTT-
TGCCGGGCACAGGCCAGCATCAGTTTGGCGCGGGGAAAAAGGCTTCGGGAATGTGGC
TCCCTC--GGGA--GTGTTATAGCCCGCTGCGTAATAC-
CCTGCGCCGGACTGAGGTACGCGGATGCTG

>Hypocrea_lixii_strain_CBS226.95_HM466680.1

TG--GCT--GGCCGCCGAGCCTCTGTAAAGCTCCTT-
CGACGAGTCGAGTAGTTTGGGAATGCTGCTCAAAATGGGAGGTATATGTCTTCTAAAGC
TAAATATTGGCCAGAGACC-
GATAGCGCACAAGTAGAGTGATCGAAAGATGAAAAGCACCTTGAAAAGAGGGTTAAAT
AGTACGTGAAATTGTTGAAAGGGAAGCGCTTGTGACCAGACTTGGGCGCGGCGGATCAT
CCGGGGTTCT--CTCCGGTGCACTT-
CGCCGCGTCTAGGCCAGCATCAGTTCGTCGCGGGGGAAAAAGGCTTCGGGAACGTGGCT
CCTCC--GGGA--GTGTTATAGCCCGTTGCATAATAC-
CCTGCGGTGGACTGAGGACCGCGGATGCTG

>Hypocrea_lixii_isolate_LD8H

TG--GCT--GGCCGCCGAGCCTCTGTAAAGCTCCTT-
CGACGAGTCGAGTAGTTTGGGAATGCTGCTCAAAATGGGAGGTATATGTCTTCTAAAGC
TAAATATTGGCCAGAGACC-
GATAGCGCACAAGTAGAGTGATCGAAAGATGAAAAGCACCTTGAAAAGAGGGTTAAAT
AGTACGTGAAATTGTTGAAAGGGAAGCGCTTGTGACCAGACTTGGGCGCGGCGGATCAT
CCGGGGTTCT--CTCCGGTGCACTT-
CGCCGCGTCTAGGCCAGCATCAGTTCGTCGCGGGGGAAAAAGGCTTCGGGAACGTGGCT
CCTCC--GGGA--GTGTTATAGCCCGTTGCATAATAC-
CCTGCGGTGGACTGAGGACCGCGGATGCTG

>Tolypocladium_inflatum_AF373286.1

TG--GTC--GGACGCCAAGCCAGTGTAAAGCTCCTT-
CGACGAGTCGAGTAGTTTGGGAATGCTGCTCTAAATGGGAGGTATATGTCTTCTAAAGC
TAAATATAGGCCAGAGACC-
GATAGCGCACAAGTAGAGTGATCGAAAGATGAAAAGCACTTTGAAAAGAGGGTTAAAC
AGTACGTGAAATTGTTGAAAGGGAAGCACTTGTGACCAGACTTGGGCCCGGTGAATCAT
CCAGCGTTCT--CGCTGGTGCACTT-
CGCCGGGCCCAGGCCAGCATCAGTTCGCCGCGGGGGATAAAAGCTTCGGGAACGTAGCT
CCCTC--GGGA--GTGTTATAGCCCGTTGCATAATAC-
CCTGCGGTGGACTGAGGTTCGCGGATGCTG

>Tolypocladium_cylindrosporum_isolate_LD150F

-G--GTC--GGACGC-AAGCCAGTGTAAAGCTCCTT-
CGACGAGTCGAGTAGTTTGGGAATGCTGCTCTAAATGGGAGGTATATGTCTTCTAAAGC
TAAATATAGGCCAGAGACC-

GATAGCGCACAAGTAGAGTGATCGAAAGATGAAAAGCACTTTGAAAAGAGGGTTAAAC
AGTACGTGAAATTGTTGAAAGGGAAGCACTTGTGACCAGACTTGGGCCCGGTGAATCAT
CCAGCGTTCT--CGCTGGTGCACTT-
CGCCGGGCTCAGGCCAGCATCAGTTCGCCGCGGGGGACAAAAGCTTCGGGAACGTGGCT
CCCTC--GGGA--GTGTTATAGCCCGTTGCACAATAC-
CCTGCGGTGGACTGAGGTTCGCGGATGCTG

>Phialemoniopsis_ocularis_strain_CBS110031_KJ573449.1

AA--GTT--GGACACCAAGCCTATGTAAAGCTCCTT-
CGACGAGTCGAGTAGTTTGGGAATGCTGCTCAAAATGGGAGGTAAATTTCTTCTAAAGC
TAAATACCGGCCAGAGACC-
GATAGCGCACAAGTAGAGTGATCGAAAGATGAAAAGCACTTTGAAAAGAGGGTTAAAA
AGTACGTGAAATTGTTGAAAGGGAAGCGCTCATGACCAGACTTGTGCCCTGCGGATCAT
CCAGCCTTCT--GGCTGGTGCACTT-
CGCTTGGTTCAGGCCAGCATCGGTTCTCCGGGGGGGATAAAAGCTTCAGGAACGTAGCT
CCCTC--GGGA--GCATTATAGCCTGTTGCATAATAC-
CCTCTAGGGGACCGAGGTTCGCGGATGCTG

>Sarcopodium_brongniarti_isolate_LD185H

AA--GTT--GGACACCAAGCCTATGTAAAGCTCCTT-
CGACGAGTCGAGTAGTTTGGGAATGCTGCTCAAAATGGGAGGTAAATTTCTTCTAAAGC
TAAATACCGGCCAGAGACC-
GATAGCGCACAAGTAGAGTGATCGAAAGATGAAAAGCACTTTGAAAAGAGGGTTAAAC
AGTACGTGAAATTGTTGAAAGGGAAGCGCTCATGACCAGACTTGTGCCCTGCGAATCAT
CCAGCCTTCT--GGCTGGTGCACTT-
CGCTTGGTTCAGGCCAGCATCGGTTTTCTGAGGGGGATAAAAGCTTCAGGAACGTAGCT
CCTCC--GGGA--GCATTATAGCCTGTTGCATAATAC-
CCCTCGGGGGACCGAGGTTCGCGGATGCTG

>Diaporthe_phaseolorum_AY346279.1

TG--GTC--GGACACCAAGCCTGTGTGAAGCTCCTT-
CGACGAGTCGAGTAGTTTGGGAATGCTGCTCTAAATGGGAGGTAAATCTCTTCTAAAGC
TAAATACCGGCCAGAGACC-
GATAGCGCACAAGTAGAGTGATCGAAAGATGAAAAGCACCTTGAAAAGGGGGTTAAAT
AGTACGTGAAATTGTTGAAAGGGAAGCACTTATGACCAGACTTGGGCCGGGCGGCTCAT
CAGGGGTTCT--CCCCTGTGCACTC-
CGCCCGGCACAGGCCAGCATCGGTTTTCGCGGGGGATAAGACCGACGGGAACGTAGC
ACCCTCCGGGGT--GTGTTATAGCCCGGCGGACGATAC-
CCTCGCGGGGACCGAGGACCGCGGATGCTG

>Diaporthe_globosum_isolate_LD46H

TG--GTT--GGACACCAAGCCTGTGTGAAACTCCTT-
CGACGAGTCGAGTAGTTTGGGAATGCTGCTCTAAATGGGAGGTAAATCTCTTCTAAAGC
TAAATACCGGCCAGAGACC-

GATAGCGCACAAGTAGAGTGATCGAAAGATGAAAAGCACCTTGAAAAGGGGGTTAAAT
AGTACGTGAAATTGTTGAAAGGGAAGCACTTATGACCAGACTTGGGCCGGGCGGTTCAT
CAGGGGTTCT--CCCCTGTGCACTC-
CGTCCGGCACAGGCCAGCATCGGTTTTCGTGGGGGGATAAGACCGTCGGGAACGTAGCA
CCCTCCGGGGT--GTGTTATAGCTCGGCGGACGATAC-
TCTCGCGGGGACCGAGGTTCGCGGATGCTG

>Diaporthe_oncostoma_strain_AR3445_AF408353.1

TG--GTC--GGACACCAAGCCTGTGTGAAGCTCCTT-
CGACGAGTCGAGTAGTTTGGGAATGCTGCTCTAAATGGGAGGTAAATCTCTTCTAAAGC
TAAATACCGGCCAGAGACC-
GATAGCGCACAAGTAGAGTGATCGAAAGATGAAAAGCACCTTGAAAAGGGGGTTAAAT
AGTACGTGAAATTGTTGAAAGGGAAGCACTTATGACCAGACTTGGGCCGGGCGGCTCAT
CAGGGGTTCT--CCCCTGTGCACTC-
CGCCCGGCACAGGCCAGCATCGGTTCTCGTGGGGGGATAAGACCGACAGGAACGTAGC
ACCCTCCGGGGT--GTGTTATAGCCTGGCGGACGATAC-
CCCCGTGGGGACCGAGGTCCGCGGATGCTG

>Diaporthe_eres_isolate_SL473T

TG--GTC--GGACACCAAGCCTGTGTGAAGCTCTTT-
CGACGAGTCGAGTAGTTTGGGAATGCTGCTCTAAATGGGAGGTAAATCTCTTCTAAAGC
TAAATACCGGCCAGAGACC-
GATAGCGCACAAGTAGAGTGATCGAAAGATGAAAAGCACCTTGAAAAGGGGGTTAAAT
AGTACGTGAAATTGTTGAAAGGGAAGCACTTATGACCAGACTTGGGCCGGGCGGCTCAT
CAGGGGTTCT--CCCCTGTGCACTC-
CGCCCGGCACAGGCCAGCATCGGTTCTCGTGGGGGGATAAGACCGTCAGGAACGTAGC
ACCCTCCGGGGT--GTGTTATAGCCTGGCGGACGATAC-
CCCCGTGGGGACCGAGGTCCGCGGATGCTG

>Melanconis_stilbostoma_strain_AR3501_AF408374.1

TG--GTT--GGACACCAAGCCTGTGTAATACTCCTT-
CGACGAGTCGAGTAGTTTGGGAATGCTGCTCTAAATGGGAGGTAAATCTCTTCTAAAGC
TAAATACCGGCCAGAGACC-
GATAGCGCACAAGTAGAGTGATCGAAAGATGAAAAGCACCTTGAAAAGGGGGTTAAAC
AGTACGTGAAATTGTTGAAAGGGAAGCGTTTATGACCAGACTTGTGCCGTGTGGCTCAT
CCGGGGTTCT--CCCCGGTGCACTC-
CACACGGTTCAGGCCAACATCGGTTCTCGTTGGGGGATAAGAACAGTAGGAACGTGGCC
CTCTTCGGAGG--GTGTTATAGCCTATTGTACGATAC-
CTTGATGGGGACCGAGGACCGCGGATGTTG

>Melanconis_stilbostoma_isolate_SL428H

TG--GTT--GGACACCAAGCCTGTGTAATACTCCTT-
CGACGAGTCGAGTAGTTTGGGAATGCTGCTCTAAATGGGAGGTAAATCTCTTCTAAAGC
TAAATACCGGCCAGAGACC-
GATAGCGCACAAGTAGAGTGATCGAAAGATGAAAAGCACCTTGAAAAGGGGGTTAAAC

AGTACGTGAAATTGTTGAAAGGGAAGCGTTTATGACCAGACTTGTGCCGTGTGGCTCAT
CCGGGGTTCT--CCCCGGTGCACTC-
CATACGGTTCAGGCCAACATCGGTTCTCGTTGGGGGATAAGAACAGTAGGAACGTGGCC
CTCTTCGGAGG--GTGTTATAGCCTATTGTACGATAC-
CTTGATGGGGACCGAGGACCGCGGATGTTG

>Chaetomium_globosum_AY346272.1

TA--GTT--AGATGCCTAGCCTGTGTAAAGCTCCTT-
CGACGAGTCGAGTAGTTTGGGAATGCTGCTCAAAATGGGAGGTAAATTTCTTCTAAAGC
TAAATACCGGCCAGAGACC-
GATAGCGCACAAGTAGAGTGATCGAAAGATGAAAAGCACTTTGAAAAGAGGGTTAAAT
AGCACGTGAAATTGTTGAAAGGGAAGCGCTTGTGACCAGACTTGCGCCGGGCAGATCAT
CCGGTGTTCT--CACCGGTGCACTC-
TGCCCGGCTCAGGCCAGCATCGGTTCTCGCGGGGGGATAAAGGCCCTGGGAACGTAGCT
CCTCC--GGGA--GTGTTATAGCCCGGGGCGTAATGC-
CCTCGCGGGGACCGAGGTTCGCGGATGCTG

>Chaetomium_globosum_isolate_LD13H

TA--GTT--AGATGCCTAGCCTGTGTAAAGCTCCTT-
CGACGAGTCGAGTAGTTTGGGAATGCTGCTCAAAATGGGAGGTAAATTTCTTCTAAAGC
TAAATACCGGCCAGAGACC-
GATAGCGCACAAGTAGAGTGATCGAAAGATGAAAAGCACTTTGAAAAGAGGGTTAAAT
AGCACGTGAAATTGTTGAAAGGGAAGCGCTTGTGACCAGACTTGCGCCGGGCAGATCAT
CCGGTGTTCT--CACCGGTGCACTC-
TGCCCGGCTCAGGCCAGCATCGGTTCTCGCGGGGGGATAAAGGCCCTGGGAACGTAGCT
CCTCC--GGGA--GTGTTATAGCCCGGGGCGTAATGC-
CCTCGCGGGGACCGAGGTTCGCGGATGCTG

>Moheitospora_fruticosae_isolate_EF14_GU252145.1

CG--GTT--GGACCCCGAGCCTGTGTAAAGCTCCCT-
CGACGAGTCGAGTAGTTTGGGAATGCTGCTCTAATTGGGAGGTAAATTTCTTCTAAAGCT
AAATACCGGCCAGAGACC-
GATAGCGCACAAGTAGAGTGATCGAAAGATGAAAAGCACTTTGAAAAGAGGGTGAAAC
AGTACGTGAAATTGTTGAAAGGGAAGCGCTTGTGACCAGACCTGCGCCCGGTGAATCAT
CCAGC--TCC--GGCTGGTGCACTT-CTCCG-
GCGTAGGCCAGCACCGGTTTGACCCGGGGGAGAAAGACGGTGGGAATGTGGCTCT-AC--
G-GA--GTGTTATAGCCCGCCGTGTAATGC-CCTGGGACGGACCGAGGTACGCGGGTGCTG

>Moheitospora_sp._isolate_AN129R

CG--GTT--GGCCCCCGAGCCTGTGTAAAGCTCCTT-
CAACGAGTCGAGTAGTTTGGGAATGCTGCTCTAATTGGGAGGTAAATTTCTTCTAAAGCT
AAATACCGGCCAGAGACC-
GATAGCGCACAAGTAGAGTGATCGAAAGATGAAAAGCACTTTGAAAAGAGGGTGAAAC
AGTACGTGAAATTGTTGAAAGGGAAGCGCTTGTGATCAGACCTGCGCCCGGTGAATCAT

CCAGC--TCC--GGCTGGTGCACTT-CTCCG-
GCGTAGGCCAGCACCGGTTCACCGCGGGGGAAAAAGACGGCGGGAATGTGGCTCC-TC--
GTGA--GTGTTATAGCCCGCCGTGTAATGC-CCTGGGGTGGACCGAGGTACGCGGGTGCTG

>Paraphaeosphaeria_minitans_strain_CBS122786_EU754174.1

CGTGGTC--GCCTGCCTTTGCCGTGTAAAGCTCCTT-
CGACGAGTCGAGTTGTTTGGGAATGCAGCTCTAAATGGGAGGTAAATTTCTTCTAAAGC
TAAATACTGGCCAGAGACC-
GATAGCGCACAAGTAGAGTGATCGAAAGATGAAAAGTACTTTGGAAAGAGAGTCAAAA
AGCACGTGAAATTGTTGAAAGGGAAGCGCTTGCAGCCAGACTTGCCCGCAGTTGCTCAC
CTAGGCTTT--GGCCTGGGGCACTC-
TTCTGCGGGCAGGCCAGCATCAGTTTGGGCGGTTGGATAAAGGCCTCTGTCACGTATCTC
CCTTCGGGGT-GACCTTATAGG-
GGAGGCGCAATGCAACCAGCCCGGACTGAGGTCCGCGGATGCTG

>Paraphaeosphaeria_sporulosa_isolate_LD53H

CGTGGTC--GCCTGCCTTTGCCGTGTAAAGCTCCTT-
CGACGAGTCGAGTTGTTTGGGAATGCAGCTCTAAATGGGAGGTAAATTTCTTCTAAAGC
TAAATACTGGCCAGAGACC-
GATAGCGCACAAGTAGAGTGATCGAAAGATGAAAAGTACTTTGGAAAGAGAGTCAAAA
AGCACGTGAAATTGTTGAAAGGGAAGCGCTTGCAGCCAGACTTGCCCGCAGTTGCTCAC
CTAGGCTTT--GGCCTGGGGCACTC-
TTCTGCGGGCAGGCCAGCATCAGTTTGGGCGGTTGGATAAAGGCCTCTGTCACGTATCTC
CCTTCGGGGT-GACCTTATAGG-
GGAGGCGCAATGCAACCAGCCCGGACTGAGGTCCGCGGATGCTG

>Phaeosphaeria_vagams_isolate_SL539T

CGTGGTC--GCTAGCCTTCGCCGTGTAAAGCCCCTT-
CGACGAGTCGAGTTGTTTGGGAATGCAGCTCTAAATGGGAGGTAAATTTCTTCTAAAGC
TAAATACTGGCCAGAGACC-
GATAGCGCACAAGTAGAGTGATCGAAAGATGAAAAGCACTTTGGAAAGAGAGTCAAAT
AGCACGTGAAATTGTTGAAAGGGAAGCGCTTGCAGCCAGACTTGCCTGTAGTTGCTTAT
CCGGACTTT--TGTCCGGTGCACTC-
TTCTGCGGGCAGGCCAGCATCAGTTTAGGCGGTTGGATAAAGGTCTCTATCACGTACCTC
CCTTCGGGGTTGGCCTTATAGG-
GGAGACGACATGCAACCAGCCTGAACTGAGGTCCGCGGATGCTG

>Phaeosphaeria_oryzae_strain_CBS110110_GQ387591.1

CGTGGTC--GCTAGCCTTCGCCGTGTAAAGCCCCTT-
CGACGAGTCGAGTTGTTTGGGAATGCAGCTCTAAATGGGAGGTAAATTTCTTCTAAAGC
TAAATACTGGCCAGAGACC-
GATAGCGCACAAGTAGAGTGATCGAAAGATGAAAAGCACTTTGGAAAGAGAGTCAAAT
AGCACGTGAAATTGTTGAAAGGGAAGCGCTTGCAGCCAGACTTGCCTGTAGTTGCTTAT
CTGGACTTT--TGTCCGGTGCACTC-
TTCTGCGGGCAGGCCAGCATCAGTTTGGGCGGTTGGATAAAGGTCTCTGTCATGTACCTC

CTTTCGGGGA-GGCCTTATAGG-
GGAGACGACATGCAACCAGCCTGGACTGAGGTCCGCGGATGCTG

>Phaeosphaeria_sp._isolate_AN596H

CGTGGTC--GCTAGCTTTCGCCGTGTAAAGCCCTTT-
CGACGAGTCGAGTTGTTTGGGAATGCAGCTCTAAATGGGAGGTAAATTTCTTCTAAAGC
TAAATACTGGCCAGAGACC-
GATAGCGCACAAGTAGAGTGATCGAAAGATGAAAAGCACTTTGGAAAGAGAGTCAAAT
AGCACGTGAAATTGTTGAAAGGGAAGCGCTTGCAGCCAGACTTGCCTGTAGTTGCTTAT
CCGGACTTT--TGTCCGGTGCACTC-
TTCTGCGGGCAGGCCAGCATCAGTTTGGGCGGTTGGATAAAGGTCTCTGTCATGTACCTC
CTTTCGGGGA-GGCCTTATAGG-
GGAGACGACATGCAACCAGCCTGGACTGAGGTCCGCGGATGCTG

>Phaeosphaeria_poagena_strain_CBS136771_KJ869172.1

CGTGGTC--GCTAGCCTTCGCCGTGTAAAGCCCCTT-
CGACGAGTCGAGTTGTTTGGGAATGCAGCTCTAAATGGGAGGTAAATTTCTTCTAAAGC
TAAATATTGGCCAGAGACC-
GATAGCGCACAAGTAGAGTGATCGAAAGATGAAAAGCACTTTGGAAAGAGAGTCAAAT
AGCACGTGAAATTGTTGAAAGGGAAGCGCTTGCAGCCAGACTTGCCTGTAGTTGCTTAT
CTGGACTTT--TGTCCGGTGCACTC-
TTCTATAGGCAGGCCAGCATCAGTTTGGGCGGTTGGATAAAGGTCTCTGTCATGTACCTC
CTTTCGGGGA-GGCCTTATAGG-
GGAGACGACATGCAACCAGCCTGGACTGAGGTCCGCGGATGCTG

>Phaeosphaeria_poagena_isolate_SL470T

CGTGGTC--
GCTAGCCTTCGCCGTGTAAAGCCCCTTTCGACGAGTCGAGTTGTTTGGGAATGCAGCTCT
AAATGGGAGGTAAATTTCTTCTAAAGCTAAATATTGGCCAGAGACC-
GATAGCGCACAAGTAGAGTGATCGAAAGATGAAAAGCACTTTGGAAAGAGAGTCAAAT
AGCACGTGAAATTGTTGAAAGGGAAGCGCTTGCAGCCAGACTTGCCTGTAGTTGCTTAT
CTGGACTTT--TGTCCGGTGCACTC-
TTCTATAGGCAGGCCAGCATCAGTTTGGGCGGTTGGATAAAGGTCTCTGTCATGTACCTC
CTTTCGGGGA-GGCCTTATAGG-
GGAGACGACATGCAACCAGCCTGGACTGAGGTCCGCGGATGCTG

>Stagonospora_sp._strain_CBS516.74_KF251770.1

CGTGGTC--GCTAGCCTTGGCCGTGTAAAGCCCCTT-
CGACGAGTCGAGTTGTTTGGGAATGCAGCTCTAAATGGGAGGTAAATTTCTTCTAAAGC
TAAATACTGGCCAGAGACC-
GATAGCGCACAAGTAGAGTGATCGAAAGATGAAAAGCACTTTGGAAAGAGAGTCAAAT
AGCACGTGAAATTGTTGAAAGGGAAGCGCTTGCAGCCAGACTTGCCTGTAGTTGCTTAT
CTGGACTTT--TGTCCGGTGCACTC-
TTCTGCGGGCAGGCCAGCATCAGTTTGGGCGGTTGGATAAAGGTCTCTGTCATGTACCTC

CTTTCGGGGA-GGCCTTATAGG-
GGAGACGACATGCAACCAGCCTGGACTGAGGTCCGCGGATGCTG

>Phaeosphaeria_sp._isolate_SL472T

CGTGGTC--GCTAGCCTTCGCCGTGTAAAGCCCCTT-
CGACGAGTCGAGTTGTTTGGGAATGCAGCTCTAAATGGGAGGTAAATTTCTTCTAAAGC
TAAATACTGGCCAGAGACC-
GATAGCGCACAAGTAGAGTGATCGAAAGATGAAAAGCACTTTGGAAAGAGAGTCAAAT
AGCACGTGAAATTGTTGAAAGGGAAGCGCTTGCAGCCAGACTTGCCTGTAGTTGCTTAT
CTGGACTTT--TGTCCGGTGCACTC-
TTCTATAGGCAGGCCAGCATCAGTTTGGGCGGTTGGATAAAGGTCTCTGTCATGTACCTC
CTTTCGGGGA-GGCCTTATAGG-
GGAGACGACATGCAACCAGCCTGGACTGAGGTCCGCGGATGCTG

>Pyrenochaeta_quercina_strain_CBS297.74_GQ387620.1

CGTGGTC--GCTGGCCTTCGCCGTGTAAAGCCCCTT-
CGACGAGTCGAGTTGTTTGGGAATGCAGCTCTAAATGGGAGGTAAATTTCTTCTAAAGC
TAAATACTGGCCAGAGACC-
GATAGCGCACAAGTAGAGTGATCGAAAGATGAAAAGCACTTTGGAAAGAGAGTCAAAT
AGCACGTGAAATTGTTGAAAGGGAAGCGCTTGCAGCCAGACTTGCCTGTAGTTGCTCAT
CCGGGCTTT--TGCCCGGTGCACTC-
TTCTGCGGGCAGGCCAGCATCAGTCTGGGCGGTTGGATAAAGGCCTCTGTCATGTACCT
CCTCTCGGGGA-GGCCTTATAGG-
GGAGGCGTAATGCAACCAGCCTGGACTGAGGTCCGCGGATGCTG

>Ochrocladosporium_elatum_isolate_SL464T

CGTGGTC--GCTGGCCTTCGCCGTGTAAAGCCCCTT-
CGACGAGTCGAGTTGTTTGGGAATGCAGCTCTAAATGGGAGGTAAATTTCTTCTAAAGC
TAAATACTGGCCAGAGACC-
GATAGCGCACAAGTAGAGTGATCGAAAGATGAAAAGCACTTTGGAAAGAGAGTCAAAT
AGCACGTGAAATTGTTGAAAGGGAAGCGCTTGCAGCCAGACTTGCCTGTAGTTGCTCAT
CCGGGCTTT--TGCCCGGTGCACTC-
TTCTGCGGGCAGGCCAGCATCAGTCTGGGCGGTTGGATAAAGGCCTCTGTCATGTACCT
CCTCTCGGGGA-GGCCTTATAGG-
GGAGGCGTAATGCAACCAGCCTGGACTGAGGTCCGCGGATGCTG

>Alternaria_maritima_strain_CBS126.60_GU456317.1

CGTGGTC--GCTGGCTATTGCCGTGTAAAGCCCCTT-
CGACGAGTCGAGTTGTTTGGGAATGCAGCTCTAAATGGGAGGTACATTTCTTCTAAAGC
TAAATATTGGCCAGAGACC-
GATAGCGCACAAGTAGAGTGATCGAAAGATGAAAAGCACTTTGGAAAGAGAGTCAAAC
AGCACGTGAAATTGTTGAAAGGGAAGCGCTTGCAGCCAGACTTGCTTACAGTTGCTCAT
CCGGGTTTT--TACCCGGTGCACTC-
TTCTGTAGGCAGGCCAGCATCAGTTTGGGCGGTAGGATAAAGGTCTCTGTCACGTACCT

CCTTTCGGGGA-GGCCTTATAGG-
GGAGACGACATACTACCAGCCTGGACTGAGGTCCGCGGATGCTG

>Embellisia_sp._isolate_SL468T

CGTGGTC--GCTGGCTATTGCCGTGTAAAGCCCCTT-
CGACGAGTCGAGTTGTTTGGGAATGCAGCTCTAAATGGGAGGTACATTTCTTCTAAAGC
TAAATATTGGCCAGAGACC-
GATAGCGCACAAGTAGAGTGATCGAAAGATGAAAAGCACTTTGGAAAGAGAGTCAAAC
AGCACGTGAAATTGTTGAAAGGGAAGCGCTTGCAGCCAGACTTGCTTGCAGTTGCTCAT
CCGGGCTTT--TGCCCGGTGCACTC-
TTCTGTAGGCAGGCCAGCATCAGTTTGGGCGGTAGGATAAAGGTCTCTGTCACGTACCT
CTCTTCGGGGA-GGCCTTATAGG-
GGAGACGACATACTACCAGCCTGGACTGAGGTCCGCGGATGCTG

>Dendryphiella_arenaria_isolate_AFTOL-ID995_DQ470971.1

CGTGGTC--GCTAGCTATTGCCGTGTAAAGCCCCTT-
CGACGAGTCGAGTTGTTTGGGAATGCAGCTCTAAATGGGAGGTAAATTTCTTCTAAAGC
TAAATATTGGCCAGAGACC-
GATAGCGCACAAGTAGAGTGATCGAAAGATGAAAAGCACTTTGGAAAGAGAGTCAAAC
AGCACGTGAAATTGTTGAAAGGGAAGCGCTTGCAGCCAGACTTGCTTGCAGTTGCTCAT
CCGGGCTTT--TGCCCGGTGCACTC-
TTCTGTAGGCAGGCCAGCATCAGTTTGGGCGGTGGGATAAAGGTCTCTGTCACGTACCT
CTCTTCGGGGA-GGCCTTATAGG-
GGAGACGACATACCACCAGCCTAGACTGAGGTCCGCGGATGCTG

>Paradendryphiella_arenaria_isolate_LD40H

CGTGGTC--GCTAGCTATTGCCGTGTAAAGCCCCTT-
CGACGAGTCGAGTTGTTTGGGAATGCAGCTCTAAATGGGAGGTAAATTTCTTCTAAAGC
TAAATATTGGCCAGAGACC-
GATAGCGCACAAGTAGAGTGATCGAAAGATGAAAAGCACTTTGGAAAGAGAGTCAAAC
AGCACGTGAAATTGTTGAAAGGGAAGCGCTTGCAGCCAGACTTGCTTGCAGTTGCTCAT
CCGGGCTTT--TGCCCGGTGCACTC-
TTCTGTAGGCAGGCCAGCATCAGTTTGGGCGGTGGGATAAAGGTCTCTGTCACGTACCT
CTCTTCGGGGA-GGCCTTATAGG-
GGAGACGACATACCACCAGCCTAGACTGAGGTCCGCGGATGCTG

>Arthopyrenia_salicis_strain_CBS368.94_AY538339.1

CGTGGCC--GCCGGTCTTCACCGTGTAAAGCCCCTT-
CGACGAGTCGAGTTGTTTGGGAATGCAGCTCTAAATGGGAGATACATTTCTTCTAAAGC
TAAATATTGGCCAGAGACC-
GATAGCGCACAAGTAGAGTGATCGAAAGATGAAAAGCACTTTGGAAAGAGAGTCAAAA
AGCACGTGAAATTGTTGAAAGGGAAGCGCTTGCAGCCAGACGTGCCCGCAGTTGCTCAC
CCGGGCTCT--CGCCCGGGGCATTC-
TTCTGCGGGCAGGCCAGCATCAGTTCGGGCGGTTGGATAAAGGCCTCCATCACGTATCT

TCCTTCGGGAT-GACCTTATAGG-
GGAGGCGCAACACGACCAGCCTGAACTGAGGACCGCGGATGCTG

>Arthropyrenia_salicis_isolate_AN120R

CGTGGCC--GCCGGTCTTCGCCGTGTAAAGCCCCTT-
CGACGAGTCGAGTTGTTTGGGAATGCAGCTCTAAATGGGAGATACATTTCTTCTAAAGC
TAAATATTGGCCAGAGACC-
GATAGCGCACAAGTAGAGTGATCGAAAGATGAAAAGCACTTTGGAAAGAGAGTCAAAA
AGCACGTGAAATTGTTGAAAGGGAAGCGCTTGCAGCCAGACGTGCCCGCAGTTGCTCAC
CCAGGCTCT--TGCCTGGGGCACTC-
TTCTGCGGGCAGGCCAGCATCAGTTCGGGCGGTTGGATAAAGGTCTCTATCACGTACCA
CCCTTCGGGGT-GTCCTTATAGG-
GGAGACGCAACACGGCCAGCCTGAACTGAGGACCGCGGATGCTG

>Exosporium_stylobatum_strain_CBS160.30_JQ044447.1

TGTGGCC--GCCAGTCTTCGCCGTGTAAAGCCCCTT-
CGACGAGTCGAGTTGTTTGGGAATGCAGCTCTAAATGGGAGGTAAATTTCTTCTAAAGC
TAAATATTGGCCAGAGACC-
GATAGCGCACAAGTAGAGTGATCGAAAGATGAAAAGCACTTTGGAAAGAGAGTCAAAA
AGCACGTGAAATTGTTGAAAGGGAAGCGCTTGCAGCCAGACTTGCTTGCAGTTGTTCAG
CCGGGCTCT--TGCCCGGTCTACTC-
TTCTGCTCGCAGGCCAGCATCAGTCCGGGCGGTTGGATAAAGGCCTATTAAACGTACCT
CCCCTCGGGGA-GGACTTATAGG-
GTAGGCGACATGCAACCAGCCCGGATTGAGGTCCGCGGATGCTG

>Exosporium_stylobatum_isolate_AN122R

TGTGGCC--GCCAGTCTTCGCCGTGTAAAGCCCCTT-
CGACGAGTCGAGTTGTTTGGGAATGCAGCTCTAAATGGGAGGTAAATTTCTTCTAAAGC
TAAATATTGGCCAGAGACC-
GATAGCGCACAAGTAGAGTGATCGAAAGATGAAAAGCACTTTGGAAAGAGAGTCAAAA
AGCACGTGAAATTGTTGAAAGGGAAGCGCTTGCAGCCAGACTTGCTTGCAGTTGTTCAG
CCGGGCTCT--TGCCCGGTCTACTC-
TTCTGCTCGCAGGCCAGCATCAGTCCGGGCGGTTGGATAAAGGCCTATTAAACGTACCT
CCCCTCGGGGA-GGACTTATAGG-
GTAGGCGACATGCAACCAGCCCGGATTGAGGTCCGCGGATGCTG

>Pyrenochaetopsis_pratorum_strain_CBS445.81_GU238136.1

CGTGGTC--GCTGGCTAACGCCGTGTAAAGCCCCTT-
CGACGAGTCGAGTTGTTTGGGAATGCAGCTCTAAATGGGAGGTAAATTTCTTCTAAAGC
TAAATACTGGCCAGAGACC-
GATAGCGCACAAGTAGAGTGATCGAAAGATGAAAAGCACTTTGGAAAGAGAGTCAAAC
AGCACGTGAAATTGTTGAAAGGGAAGCGCTTGCAGCCAGACTTGCCTGTAGTTGCTCAT
CCGGGCTTT--TGCCCGGTGCATTC-
TTCTGTAGGCAGGCCAGCATCAGTTTGGGCGGTTGGATAAAGACCTCTGTCACGTACCTT

CTCTCGGGAA-GGCCTTATAGG-
GGAGGTGTCATGCAACCAGCCTGGACTGAGGTCCGCGGATGCTG

>Pyrenochaetopsis_pratorum_isolate_LD326H

CGTGGTC--GCTGGCTAACGCCGTGTAAAGCCCCTT-
CGACGAGTCGAGTTGTTTGGGAATGCAGCTCTAAATGGGAGGTAAATTTCTTCTAAAGC
TAAATACTGGCCAGAGACC-
GATAGCGCACAAGTAGAGTGATCGAAAGATGAAAAGCACTTTGGAAAGAGAGTCAAAC
AGCACGTGAAATTGTTGAAAGGGAAGCGCTTGCAGCCAGACTTGCCTGTAGTTGCTCAT
CCGGGCTTT--TGCCCGGTGCATTC-
TTCTGTAGGCAGGCCAGCATCAGTTTGGGCGGTTGGATAAAGACCTCTGTCACGTACCTT
CTCTCGGGAA-GGCCTTATAGG-
GGAGGTGTCATGCAACCAGCCTGGACTGAGGTCCGCGGATGCTG

>Boeremia_strasseri_strain_CBS261.92_GU237957.1

CGTGGTC--GCTAGCCTTTACCGTGTAAAGCCCCTT-
CGACGAGTCGAGTTGTTTGGGAATGCAGCTCTAAATGGGAGGTAAATTTCTTCTAAAGC
TAAATACTGGCCAGAGACC-
GATAGCGCACAAGTAGAGTGATCGAAAGATGAAAAGCACTTTGGAAAGAGAGTTAAAA
AGCACGTGAAATTGTTGAAAGGGAAGCGCTTGCAGCCAGACTTGCCTGTAGTTGCTCAT
CCGGGTTTT--TACCCGGTGCACTC-
TTCTATGGGCAGGCCAGCATCAGTTTGGGCGGTTGGATAAAGGTCTCTGTCACGTACCTC
CTCTCGGGGA-GAACTTATAGG-
GGAGACGTAATGCAACCAGCCTGGACTGAGGTCCGCGGATGCTG

>Phoma_exigua_isolate_SL333T

CGTGGTC--
GCTAGCCTTTACCGTGTAAAGCCCCTTTCGACGAGTCGAGTTGTTTGGGAATGCAGCTCT
AAATGGGAGGTAAATTTCTTCTAAAGCTAAATACTGGCCAGAGACC-
GATAGCGCACAAGTAGAGTGATCGAAAGATGAAAAGCACTTTGGAAAGAGAGTTAAAA
AGCACGTGAAATTGTTGAAAGGGAAGCGCTTGCAGCCAGACTTGCCTGTAGTTGCTCAT
CCGGGTTTT--TACCCGGTGCACTC-
TTCTATAGGCAGGCCAGCATCAGTTTGGGCGGTTGGATAAAGGTCTCTGTCACGTACCTC
CTCTCGGGGA-GAACTTATAGG-
GGAGACGTAATGCAACCAGCCTGGACTGAGGTCCGCGGATGCTG

>Epicoccum_nigrum_strain_CBS173.73_GU237975.1

CGTGGTC--GCTAGCCTTTACCGTGTAAAGCCCCTT-
CGACGAGTCGAGTTGTTTGGGAATGCAGCTCTAAATGGGAGGTAAATTTCTTCTAAAGC
TAAATACTGGCCAGAGACC-
GATAGCGCACAAGTAGAGTGATCGAAAGATGAAAAGCACTTTGGAAAGAGAGTTAAAA
AGCACGTGAAATTGTTGAAAGGGAAGCGCTTGCAGCCAGACTTGCCTGTAGTTGCTCAT
CCGGGTTTT--TACCCGGTGCACTC-
TTCTACGGGCAGGCCAGCATCAGTTTGGGCGGTTGGATAAAGGTCTCTGTCACGTACCTC

CTCTCGGGGA-GATCTTATAGG-
GGAGACGACATGCAACCAGCCTGGACTGAGGTCCGCGGATGCTG

>Epiccocum_nigrum_isolate_SL332T

CGTGGTC--GCTAGCCTTTACCGTGTAAAGCCCCTT-
CGACGAGTCGAGTTGTTTGGGAATGCAGCTCTAAATGGGAGGTAAATTTCTTCTAAAGC
TAAATACTGGCCAGAGACC-
GATAGCGCACAAGTAGAGTGATCGAAAGATGAAAAGCACTTTGGAAAGAGAGTTAAAA
AGCACGTGAAATTGTTGAAAGGGAAGCGCTTGCAGCCAGACTTGCCTGTAGTTGCTCAT
CCGGGTTTT--TACCCGGTGCACTC-
TTCTACGGGCAGGCCAGCATCAGTTTGGGCGGTTGGATAAAGGTCTCTGTCATGTACCTC
CTCTCGGGGA-GATCTTATAGG-
GGAGACGACATGCAACCAGCCTGGACTGAGGTCCGCGGATGCTG

Alignment 2. Tubuline

>gi|400034452|gb|JN853971.1|_Aspergillus_versicolor_isolate_NRRL_227_beta-
tubulin_gene_partial_cds

GCGTGACAGGGTAACCAAATTGGTGCTGCTTTCTGGTGCGTCGAAAATTTCATCCATTTC
AGATGGTATTTCCTTTCGTG-
CTTTTTGCTAACGACTCTATAGGCAGACCATCTCCGGTGAGCACGGCCTCGATGGCTCCG
GTGTGTGAGTACAACCCGTCCAGGACTCGATCAAAACACGAGACAGAACACATCCCCTG
ATAGAATGC-
AGTTACAATGGTACCTCCGACCTCCAGCTCGAGCGTATGAACGTCTACTTCAACGAGG---
----
CCAGCGGCAACAAGTACGTTCCTCGTGCCGTCCTCGTCGATCTCGAGCCCGGTACCATG
GACGCTGTCCGTGCCGGTCCCTTCGGTC-
AGCTTTTCCGTCCCGACAACTTCGTCTTTGGCCAGTCCGGTGCTGGTAACAACTGGGCCA
AGGGTCG-

>Aspergillus versicolor isolate LD294F

CAAAACGGTGCTGCTTTCTGGTGCGTCGAAAATTTCATCCATTTCAGATGGTATCTCCTT
TCGTG-
CTTTTTGCTAACGACTCTATAGGCAGACCATCTCCGGTGAGCACGGCCTCGATGGCTCCG
GTGTGTGAGTACAACCCGTCCAGGACTCGATCAAAACACGAGACAGAACACATCCCCTG
ATATAATGC-
AGTTACAATGGTACCTCCGACCTCCAGCTCGAGCGTATGAACGTCTACTTCAACGAGG---
----
CCAGCGGCAACAAGTACGTTCCTCGTGCCGTCCTCGTCGATCTCGAGCCCGGTACCATG
GACGCTGTCCGTGCCGGTCCCTTCGGTC-AGCTTTTCCGTCCCGACAACTTCGTCTT-
GGCCAGTCCG----------------------------

>Penicillium brevicompactum AN312R

TACAACGGTACCTCCGACCTCCAGCTGGAGCGTATGAACGTCTACTTCAACCATGTGAG
TACAATGCCTTGGAAAAATCTTGTTGTGTGTTTGCTCACC---
CGGATTTTTTGACCTCCCAGGCTATTGGTGACAAGTATGTTCCCCGTGCCGTCCTCGTCT
ACTTGGAGCCCGGTACC--

>gi|190683756|gb|EU587353.1|_Penicillium_brevicompactum_strain_DAOM_215332_beta-
tubulin_gene_partial_sequence

-----------------------TGGTATGTACCGCATCACGGTCT-
TTTTTTCCTCCCGCAATGGCTGGGTATCAATTGACATCTTGCTAACTGACATAAAGGCAA
ACTATCTCCGGCGAGCACGGTCTCGATGGCGATGGACAGTAAGTG---------
GAGCGTACTGGGA---TCCTATGTGGATTGGT-T--
CTGATATATTGTTAGGTACAACGGTACCTCCGACCTCCAGCTGGAGCGTATGAACGTCTA
CTTCAACCATGTGAGTACAATGCCTTGGAAAAATCTTGTTGTGTGTTTGCTCACC---
CGGATTTTTTGACCTCCCAGGCTAGTGGTGACAAGTATGTTCCCCGTGCCGTCCTCGTCG
ACTTGGAGCCCGGTACCATGGACGCTGTCCGCTCCGGTCCCTTCGGCA-
AGCTTTTCCGCCCCGACAACTTCGTCTTCGGTCAGTCCGGTGCTGGTAACAACTG-----------
--

>Penicillium echinatum isolate LD69H

------CCTGGTAACCAAATCGGTGCTGCTTTTTGGTACGTGCAGA-
CAGATATCGAAACTTTTTTCGCGTCATTGGTTCACAATTTACTGACTGGATTACAGGCAA
ACCATCTCCGGTGAGCACGGTCTCGATGGCGATGGACAGTAAGTT---------
CAATGTGGAATTT---CTTGTGGTGGATTGGG-
CAGCTGATATCTGGTTAGGTACAACGGTACCTCCGACCTCCAGCTCGAGCGCATGAACG
TCTACTTCAACCATGTGAGTACAATATGTTGGAATTGGCTGCTTAAGCATTATCTGACT---
TCTATGTTTTGACCCCTCAGGCCAACGGTGACAAGTACGTTCCCCGTGCCGTTCTCGTCG
ACTTGGAGCCCGGTACCATGGACGCTGTCCGCTCCGGTCCTTTCGGCA-AGCTTT-
CCGCCCCGACAACT-CGTCTCGG--------------------------------------

>gi|402514028|gb|JX140952.1|_Penicillium_echinatum_strain_NRRL917_beta-
tubulin_gene_partial_cds

---------------------GGTGCTGCTTTCTGGTACGTGCAGA-CAGATATCGAAACTTTTT-
CGCGTCATTGGTTCACAATTTACTGACTGGATTACAGGCAAACCATCTCCGGTGAGCAC
GGTCTCGATGGCGATGGACAGTAAGTT---------CAACATGGAATTT---
CTTGTGGTGGATTGGG-
CAGCTGATATCTTGTTAGGTACAACGGTACCTCCGACCTCCAGCTCGAGCGCATGAACG
TCTACTTCAACCATGTGAGTACAATATGTTGGAATTGGCTGCTTAAGCATTATCTGACT---
TCTATGTTTTGACCCCTCAGGCCCACGGTGACAAGTACGTTCCCCGTGCCGTTCTCGTCG
ACTTGGAGCCCGGTACCATGGACGCTGTCCGCTCCGGTCCTTTCGGCA-
AGCTTTTCCGCCCCGACAACTTCGTCTTCGGTCAGTCCGGTGCTGGTAACAACTGGGCCA
AGG-----

>Penicillium polonicum isolate LD330H

AAACGGTGCTGCTTTCTGGTAAGTGCCGAGCTTTTTTTTCTTCTTCGCGTTGGGTATCAAT
TGACAGGTTACTAACTCGATTACAGGCAAACCATCTCTGGCGAGCACGGTCTCGATGGC
GATGGACAGTAAGTT---------TTAATGGTGATGTGGGTTTCCGGTAGATCACA-
CGTCTGATATCTTGCTAGGTACAATGGTACCTCCGACCTCCAGCTCGAGCGTATGAACGT
CTACTTCAACCATGTGAGTCCAATCACTGGAAACCGAATAATCGTGCATCATCTGATCA
GATGTTTTTCTTTGATATCTAGGCCAGCGGTGACAAGTACGTTCCCCGTGCCGTTCTCGT
CGATTTGGAGCCTGGTACCATGGACGCTGTCCGCTCCGGTCCTTTCGGCA-AGCTTT-
CCGCCCCGACAACT-CGTCTCG--------------------------------------

>gi|53830446|gb|AY674306.1|_Penicillium_polonicum_strain_CBS_101479_beta_tubulin-like_gene_partial_sequence

----
AATCGGTGCTGCTTTCTGGTAAGTGCCGAGCTTTTTTTTCTTCTTCGCGTTGGGTATCAAT
TGACAGGTTACTAACTCGATTACAGGCAAACCATCTCTGGCGAGCACGGTCTCGATGGC
GATGGACAGTAAGTT---------TTAATGGTGATGTGGGTTTCCGGTAGATCACA-
CGTCTGATATCTTGCTAGGTACAATGGTACCTCCGACCTCCAGCTCGAGCGTATGAACGT
CTACTTCAACCATGTGAGTCCAATCACTGGAAACCGAATAATCGTGCATCATCTGATCA
GATGTTTTTCTTTGATATCTAGGCCAGCGGTGACAAGTACGTTCCCCGTGCCGTTCTCGT
TGATTTGGAGCCTGGTACCATGGACGCTGTCCGCTCCGGTCCTTTCGGCA-
AGCTTTTCCGCCCCGACAACTTCGTCTTCGGTCAGTCCGGTGCTGGTAACAACTGGGCCA
AGGGTC--

>Talaromyces atricola LD147H

-----------------AAACGGTGCTGCTTTCTGGTGAGTTGGGAGATAAA-
CAATGGCATAAAAAAAAACCTTG-----------GCTGACC-
TTGTACAGGCAAACCATCTCTGGCGAGCACGGCCTCGATGGCTCCGGAGTGTGAGTG-----
-ATACGTGATT--GTCAAAAAATTTCA-AGAACCAA--CCCCGATGGATTCC-
AGTTACAATGGTACCTCCGCCCTCCAGTTGGAGCGTATGAACGTCTACTTCAACGAGGT
GCGTGGAATCCCAAGTCTCGACATTCGGATATATA-----------------
CTTATAACGTATAGGCTAGCAACAACAAGTATGTCCCTCGTGCCGTCCTCGTCGATTTGG
AGCCTGGCACCATGGACGCGGTGCGTGCCGGTCCCTTCGGCCGAGCCTCTCCGCCCCGA
CAACT-CGTCTCG--------------------------------------

>gi|543888516|gb|KF196849.1|_Talaromyces_atricola_isolate_NRRL_1052_beta-tubulin_gene_partial_cds

ATTCAATAGGGTAACCAAATTGGTGCCGCCTTCTGGTGAGTTGGGAGATAAA-
CAATGGCATAAAAAAAA-CCTTG-----------GCTGACC-
TTGTACAGGCAAACCATCTCTGGCGAGCACGGCCTCGATGGCTCCGGAGTGTGAGTG-----
-ATACATGATT--GTTAAAAAATTTCA-AGAACCAA--CCCTGATGGATTCC-
AGTTACAATGGTACCTCCGACCTCCAGTTGGAGCGTATGAACGTCTACTTCAACGAGGT
GCGTGGAATCCCAAGTCTCGACATTCGGATATATA-----------------
CTCATAACGTATAGGCTAGCAACAACAAGTATGTCCCTCGTGCCGTCCTCGTCGATTTGG
AGCCTGGCACCATGGACGCGGTCCGTGCCGGTCCCTTCGGCC-

AGCTTTTCCGCCCCGACAACTTCGTCTTCGGCCAGTCTGGTGCTGGTAACAACTGGGCCA
AGGGTCAG

Alignment 3. ITS

>gi|158535901|gb|EF652440.1|_Aspergillus_versicolor_NRRL_227_18S_rib_RNA_gene_partial_seq_internal_transcribed_spacer_1_5.8S_ribosomal_RNA_gene_and_internal_transcribed_spacer_2_complete_sequence_e

---CCCTACCTGATCCGAGGTCAAC-
CTGAAGAAAATGGTTGGAGACGTCGGCTGGCG-CCC--GGCCG---
GCCCTAATCGAGCGGGTGACAAAGCCCCA-TACGCTCGAG-GACCGGACACGGTGCCGC--
CGCTGCCTTTCGG---GCCCGTCCCCCG--G---GGGGGACGACGA--------CCCAACACAC-------
---------AAGCCGGG---
CTTGATGGGCAGCAATGACGCTCGGACAGGCATGCCCCCCGGAATGCCAGGGGGCGCA
ATGTGCGTTCAAAGACTCGATGATTCACTGAATTCTGCAATTCACATTACTTATCGCAGT
TCGCTGCGTTCTTCATCGATGCCGGAACCAAGAGATCCATTGTTGAAAGTTTTG-
ACTGATTTTATAT----TCAGA-----------------CTCAGACTGCAT------CACTCT-------------CAGG-
---CA--TGAAG--TTCAGTAGTC-----C--------CCGGCGGCT----------CGCCCCCGAGAGGGCTC-
----------------CCCGCCGAAGCAACAGT--GTTA-
GGTAGTCACGGGTGGGAGGTTGGGCGCCCGGAGGC----
AGCCCGCACTCGGTAATGATCCTT--

>Aspergillus versicolor isolate LD147H

AGAAAATGGTTGGAGACGTCGGCTGGCG-CCC--GGCCG---
GCCCTAATCGAGCGGGTGACAAAGCCCCA-TACGCTCGAG-GACCGGACACGGTGCCGC--
CGCTGCCTTTCGG---GCCCGTCCCCCG--G---GGGGGACGACGA--------CCCAACACAC-------
---------AAGCCGGG---
CTTGATGGGCAGCAATGACGCTCGGACAGGCATGCCCCCCGGAATGCCAGGGGGCGCA
ATGTGCGTTCAAAGACTCGATGATTCACTGAATTCTGCAATTCACATTACTTATCGCAGT
TCGCTGCGTTCTTCATCGATGCCGGAACCAAGAGATCCATTGTTGAAAGTTTTG-
ACTGATTTTATAT----TCAGA-----------------CTCAGACTGCAT------CACTCT-------------CAGG-
---CA--TGAAG--TTCAGTAGTC-----C--------CCGGCGGCT----------CGCCCCCGAGAGGGCTC-
----------------CCCGCCGAAGCAACAGT--GTTA-
GGTAGTCACGGGTGGGAGGTTGGGCGCCCGGAGGC----
AGCCCGCACTCGGTAATGATCCTTCCGCAGGTTCACCTACGGA------------------------------------
----

>gi|34809381|gb|AY373901.1|_Penicillium_canescens_strain_FRR_910_18S_ribosomal_RNA_gene_partial_sequence_internal_transcribed_spacer_1_5.8S_ribosomal_RNA_gene_and_internal_transcribed_spacer_2_complete

---CCCTACCTGATCCGAGGTCAA-
CCTGGAAAAAAGTTTTGGTTGATCGGCAAGCG-CC---GGCCG---GGCC-
TACAGAGCGGGTGACAAAGCCCCA-TACGCTCGAG-GACCGGACGCGGTGCCGC--

CGCTGCCTTTCGG---GCCCGTCCCCCCGGGA-AGGGGGACGAGA---------CCCAACACAC---
-------------AAGCCGGG---CTTGA-
GGGCAGCAATGACGCTCGGACAGGCATGCCCCCCGGAATACCAGGGGGCGCAATGTGC
GTTCAAAGACTCGATGATTCACTGAATTCTGCAATTCACATTACGTATCGCATTTCGCTG
CGTTCTTCATCGATGCCGGAACCAAGAGATCCGTTGTTGAAAGTTTTA-AATAATTT-
ATAT----TTAGA-----------------CTCAGACTGCAA------TTTTCATA-----------CAGAGTT-CA--
AGGTGTCTTCGGCGGGCGCGGGC--------CCGGGGGCA--------
GATGCCCCCCGGCGGCCGTGA---------GGCGGGCCCGCCGAAGCAACAAG--GTAC-
AATAAACACGGGTGGGAGGTTGAAT--TCAGAGA------
ATTCTCGCTCGGTAATGATCCTTCCGCAGGTTCACCTACGGA--------------------------------------
----

>Penicillium echinatum isolate LD69H

--TGCTCCGAGGTCAA-
CCTGGAAAAAAGTTTTGGTTGATCGGCAAGCG-CC---GGCCG---GGCC-
TACAGAGCGGGTGACAAAGCCCCA-TACGCTCGAG-GACCGGACGCGGTGCCGC--
CGCTGCCTTTCGG---GCCCGTCCCCCCGGGA-AGGGGGACGAGA---------CCCAACACAC---
-------------AAGCCGGG---CTTGA-
GGGCAGCAATGACGCTCGGACAGGCATGCCCCCCGGAATACCAGGGGGCGCAATGTGC
GTTCAAAGACTCGATGATTCACTGAATTCTGCAATTCACATTACGTATCGCATTTCGCTG
CGTTCTTCATCGATGCCGGAACCAAGAGATCCGTTGTTGAAAGTTTTA-AATAATTT-
ATAT----TTAGA-----------------CTCAGACTGCAA------TTTTCATA-----------CAGAGTT-CA--
AGGTGTCTTCGGCGGGCGCGGGC--------CCGGGGGCA--------
GATGCCCCCCGGCGGCCGTGA---------GGCGGGCCCGCCGAAGCAACAAG--GTAC-
AATAAACACGGGTGGGAGGTTGAAT--TCAGAGA------
ATTCTCGCTCGGTAATGATCCTTCCGCAGGTTCACCTACGGAAACCTTGTTACGATTTTTT
ACTTCCCA--

>gi|3925740|emb|AJ005492.1|_Penicillium_polonicum_(IBT_11388)_ribosomal_internal_transcribed_spacers_and_the_5.8S_ribosomal_RNA_gene_(ITS1-5.8S-ITS2)

---CCCTACCTGATCCGAGGTCAA-
CCTGGATAAAAATTTGGGTTGATCGGCAAGCG-CC---GGCCG---GGCC-
TACAGAGCGGGTGACAAAGCCCCA-TACGCTCGAG-GACCGGACGCGGTGCCGC--
CGCTGCCTTTCGG---GCCCGTCCCCCGGAAT-CGGAGGACGGGG---------CCCAACACAC----
------------AAGCCGGG---CTTGA-
GGGCAGCAATGACGCTCGGACAGGCATGCCCCCCGGAATACCAGGGGGCGCAATGTGC
GTTCAAAGACTCGATGATTCACTGAATT-
TGCAATTCACATTACGTATCGCATTTCGCTGCGTTCTTCATCGATGCCGGAACCAAGAGA
TCCGTTGTTGAAAGTTTTA-AATAATTT-ATAT----TTTCA-----------------CTCAGACTTCAA---
---TCTTCAGA-----------CAGAGTTCGG--GGGTGTCTTCGGCGGGCGCGGGC--------
CCGGGGGCG--------TGAGCCCCCCGGCGGCCAGTA-------
AAGGCGGGCCCGCCGAAGCAACAAG--GTAA-AATAAACACGGGTGGGAGGTTGGAC--
CCAAAGG------
GCCCTCACTCGGTAATGATCCTTCCGCAGGTTCACCTACGGAAACCTTGTTACGACTTTT
ACTTCC--

>Penicillium polonicum isolate LD330H

---CTGATCCGAGGTCA--
CCTGGATAAAAATTTGGGTTGATCGGCAAGCG-CC---GGCCG---GGCC-
TACAGAGCGGGTGACAAAGCCCCA-TACGCTCGAG-GACCGGACGCGGTGCCGC--
CGCTGCCTTTCGG---GCCCGTCCCCCGGAAT-CGGAGGACGGGG---------CCCAACACAC----
------------AAGCCGGG---CTTGA-
GGGCAGCAATGACGCTCGGACAGGCATGCCCCCCGGAATACCAGGGGGCGCAATGTGC
GTTCAAAGACTCGATGATTCACTGAATT-
TGCAATTCACATTACGTATCGCATTTCGCTGCGTTCTTCATCGATGCCGGAACCAAGAGA
TCCGTTGTTGAAAGTTTTA-AATAATTT-ATAT----TTTCA----------------CTCAGACTTCAA---
---TCTTCAGA-----------CAGAGTTCGG--GGGTGTCTTCGGCGGGCGCGGGC--------
CCGGGGGCG--------TGAGCCCCCCGGCGGCCAGTA-------
AAGGCGGGCCCGCCGAAGCAACAAG--GTAA-AATAAACACGGGTGGGAGGTTGGAC--
CCAAAGG------
GCCCTCACTCGGTAATGATCCTTCCGCAGGTTCACCTACGGAAACCTTGTTACGATTTTT
ACTTCCA--

>Penicillium_brevicompactum_strain_NRRL_32600_DQ123640.1

---CCCTACCTGATCCGAGGTCAA-
CCTGGAAAAAAGTTTGGTTG-ATCGGCAGGCG-CC---GGCCA---GTCC-
TACAAAGCGGGTGACAAAGCCCCA-TACGCTTGAG-GACCGGACGCGGTGCCGC--
CGCTGCCTTTCGG---GCCCGTCCCCCGGAA---GGAGGACGGAG---------CCCAACACAC------
----------AAGCCGTG---CTTGA-
GGGCAGCAATGACGCTCGGACAGGCATGCCCTCCGGAATACCAGAGGGCGCAATGTGC
GTTCAAAGACTCGATGATTCACTGAATTCTGCAATTCACATTACGTATCGCATTTCGCTG
CGTTCTTCATCGATGCCGGAACCAAGAGATCCGTTGTTGAAAGTTTTA-AATAATTT-
ATAT----TTAAT-----------------CTCAGACTACAA------TCTTCAGA-----------CAGAGTTCTA--
AGGTGTCTTCGGCGAGCGCGGAC--------CCGGGGACA--------
AACGTCCCCCGGCAGCCAGAA---------GGCAGGCTCGCCGAAGCAACAAG--GTAA-
AATAAACACGGGTGGGAGGTTGGAC--CCAGAGG-----G-
CCCTCACTCGGTAATGATCCTT---

>Penicillium brevicompactum isolate AN312R

--AAAGTTTGGTTG-
ATCGGCAGGCG-CC---GGCCA---GTCC-TACCAAGCGGGTGACAAAGCCCCA-
TACGCTTGAG-GACCGGACGCGGTGCCGC--CGCTGCCTTTCGG---GCCCGTCCCCCGGAA-
--GGAGGACGGAG---------CCCAACACAC----------------AAGCCGTG---CTTGA-
GGGCAGCAATGACGCTCGGACAGGCATGCCCTCCGGAATACCAGAGGGCGCAATGTGC
GTTCAAAGACTCGATGATTCACTGAATTCTGCAATTCACATTACGTATCGCATTTCGCTG
CGTTCTTCATCGATGCCGGAACCAATAGATCCGTTGTTGAAAGTTTTA-AATAATTT-
ATAT----TTAAT-----------------CTCAGACTACAA------TCTTCAGA-----------CAGAGTTCTA--
AGGTGTCTTCGGCGAGCGCGGAC--------CCGGGGACA--------
GATGTCCCCCGGCAGCCAAAA---------GGCAGGCTCGCCGAAGCAACAAG--GTAA-

AATAAACACGGGTGGGAGGTTGGAC--CCAGAGG-----G-
CCCTCACTCGGTAATGATCCTTCCGCAA--

>Penicillium_rugulosum_D4_GU566230.1

--GGTAGACGGGC-
CCCGAAGGGTCGCCTGGAGG-AA-GACCAGCG-CC---GACGA---
GTCCGTCCCGAGCGGGTGACAAAGCCCCA-TACGCTCGAG-GACCCGACGCGGCGCCGC--
CACTGCCTTTGGG---GCGTGTCCCC----G---GGGGGACAACA---------CCCAACACCC---------
------A-GCCGGG---CTGGA-
GGGCAGAAATGACGCTCGGACAGGCATGCCCCCCGGAATGCCAGGGGGCGCAATGTGC
GTTCAAAGATTCGATGATTCACGGAATTCTGCAATTCACATTACTTATCGCATTTCGCTG
CGTTCTTCATCGATGCCGGAACCAAGAGATCCGTTGTTGAAAGTTTTA-A-TGATTT-
AATC----ATAGA-----------------CTCAGACT-CAC------TATTCAGA-----------CAGGGTTCCA--
GGGCG-CTTCGGCGGGCGCGGAC--------CCGGGGGCA--------
GAAGCCCCCCGGCGACCGGGGCCAGGCCCCAGTGGGCCCGCCGAGGCAACGCG--
GTCATGGTAAACACGGGTGGGAGGTTGGGC--TCGTTGG-----
AACCCGCACTCGGTAATGATCCTTCCGCAGGTTCACCTACGGA-----------------------------------
----

>gi|725095880|gb|KF984859.1|_Talaromyces_atricola_strain_CBS_255.31_18S_ribosomal_RNA_gene_partial_sequence_internal_transcribed_spacer_1_5.8S_ribosomal_RNA_gene_and_internal_transcribed_spacer_2_compe

TTTCCTCCGCTTATTGATATGCTTAAGTTCAGCGGGTAACCCTACCTGATCCGAGGTCAA
CCGAAGGAAGACGAGC-CCCGAAGGGTCGCCTGGAGG-AA-GACCAGCG-CC---GACGA---
GTCCGTCCCGAGCGGGTGACAAAGCCCCA-TACGCTCGAG-GACCCGACGCGGCGCCGC--
CACTGCCTTTGGG---GCGTGTCCCC----G---GGGGGACAGCG---------CCCAACACCC---------
------A-GCCGGG---CTGGA-
GGGCAGAAATGACGCTCGGACAGGCATGCCCCCCGGAATGCCAGGGGGCGCAATGTGC
GTTCAAAGATTCGATGATTCACGGAATTCTGCAATTCACATTACTTATCGCATTTCGCTG
CGTTCTTCATCGATGCCGGAACCAAGAGATCCGTTGTTGAAAGTTTTA-A-TGATTC-
AATC----ATTCA-----------------CTCAGACT-CAC------TATTCAGA-----------CAGGGTTCCA--
GGGCG-CTTCGGCGGGCGCGGAC--------CCGGGGGCAA-------
GGAGCCCCCCGGCGACCGGGGCCAGGCCCCAGTGGGCCCGCCGAGGCAACGCG--
GTCATGGTAAACACGGGTGGGAGGTTGGGC--TCGTTGG-----
AACCCGCACTCGGTAATGATCCTT--

>Talaromyces atricola AN312R

--GGTAGACGGGC-
CCCGAAGGGTCGCCTGGAGG-AA-GACCAGCG-CC---GACGA---
GTCCGTCCCGAGCGGGTGACAAAGCCCCA-TACGCTCGAG-GACCCGACGCGGCGCCGC--
CACTGCCTTTGGG---GCGTGTCCCC----G---GGGGGACAGCA---------CCCAACACCC---------
------A-GCCGGG---CTGGA-
GGGCAGAAATGACGCTCGGACAGGCATGCCCCCCGGAATGCCAGGGGGCGCAATGTGC
GTTCAAAGATTCGATGATTCACGGAATTCTGCAATTCACATTACTTATCGCATTTCGCTG

CGTTCTTCATCGATGCCGGAACCAAGAGATCCGTTGTTGAAAGTTTTA-A-TGATTT-AATC----ATCGA----------------CTCAGACT-CAC------TATTCAGA-----------CAGGGTTCCA--GGGCG-CTTCGGCGGGCGCGGAC--------CCGGGGGCA--------GAAGCCCCCCGGCGACCGGGGCCAGGCCCCAGTGGGCCCGCCGAGGCAACGCG--GTCATGGTAAACACGGGTGGGAGGTTGGGC--TCGTTGG-----AACCCGCACTCGGTAATGATCCTTCCGCAGGTTCACCTACGGA---

>gi|385889185|gb|JQ796751.1|_Cadophora_luteo-olivacea_18S_ribosomal_RNA_gene_partial_sequence_internal_transcribed_spacer_1_5.8S_ribosomal_RNA_gene_and_internal_transcribed_spacer_2_complete_sequence_e

--TCCCTACCTGATCCGAGGTCAA-CCTGTAAAAATTGGG-GGTTGCTGGCAAGTAG-AC----CTAC---CGGACCCAGACGCGAG---GAGTATTAC--TACGC-GTAG-AGCCGA--CAGGCACCGC--CACTGATTTTAGG---GGCCGCGGAACC-----------GCG-AA--------CCCCAATACCA----------------AGCGAGA--GCTTGAGTGGTTATAATGACGCTCGAACAGGCATGCCCCCCGGAATACCAGAGGGCGCAATGTGCGTTCAAAGATTCGATGATTCACTGAATTCTGCAATTCACATTACTTATCGCATTTCGCTGCGTTCTTCATCGATGCCAGAACCAAGAGATCCGTTGTTGAAAGTTTTA-ACTATTATATAG------TA-------------------CTCAGACATCAC-------TAAAAAC-----------AAGAGTTGTG-----GTCCTCTGGCGGGCACTCA------------------------ACAGCCGAAG--CCGC--TGGCGCGAG-GCGG-----CCCGCCAAAGCAACAAA--GGTAGTTTATTCAAGGGTGGAGTTCA--GGACCGACCTTC-TCCGAGAGGGTCGACGGCTCTAAACCCTACCGAAGTAGGGTAGCCCCGCCACGAAGCAAGCTTCTGTGGGGCGCTGCCTGTCCTTTGCTC--

>Cadophora luteo-olivacea SL408T

---ACCTGATCCGAGGTCA--CCTGTAAAAATTGGG-GGTTGCTGGCAAGTAG-AC----CTAC---CGGACCCAGACGCGAG---GAGTATTAC--TACGC-GTAG-AGCCGA--CAGGCACCGC--CACTGATTTTAGG---GGCCGCGGAACC-----------GCG-AA--------CCCCAATACCA----------------AGCGAGA--GCTTGAGTGGTTATAATGACGCTCGAACAGGCATGCCCCCCGGAATACCAGAGGGCGCAATGTGCGTTCAAAGATTCGATGATTCACTGAATTCTGCAATTCACATTACTTATCGCATTTCGCTGCGTTCTTCATCGATGCCAGAACCAAGAGATCCGTTGTTGAAAGTTTTA-ACTATTATATAG------TA-------------------CTCAGACATCAC-------TAAAAAC-----------AAGAGTTGTG-----GTCCTCTGGCGGGCACTCA------------------------ACAGCCGAAG--CCGC--TGGCGCGAG-GCGG-----CCCGCCAAAGCAACAAA--GGTAGTTTATTCAAGGGTGGAGTTCA--GGACCGACCTTC-TTCGAGAGGGTCGACGGCTCTAAACCCTACCGAAGTAGGGTAGCCCCGCCACGAAGCAAGCTTCTGTGGGGCGCTGCCTGTCC--

>gi|309384370|gb|HM148035.1|_Cladosporium_cladosporioides_strain_CPC_13669_18S_ribosomal_RNA_gene_partial_sequence_internal_transcribed_spacer_1_5.8S_ribosomal_RNA_gene_and_internal_transcribed_spacere

--CCCTACCTGATCCGAGGTCAA-
CCTTAGAAATGGGGTTGTTTTACGGCGTAGCC-T-----CCCG---AACACCCTTTAGCGAA--
TAGTTTCCAC--AACGCTTAGGGGACAGAA---GACCCAGC--
CGGTCGATTTGAGGCACGCGGCGGACC---------GCGTTG------------CCCAATACCA------------
-----AGCGAGG---CTTGAGTG-
GTGAAATGACGCTCGAACAGGCATGCCCCCCGGAATACCAGGGGGCGCAATGTGCGTTC
AAAGATTCGATGATTCACTGAATTCTGCAATTCACATTACTTATCGCATTTCGCTGCGTT
CTTCATCGATGCCAGAACCAAGAGATCCGTTGTTAAAAGTTTTA-ATTTATTAATTAA----
GTTTA-----------------CTCAGACTGCAA-------AGTTACG----------CAAGAGTTTGA--
AGTGTCCACCCGGAGCCCCC---GCCCGAAGGCAGGG-
TCGCCCCGGAGGCAACA-------
GAGTCGGACAACAAAGGGTTATGAACATCCCGGTGGTTAGACCGGGGTCACTTGTAATG
ATCCCTCCGCAGGTTCACCTACGGAGACCTTGTTACGACTTTTACTTCCTCTAAATGACC
GGGTTTGACCAACTTTCCGGCCCTGGGTGGTCGTTGCCGACCTCCCTGGGCCAGTCCGAA
GGCCTCACCGAGCCAT----------------------------------

>Cladosporium cladosporioides SL405T

--ACCTGATCCGAGGTCAA-
CCTTAGAAATGGGGTTGTTTTACGGCGTAGCC-T-----CCCG---AACACCCTTTAGCGAA--
TAGTTTCCAC--AACGCTTAGGGGACAGAA---GACCCAGC--
CGGTCGATTTGAGGCACGCGGCGGACC---------GCGTTG------------CCCAATACCA------------
-----AGCGAGG---CTTGAGTG-
GTGAAATGACGCTCGAACAGGCATGCCCCCCGGAATACCAGGGGGCGCAATGTGCGTTC
AAAGATTCGATGATTCACTGAATTCTGCAATTCACATTACTTATCGCATTTCGCTGCGTT
CTTCATCGATGCCAGAACCAAGAGATCCGTTGTTAAAAGTTTTA-ATTTATTAATTAA----
GTTTA-----------------CTCAGACTGCAA-------AGTTACG----------CAAGAGTTTGA--
AGTGTCCACCCGGAGCCCCC---GCCCGAAGGCAGGG-
TCGCCCCGGAGGCAACA-------
GAGTCGGACAACAAAGGGTTATGAACATCCCGGTGGTTAGACCGGGGTCACTTGTAATG
ATCCCTCCGCAGGTTCACCTACGGAGACCTTGTTACGACTTTTACTTCCA---------------------
----

>Cladosporium_cucumerinum_strain_CBS_HM148072.1

---CCTACCTGATCCGAGGTCAA-
CCTTAGAAATGGGGTTGTTTTACGGCGTAGCC-T-----CCCG---AACACCCTTTAGCGAA--
TAGTTTCCAC--AACGCTTAGGGGACAGAA---GACCCAGC--
CGGACGATTTGAGGCACGCGGCGGACC---------GCGTTG------------CCCAATACCA------------
-----AGCGAGG---CTTGAGTG-
GTGAAATGACGCTCGAACAGGCATGCCCCCCGGAATACCAGGGGGCGCAATGTGCGTTC
AAAGATTCGATGATTCACTGAATTCTGCAATTCACATTACTTATCGCATTTCGCTGCGTT
CTTCATCGATGCCAGAACCAAGAGATCCGTTGTTAAAAGTTTTA-ATTTATTAATTAA----
GTTTA-----------------CTCAGACTGCAA-------AGTTACG----------CAAGAGTTTGA--
AGTGTCCACCCGGAGCCCCC---GCCCGAAGGCAGGG-
TCGCCCCGGAGGCAACA-------GAGTCGGACAACAAAGGGTTATGAATAACCAGGCCGA-
AGCCCGGGCGTTCTTGTAATGATCCCTCCGCAGGTTCACCTACGGAGACCTTGTTACGAC
TTTTACTTCCTCTAAATGACCGGGT--

>Cladosporium cucumerinum AN187R

--CGAGGTCA--
CCTTAGAAATGGGGTTGTTTTACGGCGTAGCC-T-----CCCG---AACACCCTTTAGCGAA--
TAGTTTCCAC--AACGCTTAGGGGACAGAA---GACCCAGC--
CGGACGATTTGAGGCACGCGGCGGACC---------GCGTTG-----------CCCAATACCA-----------
-----AGCGAGG---CTTGAGTG-
GTGAAATGACGCTCGAACAGGCATGCCCCCCGGAATACCAGGGGGCGCAATGTGCGTTC
AAAGATTCGATGATTCACTGAATTCTGCAATTCACATTACTTATCGCATTTCGCTGCGTT
CTTCATCGATGCCAGAACCAAGAGATCCGTTGTTAAAAGTTTTA-ATTTATTAATTAA----
GTTTA----------------CTCAGACTGCAA-------AGTTACG----------CAAGAGTTTGA--
AGTGTCCACCCGGAGCCCCC--GCCCGAAGGCAGGG-
TCGCCCCGGAGGCAACA-------GAGTCGGACAACAAAGGGTTATGAATAACCAGGCCGA-
AGCCCGGGCGTTCTTGTAATGATCCCTCCGCAGGTTCACCTACGGAGACCTTGTTACGAT
TTTTT--

>gi|595827543|gb|JX681101.1|_Microsphaeropsis_olivacea_strain_CBS_303.68_18S_ribosomal_RNA_gene_partial_sequence_internal_transcribed_spacer_1_5.8S_ribosomal_RNA_gene_and_internal_transcribed_spacer_2e

---TAAAAAAGT-------
ACTTTTGGACG-TC--GTCGTT---ATGAGTGCAAAGCGCGAG-ATG---TAC--
TGCGCTCCGAAATCAATA-CGCCGGCTGC--
CAATTGTTTTAAGGCGAGTCTACACGCAAAG-GCGA--GACAA--------ACACCCAACACCA-
----------------AGCAAAG---CTTGAAGG-
TACAAATGACGCTCGAACAGGCATGCCCCATGGAATACCAAGGGGCGCAATGTGCGTTC
AAAGATTCGATGATTCACTGAATTCTGCAATTCACACTACTTATCGCATTTCGCTGCGTT
CTTCATCGATGCCAGAACCAAGAGATCCGTTGTTGAAAGTTGTA-ACTATTATGTTTT-----
T-CAGA---------CGCTGATTGCAACTGCAA----AGGGTTTAAA--T--
TGTCCAATCGGCGGGCG--AACCC-ACCGAGGAA--ACGTA-AGTACTCAAAAGA-
CATGGGTAAGAG---ATAGCAGGC---
----AAAGCCCACAA-
CTCTAGGTAATGATCCTTCCGCAGGTTCACCTACGGAAACCTTGTTACGACTT---------------
----

>Microsphaeropsis olivacea isolate LD50H

---TAAAAATGT-------
ACTTTTGGACG-TC--GTCGTT---ATGAGTGCAAAGCGCGAG-ATG---TAC--
TGCGCTCCGAAATCAATA-CGCCGGCTGC--
CAATTGTTTTAAGGCGAGTCTACACGCAAAG-GCGA--GACAA--------ACACCCAACACCA-
----------------AGCAAAG---CTTGAAGG-
TACAAATGACGCTCGAACAGGCATGCCCCATGGAATACCAAGGGGCGCAATGTGCGTTC
AAAGATTCGATGATTCACTGAATTCTGCAATTCACACTACTTATCGCATTTCGCTGCGTT
CTTCATCGATGCCAGAACCAAGAGATCCGTTGTTGAAAGTTGTA-ACTATTATGTTTT-----
T-CAGA---------CGCTGATTGCAACTGCAA----ATGGTTTAAA--T--
TGTCCAATCGGCGGGCG--AACCC-ACCGAGGAA--ACGTA-AGTACTCAAAAGA-

CATGGGTAAGAG---ATGGTAGGC---
----AAAGCCTACAA-CTCTAGGTAATGATCCTTCCGCAGGTTCACCTACGGA-------------------
----

>gi|350642907|gb|JN712497.1|_Microsphaeropsis_proteae_strain_CPC_1425_18S_ribosomal_RNA_gene_partial_sequence_internal_transcribed_spacer_1_5.8S_ribosomal_RNA_gene_and_internal_transcribed_spacer_2_coe

-----------------------------------TGCTTAAGTTCAGCGGGTATCCCTACCTGATCCGAGGTCAA-
GAGTGTAAAAATGT-------ACTTTTGGACG-TC--GTCGTT---ATGAGTGCAAAGCGCGAG-
ATG---TAC--TGCGCTCCGAAATCAATA-CGCCGGCTGC--
CAATTGTTTTAAGGCGAGTCTACACGCAAAG-GCGA--GACAA--------ACACCCAACACCA-
----------------AGCAAAG---CTTGAAGG-
TACAAATGACGCTCGAACAGGCATGCCCCATGGAATACCAAGGGGCGCAATGTGCGTTC
AAAGATTCGATGATTCACTGAATTCTGCAATTCACACTACTTATCGCATTTCGCTGCGTT
CTTCATCGATGCCAGAACCAAGAGATCCGTTGTTGAAAGTTGTA-ACTATTATGTTTT-----
T-CAGA---------CGCTGATTGCAACTGCAA----AGGGTTTAAA--T--
TGTCCAATCGGCGGGCG--AGCCC-ACCGAGGAA--ACGTA-AGTACTCAAAAGA-
CATGGGTAAGAG---ATAGCAGGC---
----AAAGCCCACAA-
CTCTAGGTAATGATCCTTCCGCAGGTTCACCTACGGAAACCTTGTTACGACTTTTACTTC
CTCTAAATGACCGAGTTTGACGAACTTTCCGGCTCGAAGTGGTCGTTGCCAACCTCTTCG
AGCCAGTCCGAAGGCCTCACTGAGCCATTCAATCGGTAGTAGCGACGGGCGGTGTGTAC
AAAG

>gi|162415147|gb|EU167567.1|_Boeremia_exigua_var._exigua_strain_CBS_118.94_small_subunit_ribosomal_RNA_gene_internal_transcribed_spacer_1_5.8S_ribosomal_RNA_gene_and_internal_transcribed_spacer_2_compe

---CCCTACCTGATCCGAGGTCAA-
GAGTGTAAAAAGTA-------CTTTTTGGACG-TC--GTCGTT---ATGAGTGCAAAGCGCGAG-
ATG---TAC--TGCGCTCCGAAATCAATA-CGCCGGCTGC--
CAATTGTTTTAAGGCGAGTCTACACGCAAAG-GCGA--GACAA--------ACACCCAACACCA-
----------------AGCAGAG---CTTGAAGG-
TACAAATGACGCTCGAACAGGCATGCCCCATGGAATACCAAGGGGCGCAATGTGCGTTC
AAAGATTCGATGATTCACTGAATTCTGCAATTCACACTACTTATCGCATTTCGCTGCGTT
CTTCATCGATGCCAGAACCAAGAGATCCGTTGTTGAAAGTTGTA-ACTATTATGTTTT-----
TTCAGA---------CGCTGATTTCAATTACAA----AGGGTTTAAG--T-
TTGTCCAATCGGCGGGCG--GACCC-GCCGAGGAA--ACGAA-GGTACTCAAAAGA-
CATGGGTAAGAG---ATGGTAGGC---
----AAAGCCTACAA-
CTCTAGGTAATGATCCTTCCGCAGGTTCACCTACGGAAACCTTGTTACGACTTTTACTTC
CTCTAAATGACCGAGTTTGACGAACTTTCCG---

>Phoma exigua isolate SL333T

---CTGATCCGAGGTCAA-
GAGTGTAAAAAGTA-------CTTTTTGGACG-TC--GTCGTT---ATGAGTGCAAAGCGCGAG-
ATG---TAC--TGCGCTCCGAAATCAATA-CGCCGGCTGC--
CAATTGTTTTAAGGCGAGTCTACACGCAAAG-GCGA--GACAA--------ACACCCAACACCA-
----------------AGCAGAG---CTTGAAGG-
TACAAATGACGCTCGAACAGGCATGCCCCATGGAATACCAAGGGGCGCAATGTGCGTTC
AAAGATTCGATGATTCACTGAATTCTGCAATTCACACTACTTATCGCATTTCGCTGCGTT
CTTCATCGATGCCAGAACCAAGAGATCCGTTGTTGAAAGTTGTA-ACTATTATGTTTT-----
TTCAGA---------CGCTGATTTCAATTACAA----AGGGTTTAAG--T-
TTGTCCAATCGGCGGGCG--GACCC-GCCGAGGAA--ACGAA-GGTACTCAAAAGA-
CATGGGTAAGAG---ATGGTAGGC---
----AAAGCCTACAA-
CTCTAGGTAATGATCCTTCCGCAGGTTCACCTACGGAAACCTTGTTACGATTTTTAC-------
----

>Pyrenochaetopsis_pratorum_strain_CBS_445.81_JF740263.1

---CCCTACCTGATCCGAGGTCA---
AACGTTGAAAAGG-------GCTTGTGGATG-CC--GTCGCG---GAGGGCAGAGACGCGGTA-
ATG---TGC--TGCGCTCTGCTGCCTATG-CGGCGGCTGC--
CAATAGCTTTTAGGTGAGTCCGCTGGCG----------GACAA--------GCACCCAACACCA---------
--------AGCATTG---CTTGAAGG-
TACAAATGACGCTCGAACAGGCATGCCCCATGGAATACCAAGGGGCGCAATGTGCGTTC
AAAGATTCGATGATTCACTGAATTCTGCAATTCACACTACTTTTCGCATTTCGCTGCGTT
CTTCATCGATGCCAGAACCAAGAGATCCGTTGTTGAAAGTTTTA-ATTATTGTTTTTT-----
T-CTGA---------CGCTGATTGCAACTACAA----AGGGTTTGTG--T-
TTGTCCTATCGGCAGGCA--AGCCC-GCCGAGGAA--ACAAGTGGTACGCAAAAGA-
CAAGGGTATAGC---AGGG---GCC--
--GAAGCCCGCTG-ATATTGATAATGAT--

>Ochrocladosporium_elatum_isolate_PS67_GU248334.1

---TCCTACCTGATCCGAGGTCAA-
GAGCAAAAGTGTAGG------CTTAATGGACG-C--AAGCGCT---CCAGTCGAGATGCGCAAA-
ATG---TGC--TGCGCTCCCAGGCTAGTA-CGCCGGCTGC--
CAATTGCTTTGAGGCGAGTCCACGCAAAAAG-CGG---GACAA--------ACACCCAACACCA--
---------------AGCAGAG---CTTGAAGG-
TACAAATGACGCTCGAACAGGCATGCCCCATGGAATACCAAGGGGCGCAATGTGCGTTC
AAAGATTCGATGATTCACTGAATTCTGCAATTCACACTACTTTTCGCATTTCGCTGCGTT
CTTCATCGATGCCAGAACCAAGAGATCCGTTGTTGAAAGTTGTA-ATTATTATGT--TT----
TTCTGA---------CGCTGATTGCAATTACAA----AAGGTTTAATAGT-G--
TCCTATTGGCAGGCA--AAGCCTACCAAGGAA--ACAAATAGTACGCAAAAGA-
CAAGGGT-------TCAGACAGG---GAC-------------------------CGAAGT----------------------------------
----------CCCCCCGATAAAATTG-GTAATGATCCTTCCGCAGGTWCACCTACAGAAACCT-
GTYACGACTTTT-ACTTCCAAAA--

>Ochrocladosporium elatum isolate SL464T

--CTGATCCGAGGTCA--
GAGCAAAAGTGTAGG------CTTAATGGACG-C--AAGCATT---CAAGCTGAGAGACGCAAA-
ATG---TGC--TGCGCTCCCAGGCTAGTA-CGCCGGCTGC--
CAATTGCTTTGAGGCGAGTCCACGCAGAAGG-CGG---GACAA--------ACACCCAACACCA--
---------------AGCAGAG---CTTGAAGG-
TACAAATGACGCTCGAACAGGCATGCCCCATGGAATACCAAGGGGCGCAATGTGCGTTC
AAAGATTCGATGATTCACTGAATTCTGCAATTCACACTACTTTTCGCATTTCGCTGCGTT
CTTCATCGATGCCAGAACCAAGAGATCCGTTGTTGAAAGTTGTA-ACTATTGTGT--TT----
TTCTGA---------CGCTGATTGCAATTACAA----AAGGTTTAATGGT-G--
TCCTATCGGCAGGCATAGAGCCCGCCGAGGAA--ACAAACAGTACGCAAAAGA-
CAAGGGT-------TCAGACAGG---GAC------------------------CGAAGT-----------------------------------
----------CCCCC-GATAAAATTG-
GTAATGATCCTTCCGCAGGTTCACCTACGGAAACCTTGTTACGACTTTTTACTTCCCA-----
----

>GU014950.1|_Epicoccum_CBS161-73Group2Lim

--GAGTGTAAAAATGT-------
ACTTTTGGACG-TC--GTCGTT---GTGAGTGCAAAGCGCGAG-ATG---TAC--
TGCGCTCCGAAATCAATA-CGCCGGCTGC--
CAATTGTTTTAAGGCGAGTCTACACGCAGAG-GCGA--GACAA--------ACACCCAACACCA-
----------------AGCAGAG---CTTGAAGG-
TACAAATGACGCTCGAACAGGCATGCCCCATGGAATACCAAGGGGCGCAATGTGCGTTC
AAAGATTCGATGATTCACTGAATTCTGCAATTCACACTACTTATCGCATTTCGCTGCGTT
CTTCATCGATGCCAGAACCAAGAGATCCGTTGTTGAAAGTTGTA-ACTATTATGTTTT-----
TTCAGA---------CGCTGATTGCAACTGCAA----AGGGTTTGAA--
TGTTGTCCAATCGGCGGGCG--GACCC-GCCGAGGAA--ACGAA-GGTACTCAAAAGA-
CATGGGTAAGAG---GTAGCAGACC--
-----GAAGTCTACAAACTCTAG--

>Epicoccum nigrum isolate SL332T

--CTGATCCGAGGTCA--GAGTGTAAAAATGT-
------ACTTTTGGACG-TC--GTCGTT---GTGAGTGCAAAGCGCGAG-ATG---TAC--
TGCGCTCCGAAATCAATA-CGCCGGCTGC--
CAATTGTTTTAAGGCGAGTCTACACGCAGAG-GCGA--GACAA--------ACACCCAACACCA-
----------------AGCAGAG---CTTGAAGG-
TACAAATGACGCTCGAACAGGCATGCCCCATGGAATACCAAGGGGCGCAATGTGCGTTC
AAAGATTCGATGATTCACTGAATTCTGCAATTCACACTACTTATCGCATTTCGCTGCGTT
CTTCATCGATGCCAGAACCAAGAGATCCGTTGTTGAAAGTTGTA-ACTATTATGTTTT-----
TTCAGA---------CGCTGATTGCAACTGCAA----AGGGTTTGAA--
TGTTGTCCAATCGGCGGGCG--GACCC-GCCGAGGAA--ACGAA-GGTACTCAAAAGA-
CATGGGTAAGAG---GTAGCAGACC--

GAAGTCTACAAACTCTAGGTAATGATCCTTCCGCAGGTTCACCTACGGAAACCTTGTTAC

GATTTTTACTTCCA---

>Diaporthe_phaseolorum_2734_EU272538.1

--CAGAAGTTGGGG-
GTTTAACGGCAGGGCA-CCG---CCAG---GGCCTTCCAGAGCGAGGGTTTA-ACTAC--
TGCGCTCGG--GGTCCTG-GCGAGCTCGC--CACTAGATTTCAG--GGCCTGCTCCCTCGC-----
GGAAGCAGTG--------CCCCATCACCA-----------------AGCCAGG---CTTGAGGG-
TTGAAATGACGCTCGAACAGGCATGCCCTCCGGAATACCAGAGGGCGCAATGTGCGTTC
AAAGATTCGATGATTCACTGAATTCTGCAATTCACATTACTTATCGCATTTCGCTGCGTT
CTTCATCGATGCCAGAACCAAGAGATCCGTTGTTGAAAGTTTTG-ATTCATTTATGT----
TTTTTT-----------------CTCAGAGTTTCA------GTGTAAAA---------ACAAGAGTTAA---
CTTGGCCGCCGGCGGGCTGCTC--------CTCGTTA----------CCGAGGGGCCC-----CTG--
TGAGGAGGCCGGC-CTGACGCCGAGGCAACAGT--
AAGGTATAAGTTCACAAAGGGTTTCTGGGTGCGCC--------
GAGGCGCGTTCCAGCAATGATCCCTCCGCTGGTTCACCAACGGA---------------------------------
----

>Diaporthe phaseolorum LD46H

--CAGAAGTTGGGG-
GTTTAACGGCAGGGCA-CGG---CCAG---GGCCTTCCAGAGCGAGGGTTTA-ACTAC--
TGCGCTCGG--GGTCCTG-GCGAGCTCGC--CACTAGATTTCAG--GGCCTGCTCCCTCGC-----
GGAAGCAGTG--------CCCCATCACCA-----------------AGCCAGG---CTTGAGGG-
TTGAAATGACGCTCGAACAGGCATGCCCTCCGGAATACCAGAGGGCGCAATGTGCGTTC
AAAGATTCGATGATTCACTGAATTCTGCAATTCACATTACTTATCGCATTTCGCTGCGTT
CTTCATCGATGCCAGAACCAAGAGATCCGTTGTTGAAAGTTTTG-ATTCATTTATGT----
TTTTTTT----------------CTCAGAGTTTCA------GTGTAAAA---------ACAAGAGTTAA---
CTTGGCCGCCGGCGGGCTGCTC--------CTCGTCA----------CCGAGGGGCCT-----CTG--
TGAGGAGGCCGGC-CTAACGCCGAGGCAACAAT--
AAGGTATAAGTTCACAAAGGGTTTCTGGGTGCGCC--------
GAGGCGCGTTCCAGCAATGATCCCTCCGCTGGTTCACCAACGGA---------------------------------
----

>gi|469783301|gb|KC343073.1|_Diaporthe_eres_strain_CBS_101742_18S_ribosomal_RNA_gene_partial_sequence_internal_transcribed_spacer_1_5.8S_ribosomal_RNA_gene_and_internal_transcribed_spacer_2_complete_se

--TTCCTACCTGATCCGAGGTCAAAT-
TTTCAGAAGTTGGGG-GTTTAACGGCAGGGCA-CCG---CCAG---
GGCCTTCCAGAGCGAGGGTTTA-ACTAC--TGCGCTCGG--GGTCCTG-GCGAGCTCGC--
CACTGAATTTCAG--GGCCTGCTTCTTGGGT---AAGAAGCAGTG--------CCCCATCACCA-----
------------AGCCAGG---CTTGAGGG-
TTGAAATGACGCTCGAACAGGCATGCCCTCCGGAATACCAGAGGGCGCAATGTGCGTTC
AAAGATTCGATGATTCACTGAATTCTGCAATTCACATTACTTATCGCATTTCGCTGCGTT
CTTCATCGATGCCAGAACCAAGAGATCCGTTGTTGAAAGTTTTG-ATTCATTTATGT----
TTTGTG-----------------CTCAGAGTTTCA------GTGTAAAA---------ACAAGAGTTAG---

GTTGGCCGCCGACGGGCTGT----------CTCAACA----------CCCGAGGGTGA-----
GGGGCCCCGAGGGACCAG-CTAGCGCCGAGGCAACAGT--
AAGGTATAAGTTCACAAAGGGTTTCTGGGTGCGCCT-------
GGGGCGCGTTCCAGCAATGATCCCTCCGCTGGTTCACCAACGGAGACCTTGTT-------------
----
-

>Diaporthe eres isolate SL473T

---CTGATCCGAGGTCAAT-
TTTCAGAAGTTGGGG-GTTTAACGGCAGGGCA-CCG---CCAG---
GGCCTTCCAGAGCGAGGGTTTA-ACTAC--TGCGCTCGG--GGTCCTG-GCGAGCTCGC--
CACTGAATTTCAG--GGCCTGCTTCTTGGGT---AAGAAGCAGTG--------CCCCATCACCA-----
------------AGCCAGG---CTTGAGGG-
TTGAAATGACGCTCGAACAGGCATGCCCTCCGGAATACCAGAGGGCGCAATGTGCGTTC
AAAGATTCGATGATTCACTGAATTCTGCAATTCACATTACTTATCGCATTTCGCTGCGTT
CTTCATCGATGCCAGAACCAAGAGATCCGTTGTTGAAAGTTTTG-ATTCATTTATGT----
TTTGTG-----------------CTCAGAGTTTCA------GTGTAAAA---------ACAAGAGTTAG---
GTTGGCCGCCGACGGGCTGT----------CTCAACA----------CCCGAGGGTGA-----
GGGGCCCCGAGGGACCAG-CTAGCGCCGAGGCAACAGT--
AAGGTATAAGTTCACAAAGGGTTTCTGGGTGCGCCT-------
GGGGCGCGTTCCAGCAATGATCCCTCCGCTGGTTCACCAACGGAGACCTTGTTACGATTT
TTAC--

>Phomopsis_mali_IFO31031_AB665315.1

--CAGAAGTTGGGG-
GTTTAACGGCAGGGCA-CCG---CCAG---GGCCTTCCAGAGCGAGGGTTTA-ACTAC--
TGCGCTCGG--GGTCCTG-GCGAGCTCGC--CACTGAATTTCAG--GGCCTGCTTCTTGGGT---
AAGAAGCAGTG--------CCCCATCACCA----------------AGCCAGG---CTTGAGGG-
TTGAAATGACGCTCGAACAGGCATGCCCTCCGGAATACCAGAGGGCGCAATGTGCGTTC
AAAGATTCGATGATTCACTGAATTCTGCAATTCACATTACTTATCGCATTTCGCTGCGTT
CTTCATCGATGCCAGAACCAAGAGATCCGTTGTTGAAAGTTTTG-ATTCATTTATGT----
TTTATT-----------------CTCAGAGTTTCA------GTGTAAAA---------ACAAGAGTTGG---
GTTGGCCGCCGGCGGGCTGT----------CTCAACA----------CCCGAGGGTGA-----
GGGGCCCCGAAGGACCAG-CTAGCGCCGAGGCAACAGT--
AAGGTATAAGTTCACAAAGGGTTTCTGGGTGCGCCT-------
GGGGCGCGTTCCAGCAATGATCCCTCCGCTGGTTCACCAACGGA------------------------------
----

>Phomopsis mali isolate LD14H

--CAGAAGTTGGGG-
GTTTAACGGCAGGGCA-CCG---CCAG---GGCCTTCCAGAGCGAGGGTTTA-ACTAC--
TGCGCTCGG--GGTCCTG-GCGAGCTCGC--CACTGAATTTCAG--GGCCTGCTTCTTGGGT---
AAGAAGCAGTG--------CCCCATCACCA----------------AGCCAGG---CTTGAGGG-
TTGAAATGACGCTCGAACAGGCATGCCCTCCGGAATACCAGAGGGCGCAATGTGCGTTC
AAAGATTCGATGATTCACTGAATTCTGCAATTCACATTACTTATCGCATTTCGCTGCGTT

CTTCATCGATGCCAGAACCAAGAGATCCGTTGTTGAAAGTTTTG-ATTCATTTATGT----
TTTGTG-----------------CTCAGAGTTTCA------GTGTAAAA---------ACAAGAGTTGG---
GTTGGCCGCCGGCGGGCTGT----------CTCAACA----------CCCGAGGGTGA-----
GGGGCCCCGGAGGACCAG-CTAGCGCCGAGGCAACATT--
AAGGTATAAGTTCACAAAGGGTTTCTGGGTGCGCCT-------
GGGGCGCGTTCCAGCAATGATCCCTCCGCTGGTTCACCAACGGA-------------------------------
----

>gi|21310048|gb|AF377292.1|_Pestalotiopsis_microspora_strain_CBS364.54_18S_ribosomal_RNA_gene_partial_sequence_internal_transcribed_spacer_1_5.8S_ribosomal_RNA_gene_and_internal_transcribed_spacer_2_ce

--TCCTACCTGATCCGAGGTCAACCA-C--
AAAAAATTGGGG-GTTTAGCGGCTGGGAG-TTA---TAGC---ACCTAACAAAAGCGAGAA-
AAAAATTAC--TACGCTCAGA-GGATACT-ACAAATCCGC--CGTTGTATTTCAG--
GAACTACAACTCCTAA---GAGAAGTAGAT--------TCCCAACACTA-------------AGC----
TAGG--CTTAAGGG-
TTGAAATGACGCTCGAACAGGCATGCCCACTAGAATACTAATGGGCGCAATGTGCGTTC
AAAGATTCGATGATTCACTGAATTCTGCAATTCACATTACTTATCGCATTTCGCTGCGTT
CTTCATCGATGCCAGAACCAAGAGATCCGTTGTTGAAAGTTTTG-ACTTATTAAAAT------
AAGACG---------------CTCAGA--TTAC------ATAAAATA---------ACAAGAGTTTA---
ATGGTCCACCGGCAGC-------------AGCTATAAGAAGAC-------------CTATAA------------------
CTTCTGCCGAGGCAACA-----AAAGGTAAGTTCACAT--GGGTTGGGAG-------------
TTTAGAAAACTCTATAATGATCCCTCCGCTGGTTCACCAACGGAGACCTTGTTACGACTT
TTACTTCCA--

>Pestialotiopsis microspora isolate AN325T

---TGATCCGAGGTCA-CCA-C--
AAAAAATTAGGG-GTTTAGCGGCTGGGAG-TTA---CAGC---ACCTGACAAAAGCGAGAA-
AAAAATTAC--TACGCTCAGA-GGACACT-ATAAATCCGC--CGTTGTATTTCAG--
GAACTACGACTCCTAG---AAGAAGTAGAT--------TCCCAACACTA-------------AGC----
TAGG--CTTAAGGG-
TTGAAATGACGCTCGAACAGGCATGCCCACTAGAATACTAATGGGCGCAATGTGCGTTC
AAAGATTCGATGATTCACTGAATTCTGCAATTCACATTACTTATCGCATTTCGCTGCGTT
CTTCATCGATGCCAGAACCAAGAGATCCGTTGTTGAAAGTTTTG-ACTTATTAAAAT------
AAGACG---------------CTCAGA--TTCC------ATAAAATA---------ACAAGAGTTTA---
GTGGTCCACCGGCAGC-------------AGCTATAAGAAGTC-------------CTATAA------------------
CTTCTGCCGAGGCAACA-----AAAGGTAAGTTCACAT--GGGTTGGGAG-------------
TTTAGAAAACTCTATAATGATCCCTCCGCTGGTTCACCAACGGAGACCTTGTTACGATTT
TTAC---

>gi|121622475|gb|EF063140.1|_Sarcopodium_oculorum_internal_transcribed_spacer_1_partial_sequence_5.8S_ribosomal_RNA_gene_complete_sequence_and_internal_transcribed_spacer_2_partial_sequence

---TTCAGAATTGGGGG-
GTTTAACGGCCGGGAA-C-----CGCC---ACACTTCCAAAGCGAG---AGAGATTAC--
TACGCTCGG--AGTTATA-GCGAGCCCGC--CACTGTTTTTCAG--GGCCTGCGGCC------
GAAGCCGCAGAAG--------CCCCAACACCA-----------------AGCAAGAGAGCTTGAGGG-
TTGAAATGACGCTCGAACAGGCATGCCCACTAGAATACTAATGGGCGCAATGTGCGTTC
AAAGATTCGATGATTCACTGAATTCTGCAATTCACATTACTTATCGCATTTCGCTGCGTT
CTTCATCGATGCCAGAACCAAGAGATCCGTTGTTGAAAGTTTTA-ACT-ATTAAAAG------
TTTA-----------------CTCAGACAGAA-------CAGTAATT--------AACAAGAGTTTT----
AGATACTCCGGCAGGCACC----------------------------ACGGATGGCT------
AAGGCCCCGAAGAGCCG---CCTGCCGAAGCAACGAA--ACAGGTA-GATAAACAAA-
TGGTTTGGGA--------------GTTTTGCAACTCTTTAATGAT--

-

>Sarcopodium oculorum isolate LD185H

---GAAAATTGGGGG-
GTTTAACGGCCGGGAA-C-----CGCC---ACACTTCCAAAGCGAG---AGAGATTAC--
TACGCTCGG--AGTTATA-GCGAGCCCGC--CACTGTTTTTCAG--GGCCTGCGGCC------
AAAGCCGCAGAAG--------CCCCAACACCA-----------------AGCAAGA--GCTTGAGGG-
TTGAAATGACGCTCGAACAGGCATGCCCACTAGAATACTAATGGGCGCAATGTGCGTTC
AAAGATTCGATGATTCACTGAATTCTGCAATTCACATTACTTATCGCATTTCGCTGCGTT
CTTCATCGATGCCAGAACCAAGAGATCCGTTGTTGAAAGTTTTA-ACT-ATTAAAAG------
TTTA-----------------CTCAGACAGAA-------CAGTAATT--------AACAAGAGTTTT----
AGATACTCCGGCAGGCACC----------------------------ACGGATGGCT------
AAGGCCCCGAAGAGCCG---CCTGCCGAAGCAACGAA--ACAGGTA-GATAAACAAA-
TGGTTTGGGA--------------
GTTTTGCAACTCTTTAATGATCCCTCCGCTGGTTCACCAACGGA----------------------------------
----

>Eutypa_consobrina_CBS122678_EU552125.1

---GAAAAATAGGGG-
GTTTAGCGGCCGGACG-CCAGCGCTGG---AGCCACCAGAAGCGAGG--AAAAATTAC--
TACGCTTGG--GGCCAAC-ACGACTCCGC--CGATGCTTTTCAG--GAAGTA----TCCGAA---
GA----TACAC--------TCCCAACACCA--------------AGCAGCTAGGG--CTTGAGGG-
TTGAAATGACGCTCGAACAGGCATGCCCACTAGAATACTAATGGGCGCAATGTGCGTTC
AAAGATTCGATGATTCACTGAATTCTGCAATTCACATTACTTATCGCATTTCGCTGCGTT
CTTCATCGATGCCAGAACCAAGAGATCCGTTGTTGAAAGTTTTA-ACTTATTAAGTA------
TAAAAA---------------ATCAGA-AGTTC------C-ACTATA---------ACAAGAGTTTT---
ACTGTCCGCCGGCGGGTCGGCCTGCAGCGGGCTACAGGGTAGCTCGCTCCCGGGTCCCT
ACAGGGTAGGTACCGGGTAGGCTTCCCCGCCGAGGCAACA-----TA-GGTAAGTTCACAT--
GGGTTTGGGA--------------
GTTAGATAACTCTGTAATGATCCCTCCGCTGGTTCACCAACGGA--------------------------------
----

>Eutypa consorbrina isolate LD54H

--GAAAGATAGGGG-
GTTTAGCGGCCGGACG-CCAGCGCTGG---AACCACCAGAAGCGAGG--AAAAATTAC--
TACGCTTGG--GGCCAAC-ACGACTCCGC--CGATGCTTTTCAG--GAAGTA----TCCGAA---
GA----TACAC--------TCCCAACACCA--------------AGCAGCTAGGG--CTTGAGGG-
TTGAAATGACGCTCGAACAGGCATGCCCACTAGAATACTAATGGCGCAATGTGCGTTC
AAAGATTCGATGATTCACTGAATTCTGCAATTCACATTACTTATCGCATTTCGCTGCGTT
CTTCATCGATGCCAGAACCAAGAGATCCGTTGTTGAAAGTTTTC-ACTTATTAAGTA------
TAAAAA---------------ATCAGA-AGTTC------C-ACTATA---------ACAAGAGTTTT---
ACTGTCCGCCGGCGGGTCGGCCTGCAGCGGGCTACAGGGTAGCTCGCTCCCGGGTCCCT
ACAGGGTAGGTACCGGGTAGGCTTCCCCGCCGAGGCAACA-----TA-GGTAAGTTCACAT--
GGGTTTGGGA--------------
GTTAGATAACTCTGTAATGATCCCTCCGCTGGTTCACCAACGGA---------------------------------
----

>Eutypa_lata_strain_UCD2275MO_HQ288221.1

---CCTACCTGATCCGAGGTCAACCA-
TTTAAAAAATTGGGG-GTTTAACGGCAAGACG-TCAGCCGTGA---CAC-
ACCTAAAGCGAG---AAGAATTAC--TACGCTTGG--GGTCACC-GCGACTCCGC--
CAATGCTTTTGAG--GAGCTA----TCCGGA---GA----TAGGC--------TCCCAACACCA--------------
-AGCAGCTAGGG--CTTGAAGG-
TCGAAATGACGCTCGAACAGGCATGCCCACTAGAATACTAATGGCGCAATGTGCGTTC
AAAGATTCGATGATTCACTGAATTCTGCAATTCACATTACTTATCGCATTTCGCTGCGTT
CTTCATCGATGCCAGAACCAAGAGATCCGTTGTTGAAAGTTTTA-ACTTATTAAGTA------
TAAACA---------------CTCAGATAATCA------C-TAAAAA---------ACAAGAGTTTA---
AGTGTCCACCGGCGGGTAGGCCTGCAGCGGGCTACAGGGTAGCTCGCTCCCGGGTAGCT
ACAGGGTAGGTACCGGGTAGGCTTCCCCGCCGAGGCAACA-----TA-GGTAAGTTCACAT--
GGGTTTGG-A--------------
GTTAGGTAACTCTGTAATGATCCCTCCGCTGGTTCACCAACGGAGAC---------------------------
----

>Eutypa lata isolate LD366H

---ACCTGATCCGAGGTCAACCA-
TTTAAAAAATTGGGG-GTTTAACGGCAAGACG-TCAGCCGTGA---CAC-
ACCTAAAGCGAG---AAGAATTAC--TACGCTTGG--GGTCACC-GCGACTCCGC--
CAATGCTTTTGAG--GAGCTA----TCCGGA---GA----TAGGC--------TCCCAACACCA--------------
-AGCAGCTAGGG--CTTGAAGG-
TCGAAATGACGCTCGAACAGGCATGCCCACTAGAATACTAATGGCGCAATGTGCGTTC
AAAGATTCGATGATTCACTGAATTCTGCAATTCACATTACTTATCGCATTTCGCTGCGTT
CTTCATCGATGCCAGAACCAAGAGATCCGTTGTTGAAAGTTTTA-ACTTATTAAGTA------
TAAACA---------------CTCAGATAATCA------CTAAAAAA---------ACAAGAGTTTA---
AGTGTCCACCGGCGGGTAGGCCTGCAGCGGGCTACAGGGTAGCTCGCTCCCGGGTAGCT
ACAGGGTAGGTACCGGGTAGGCTTCCCCGCCGAGGCAACA-----TA-GGTAAGTTCACAT--
GGGTTTGG-A--------------
GTTAGGTAACTCTGTAATGATCCCTCCGCTGGTTCACCAACGGAGACCTTGTTACGATTT
TTTA--

>gi|94384810|dbj|AB258367.1|_Cordyceps_brongniartii_genes_for_18S_rRNA_ITS1_5.8S_rRNA_ITS2_28S_rRNA_partial_and_complete_sequence_strain:_BCMU_BB06

---CAGAAGTTGGGT-
GTTTTACGGC-GTGGC-CG----CGTC---GGGGTTCCGGTGCGAG-TTGGA--TTAC--
TACGCAGAGGTCGCCGCG-GACGGGCCGC--CACTCCATTTCAG--GGCCGGCGGT--------
GTGCTGCCGGT---------CCCCAACGCCG----------ACTTCCCCAAAGGGAG-GTCGAGGG-
TTGAAATGACGCTCGAACAGGCATGCCCGCCAGAATGCTGGCGGGCGCAATGTGCGTTC
AAAGATTCGATGATTCACTGGATTCTGCAATTCACATTACTTATCGCATTTCGCTGCGTT
CTTCATCGATGCCAGAGCCAAGAGATCCGTTGTTGAAAGTTTTG-ATTTATTTGTTTTGC-
CTTGCGGCGTA------------TTCAGAAGATG-------CTGATAAT---------ACAAGAGTTTG----
AGGGTCCCCGGCGGCCGCT---------------GG----------TCCAGTCCGC-------
GTCCGGCTGGGGCGAG----TCCGCCGAAGCAACAA----TAGGTA-
GGTTCACATAAGGGTTAGGGA--------------GTTGAA-
AACTCGGTAATGATCCCTCCGCTGGTTCACCAACGGA--

>Cordiceps brongniartii isolate LD144H

--CCTGATTCGAGGTCAC-
GTTCAGAAGTTGGGT-GTTTTACGGC-GTGGC-CG----CGTC---GGGGTTCCGGTGCGAG-
TTGGA--TTAC--TACGCAGAGGTCGCCGCG-GACGGGCCGC--CACTCCATTTCAG--
GGCCGGCGGT--------GTGCTGCCGGT---------CCCCAACGCCG----------
ACTTCCCCAAAGGGAG-GTCGAGGG-
TTGAAATGACGCTCGAACAGGCATGCCCGCCAGAATGCTGGCGGGCGCAATGTGCGTTC
AAAGATTCGATGATTCACTGGATTCTGCAATTCACATTACCTATCGCATTTCGCTGCGTT
CTTCATCGATGCCAGAGCCAAGAGATCCGTTGTTGAAAGTTTTG-ATTTATTTGTTTTGC-
CTTGCGGCGTA------------TTCAGAAGATG-------CTGATAAT---------ACAAGAGTTTG----
AGGGTCCCCGGCGGCCGCT---------------GG----------TCCAGTCCGC-------
GTCCGGCTGGGGCGAG----TCCGCCGAAGCAACAA----TAGGTA-
GGTTCACATAAGGGTTAGGGA--------------GTTGAA-
AACTCGGTAATGATCCCTCCGCTGGTTCACCAACGGAGACCTTGTTACGATTTTACTTC
CA--

>Verticillium_cf._biguttatum_CBS_EF641856.1

---CTACCTGATCCGAGGTCAACCAT-
TGGTGTAGGTCGGGG-GTTTTACGGC-GTGGC-CG----CACC---
GCTCTCCGCATGCGAGGTTAAG--TTAC--TACGCAGAGGAGGCCGCG-ATGGGGCCGCA-
CACTAGATTTCGG--GGGCGGCCGC---------GGAGGCCGGT---------
CCCCAACACCGGGATGC---AACCCTCCGAAGAGGGGTCCCGAGGG-
TTGAAATGACGCTCGGACAGGCATGCCCGCCAGAGTACTGGCGGGCGCAATGTGCGTTC
AAAGATTCGATGATTCACTGAATTCTGCAATTCACATTACTTATCGCATTTCGCTGCGTT
CTTCATCGATGCCAGAACCAAGAGATCCGTTGTTGAAAGTTTTG-ATTTATTTGTT---
TGCTTTCGCCA--------------CTCAGAAGATA-------CCGCTATGGG------AAACAGAGTGTT---
TAGGTCCTCCGGCGGCCGTCTG-------------GA----------
TCCGGGACACCTGTGAGGGGCGCCCGGGACAGA----ACCGCCGAAGCAACTG----
TAGGTA-TGTTCACAT--GGGTTTGGGA--------------GTTGTA-AACTCGGTAAGTATCCCT----

----

>Verticillium cf biguttatum isolate AN130T

--ACCTGATCCGAGGTCA-CCAT-
TGGTGTAGGTCGGGG-GTTTTACGGC-GTGGC-CG----CACC---
GCTCTCCGCATGCGAGGTTAAG--TTAC--TACGCAGAGGAGGCCGCG-ATGGGGCCGCA-
CACTAGATTTCGG--GGGCGGCCGC---------GGAGGCCGGT---------
CCCCAACACCGGGATGC---AACCCTCCGAAGAGGGGTCCCGAGGG-
TTGAAATGACGCTCGGACAGGCATGCCCGCCAGAGTACTGGCGGGCGCAATGTGCGTTC
AAAGATTCGATGATTCACTGAATTCTGCAATTCACATTACTTATCGCATTTCGCTGCGTT
CTTCATCGATGCCAGAACCAAGAGATCCGTTGTTGAAAGTTTTG-ATTTATTTGTT---
TGCTTTCGCCA--------------CTCAGAAGATA-------CCGCTATGGG------AAACAGAGTGTT---
TAGGTCCTCCGGCGGCCGTCTG-------------GA----------TCCGGGACACCTGTAAGGG-
CGCCCGGGACAGA----ACCGCCGAAGCAACTG----TAGGTA-TGTTCACAT--
GGGTTTGGGA--------------GTTGTA-
AACTCGGTAATGATCCCTCCGCTGGTTCACCAACGGAGACCTTGTTACGATTTTTACTTC
CCACCC--

>Marine_ascomycete_SAP162_AF422992.1

---CG-GAGGGGGCTCCAGGA-
AGTTTTGGGCTGAGAC-GG-----GAA---GGTCATCCAATACGAGATTTAG--TTAC--
CACGTAGGGGAAACTCGACACCGTTTCGC--
CGCTGCATTTGAGCCGCGCGGGGGTGGGGAGCCCGGCCGTTAGGCCGGATCGCCGCCGT
ACCCGCGTCGG---CCAACACCAAGCCGGA--CTTGAGTG-
GTGAGATGACGCTCGGACAGGCATGCCCGCCGGAATACCGGCGGGCGCAATGTGCGTTC
AAAGATTCGATGATTCACTGAATTCTGCAATTCACATTACTTATCGCATTTCGCTGCGTT
CTTCATCGATGCCAGAACCAAGAGATCCATTGTTGAAAGTTTTA-ACTTATTTGTGT------
CACA-----------------CTCAGACTTCCA------GTGCAAAAGCGTAG--ACGTTGAGTTTG-----
AGACCTCCGGCGGGCCGGTCT-------
AAGGCGGGCCAGGCCGCCCCGGAGCGCCGAAGCGCAAGGGGGCGGACCGGCCAGCCCG
CCGAAGCAACTGT--AATAGGTATGTTCACAGGGGGTTTTGAGC-----------------GTAGAA-
TCGGTAATGATCCCTCCGCTGGTTCACCAACGGA--

>Moheitospora sp. isolate AN129R

--TCCGAGGTCAACCTTGGTGGGCCA-
CAGAAGCAGCCCATT-AGTTTTGGGCAGAGAC-GG-----AAA---GG-
CTTCCAATACGAGATTATA--TTAC--CACGTAGGGGAAACTCGACACCGTTTCGC--
CGCTGAAATTGGGCCGCGCGG---------------------------------CCGGAGCCGCGTCGG---
CCAACACCAAGACAGG--CTTGAGGG-
GTGAGATGACGCTCGGACAGGCATGCCCGCCGGAATACCGGCGGGCGCCATGTGCGTTC
AAAGATTCGATGATTCACTGAATTCTGCAATTCACATTACTTATCGCATTTCGCTGCGTT
CTTCATCGATGCCAGAACCAAGAGATCCATTGTTGAAAGTTTTA-ACTTATTTGTAT------

GACA-----------------CTCAGATTTGAA------CAATAAAGGAGTG----CGCTGAGTTTA-----
AGACCTCCGGCGGGCCTGTCT-------G-GGCGG--
CCGGCGTCTCGACGGCACCGGAGCACGCGTGGAGAGACCGAC-
AGCCCGCCGAAGCAACTGG--AATAGGTATGTTCACAGGGGGTTTTGAGT-----------------
GTAGAACTCGGTAATGATCCCTCCGCTGGTTC-
CCAACGGAGACCTTGTTACGACTTTTTACTTCCCATA---

>Tolypocladium_cylindrosporum_AB208110.1

---GAGAAGTTGGGC-
GTTTTACGGC-GTGAC-CGT---CTCC---GCGCTCC-GGTGCGAGGTTGTG--CTAC--
TACGCAGGGGAGGCTGCG-GCGAGGTCGC--CACTGCATTTGGG--GGGCGGCCG------------
GGGCCGGT---------CCCCAACACCA----------AGCCGGCTG--GGG---CTTGAGGG-
TTGAAATGACGCTCGAACAGGCATGCCCGCCAGAATACTGGCGGGCGCAATGTGCGTTC
AAAGATTCGATGATTCACTGAATTCTGCAATTCACATTACTTATCGCATTTCGCTGCGTT
CTTCATCGATGCCAGAGCCAAGAGATCCGTTGTTGAAAGTTTTG-ATTCATTTTT------
CTTGTGAGA--------------CTCAGAATATG-------CCACTATGGTTTA---AACAAGAGTTTG-----
GGTCCTCCGGCGGGCGCCTG-------------GT----------TCCGGGACGCC-----
GAGGGCGCCGGGGCGG----TCCCGCCGAAGCAACG----TTGGGTA-TGTTCACAG--
GGGTTTGGGA--------------GTTGAT-
AACTCGGTAATGATCCCTCCGCTGGTTCACCAACGGA---

>Tolypocladium cylindrosporum isolate LD150F

---GAGAAGTTGGGC-
GTTTTACGGC-GTGAC-CGT---CTCC---GCGCTCC-GGTGCGAGGTTGTG--CTAC--
TACGCAGGGGAGGCTGCG-GCGAGGTCGC--CACTGCATTTGGG--GGGCGGCCG------------
GGGCCGGT---------CCCCAACACCA----------AGCCGGCTG--GGG---CTTGAGGG-
TTGAAATGACGCTCGAACAGGCATGCCCGCCAGAATACTGGCGGGCGCAATGTGCGTTC
AAAGATTCGATGATTCACTGAATTCTGCAATTCACATTACTTATCGCATTTCGCTGCGTT
CTTCATCGATGCCAGAGCCAAGAGATCCGTTGTTGAAAGTTTTG-ATTCATTTTT------
CTTGTGAGA--------------CTCAGAATATG-------CCACTATGGTTTA---AACAAGAGTTTG-----
GGTCCTCCGGCGGGCGCCTG-------------GT----------TCCGGGACGCC-----
GAGGGCGCCGGGGCGG----TCCCGCCGAAGCAACG----TTGGGTA-TGTTCACAG--
GGGTTTGGGA--------------GTTGAT-
AACTCGGTAATGATCCCTCCGCTGGTTCACCAACGGA---

>gi|54292654|gb|AY605724.1|_Hypocrea_lixii_strain_DAOM_220786_18S_ribosomal_RNA_gene_partial_sequence_internal_transcribed_spacer_1_5.8S_ribosomal_RNA_gene_and_internal_transcribed_spacer_2_complete_se

---CAGAAGTTGGGT-
GTTTAACGGCTGTGGA-CG----CGCC---GCGCTCCCGATGCGAGTGTGCAAACTAC--
TGCGCAGGAGAGGCTGCG-GCGAGACCGC--CACTGTATTTCGG--
AGACGGCCACCCGCTAAGGGAGGGCCGAT---------CCCCAACGCCG----------
ACCCCCCGGAGGGG---TTCGAGGG-

TTGAAATGACGCTCGGACAGGCATGCCCGCCAGAATACTGGCGGGCGCAATGTGCGTTC
AAAGATTCGATGATTCACTGAATTCTGCAATTCACATTACTTATCGCATTTCGCTGCGTT
CTTCATCGATGCCAGAACCAAGAGATCCGTTGTTGAAAGTTTTG-
ATTCATTTTCGAAACGCCTACGAGAGGCGCCGAGAAAGGCTCAGATTATAAAAAAACCC
GCGAGGGGGTATACAATAAGAGTTTTAGGTTGGTCCTCCGGCGGGCGCCTT------------
GG----------TCCGGGGCTGC-----GACGCACCCGGGGCAGAG-ATCCCGCCGAGGCAACAGT--
TTGGTAA-CGTTCACATT-GGGTTTGGGA--------------GTTGTA-
AACTCGGTAATGATCCCTCCGCTGGTTCACCAACGGAGACCTTGTTACGACTTTTACTTC
CA--

>Hypocrea lixii isolate LD8H

---CAGAAGTTGGGT-
GTTTAACGGCTGTGGA-CG----CGCC---GCGCTCCCGATGCGAGTGTGCAAACTAC--
TGCGCAGGAGAGGCTGCG-GCGAGACCGC--CACTGTATTTCGG--
AGACGGCCACCCGCTAAGGGAGGGCCGAT---------CCCCAACGCCG----------
ACCCCCCGGAGGGG---TTCGAGGG-
TTGAAATGACGCTCGGACAGGCATGCCCGCCAGAATACTGGCGGGCGCAATGTGCGTTC
AAAGATTCGATGATTCACTGAATTCTGCAATTCACATTACTTATCGCATTTCGCTGCGTT
CTTCATCGATGCCAGAACCAAGAGATCCGTTGTTGAAAGTTTTG-
ATTCATTTTCGAAACGCCTACGAGAGGCGCCGAGAAAGGCTCAGATTATAAAAAAACCC
GCGAGGGGGTATACAATAAGAGTTTTAGGTTGGTCCTCCGGCGGGCGCCTT------------
GG----------TCCGGGGCTGC-----GACGCACCCGGGGCAGAG-ATCCCGCCGAGGCAACAGT--
TTGGTAA-CGTTCACATT-GGGTTTGGGA--------------GTTGTA-
AACTCGGTAATGATCCCTCCGCTGGTTCACCAACGGA---

>Melanconis_stilbostoma_strain_E01051_AY577814.1

---TCCTACCTGATCCGAGGTCAATTTT-
TCAAAAATGGGGGGT-GTTTTATGGCTAGAAG-TCC---CACT---AGTCTTTACAAACGAGG-
TATAAATTAC--TACGCTCAA--AATTCTA-GCGAGCCCGC--CACTACATTTCAG--
GGCATACCTTTT-------GACGGGTAGAG--------CCCCAACACCA---------------AAGACTAGGC-
-TTTGAGGG-
TTGAAATGACGCTCGAACAGGCATGCCCGCTGGAATACCAGCGGGCGCAATGTGCGTTC
AAAGATTCGATGATTCACTGAATTCTGCAATTCACATTACTTATCGCATTTCGCTGCGTT
CTTCATCGATGCCAGAACCAAGAGATCCGTTGTTGAAAGTTTTG-ATTCATTTATAG-----
TTGTTTTA--------------CTCAGAGTACTA------TTTTAAAA---------ATAAGAGTTTA---
TAGGGCCACCGGCCAGCCTGCTC-------------------
CTTTCTCCGAAGAAAAAGGGAATCCCTTTAAAAGATTCAGCTAATGCCGAGGCAACAGT
--TTGGTAT-AATTCACAAAGGGTTTCTGGGTAGCACCCGTG-
AGGGCGTTTGTTCCAGCAATGATCCCTCCGCTGGTTCACCAACGGAGACCTTGTTACGAC
TTTTACTTCC---

>Melanconis stilbostroma isolate SL428H

--CGAGGTCA--TTT-TTCAAAATTGGGGGT-
GTTTTATGGCTAGAAG-TCC---CACT---AGTCTTTACAAACGAGG-TATAAATTAC--
TACGCTCAA--AATTCTA-GCGAGCCCGC--CACTACATTTCAG--GGCATACCTTTT-------
GACGGGTAGAG--------CCCCAACACCA---------------AAGACTAGGC--TTTGAGGG-
TTGAAATGACGCTCGAACAGGCATGCCCGCTGGAATACCAGCGGGCGCAATGTGCGTTC
AAAGATTCGATGATTCACTGAATTCTGCAATTCACATTACTTATCGCATTTCGCTGCGTT
CTTCATCGATGCCAGAACCAAGAGATCCGTTGTTGAAAGTTTTG-ATTCATTTATAG-----
TTGTTTTA--------------CTCAGAGTACTA------TTTTAAAA---------ATAAGAGTTTA---
TAGGGCCACCGGCCAGCCTGCTC------------------
CTTTCTCCGAAGAAAAAGGGAATCCCTTTAAAAGATTCAGCTAATGCCGAGGCAACAGT
--TTGGTAT-AATTCACAAAGGGTTTCTGGGTAGCACCCGTG-
AGGGCGTTTGTTCCAGCAATGATCCCTCCGCTGGTTCACCAACGGAGACCTTGTTACGAT
TTTTAC--

>gi|411532197|gb|KC109754.1|_Chaetomium_globosum_strain_CBS_155.52_18S_ribosomal_RNA_gene_partial_sequence_internal_transcribed_spacer_1_5.8S_ribosomal_RNA_gene_and_internal_transcribed_spacer_2_comple

---GGTTAAAAGGTG-
GTTTAACGGCCGGAAC-C-----CGCA---GCACGCCCAGAGCGAG-ATGTATGCTAC--
TACGCTCGG--TGTGACA-GCGAGCCCGC--CACTGCTTTTCAG--GGCCTGCGGC---------
AGCCGCAGGT---------CCCCAACACAA--------------GCCCG--GGG---CTTGATGG-
TTGAAATGACGCTCGAACAGGCATGCCCGCCAGAATACTGGCGGGCGCAATGTGCGTTC
AAAGATTCGATGATTCACTGAATTCTGCAATTCACATTACTTATCGCATTTCGCTGCGTT
CTTCATCGATGCCAGAACCAAGAGATCCGTTGTTGAAAGTTTTG-ACTTATTCAG--------
TACAGAAGA------------CTCAGA-GAGG-------CCATAAAT-------TATCAAGAGTTTG----
GTGACCTCCGGCGGGCGCCCGC-------GGTGGGG----------
CCCAGGGGCGCCCGGGGGGTAAACCCCGGCGCCG---CCCGCCGAAGCAACGGT--
TTAGGTAACGTTCACAAT-GGTTTAGGGA-------------
GTTTTGCAACTCTGTAATGATCCCTCCGCTGGTTCACCAACGGAGA-----------------------------
----

>Chaetomium globosum isolate LD13H

---GGTTAAAAGGTG-
GTTTAACGGCCGGAAC-C-----CGCA---GCACGCCCAGAGCGAG-ATGTATGCTAC--
TACGCTCGG--TGTGACA-GCGAGCCCGC--CACTGCTTTTCAG--GGCCTGCGGC---------
AGCCGCAGGT---------CCCCAACACAA--------------GCCCG--GGG---CTTGATGG-
TTGAAATGACGCTCGAACAGGCATGCCCGCCAGAATACTGGCGGGCGCAATGTGCGTTC
AAAGATTCGATGATTCACTGAATTCTGCAATTCACATTACTTATCGCATTTCGCTGCGTT
CTTCATCGATGCCAGAACCAAGAGATCCGTTGTTGAAAGTTTTG-ACTTATTCAG--------
TACAGAAGA------------CTCAGA-GAGG-------CCATAAAT-------TATCAAGAGTTTG----
GTGACCTCCGGCGGGCGCCCGC-------GGTGGGG----------
CCCAGGGGCGCCCGGGGGGTAAACCCCGGCGCCG---CCCGCCGAAGCAACGGT--
TTAGGTAACGTTCACAAT-GGTTTAGGGA-------------
GTTTTGCAACTCTGTAATGATCCCTCCGCTGGTTCACCAACGGA---------------------------------
----

>Phialophora_sp._DF33_EU314707.1_voir_leptosphaeria_marina

--GAGCTACCTGATCCGAGGTCAA-
ACTTAGAAAGTTGGG-GGTTGCTGGCCAGCA--TC----CACC---GGGTCCCTATAGCGAG---
GAGTATTAC--TACGCATTAG-AGCCCAG-CGGGCGCCGC--CACTACTTTTAAG---
GCCCGCCGTTTCC---------GGCG-AG--------GCCCAAGACCA----------------AGCTAG---
GCTTGAGTG-
TTGTACTGACGCTCGAACAGGCATGCCCTGCGGAATACCACAGGGCGCAATGTGCGTTC
AAAGATTCGATGATTCACTGAATTCTGCAATTCACATTACTTATCGCATTTCGCTGCGTT
CTTCATCGATGCCAGAACCAAGAGATCCGTTGTTGAAAGTTTTA-ACTATTATATAG------
TA-------------------CTCAGACGACATG------TATAAAC-----------AGAGTTTAGG-----
GTCCTCTGGCGAGCGCTCA------------------------CCAGCGTGAG-CCGG--TGGCCGAAG--
CGG-----CTCGCCAAAGCAACAAA--GGTATAATTAACATAGGGTTGGA-----
GAGTCACCCTTG-CGGGCGATGTTCTCTGTAATGATC--

>gi|317415485|emb|FM200735.1|_Fungal_endophyte_sp._AP701_partial_18S_rRNA_gene_ITS1_5.8S_rRNA_gene_ITS2_and_partial_28S_rRNA_gene_isolate_AP701

---CCCTACCTGATCCGAGGTCAA-
CCTGATAAAATATGG-GGGTTCTGGCAGGGA--AC----CGAC---AGGACTCTATAGCGAG---
GAGTATTAC--TACGC-TCAG-AGCCTA--CCGGCACCGC--CACTGGTTTTAGA---
GGCCGCGAGACC-----------GCG-AG--------CCCCAATACTA----------------AGCTAG---
GCTTAATTGGCTATAATGACGCTCGAACAGGCATGCCCTGCGGAATACCACAGGGCGCA
ATGTGCGTTCAAAGATTCGATGATTCACTGAATTCTGCAATTCACATTACTTATCGCATT
TCGCTGCGTTCTTCATCGATGCCAGAACCAAGAGATCCGTTGTTGAAAGTTTTA-
ACTATTATATAG------TA-------------------CTCAGACATCAC-------TAAAATT-----------
CAGAGTT-TG-----GTCCTCTGGCAAACGCATGT-----------------------
ACAGGCAAGAGCCCGCAGTGGATTAACCACAG-----CCTGCCAAAGCAACAAG--
AGTAGAT-AGACACGGGTTGGGTATACCGAAGTATCCTTT-TGGGTAATG--------------------
----

>Ascomycete isolate LD534H

---TGATCCGAGGTCA--
CCTGATAAAATATGG-GGGTTCTGGCAGGGA--AC----CGAC---AGGACTCTATAGCGAG---
GAGTATTAC--TACGC-TCAG-AGCCTA--CCGGCACCGC--CACTGGTTTTAGA---
GGCCGCGAGACC-----------GCG-AG--------CCCCAATACTA----------------AGCTAG---
GCTTAATTGGCTATAATGACGCTCGAACAGGCATGCCCTGCGGAATACCACAGGGCGCA
ATGTGCGTTCAAAGATTCGATGATTCACTGAATTCTGCAATTCACATTACTTATCGCATT
TCGCTGCGTTCTTCATCGATGCCAGAACCAAGAGATCCGTTGTTGAAAGTTTTA-
ACTATTATATAG------TA-------------------CTCAGACATCAC-------TATAATT-----------
CAGAGTT-TG-----GTCCTCTGGCAAACGCATGT-----------------------
ACAGGCAAGAGCCCGCAGTGGATTAACCACAG-----CCTGCCAAAGCAACAAG--
AGTAGAT-AGACACGGGTTGGGTATACCGAAGTATCCTTT-
TGGGTAATGATCCTTCCGCAGGTTCACCTACGGACGCAGAAGCCCCTATAACTTGTTACA

GGGCCCGACTATATCTTA---

>Exosporium_stylobatum_strain_CBS_JQ044428.1

--CTACCTGATCCGAGGTCAA--
GATATAAATGTTG-------CTTACTGGATG--CCATCCGC----CGCGAAGAGAGACGCAAT--
TC---TGC--TGCGCTCAAAGC-CGCTGAAGTGG-CTGC--
CAATCGTTTTGAGGCGAGTCCGCGC----CG-AAGCGGGACAG--------ACGCCCAACACCA---
--------------AGCTGTG---CTTGAGGG-
TGTAAATGACGCTCGAACAGGCATGCCCTAAGGAATACCAAAGGGCGCAATGTGCGTTC
AAAGATTCGATGATTCACTGAATTCTGCAATTCACACTACTTATCGCATTTTGCTGCGTT
CTTCATCGATGCCAGAACCAAGAGATCCATTGTTGAAAGTTTTG-ATTTTTT-GTTTGT----
TTTTCAGA-------CGAT--TACTGTATTTAC----AAAGCGTTTGAGGA-
GTCCCCAGTAGCAGGCA---AGCCTGCTGAGGAA--ACGAACGGTGCTCCAAAAA-ATAGG-
-----------------CAAGGACGCTATCTCGAC---
AGGACAAAGGCCCTAC-------------
GATAATGATCCTTCCGCAGGTTCACCTACGGAAACCTTGTTACGACTT------------------------
----

>Exosporium stylobatum isolate AN122R

--CTGATCCGAGGTCA---GATATAAATGTTG---
----CTTACTGGATG--CCATCCGC----CGCGAAGAGAGACGCAAT--TC---TGC--
TGCGCTCAAAGC-CGCTGAAGTGG-CTGC--CAATCGTTTTGAGGCGAGTCCGCGC----CG-
AAGCGGGACAG--------ACGCCCAACACCA-----------------AGCTGTG---CTTGAGGG-
TGTAAATGACGCTCGAACAGGCATGCCCTAAGGAATACCAAAGGGCGCAATGTGCGTTC
AAAGATTCGATGATTCACTGAATTCTGCAATTCACACTACTTATCGCATTTTGCTGCGTT
CTTCATCGATGCCAGAACCAAGAGATCCATTGTTGAAAGTTTTG-ATTTTTT-GTTTGT----
TTTTCAGA-------CGAT--TACTGTATTTAC----AAAGCGTTTGAGGG-
GTCCCCAGTAGCAGGCA---AGCCTGCTGAGGAA--ACGAACGGTGCTCCAAAAA-ATAGG-
-----------------CAAGGACGCTATCTCGAC---
AGGACAAAAGCCCTAC-------------
GATAATGATCCTTCCGCAGGTTCACCTACGGAAACCTTGTTACGACTTTTACTTCCA-------
----

>Dendryphion_penicillatum_isolate_48/3.6.1_DQ865101.1

--CCCTACCTGATCCGAGGTCAA-
CGTTGATTTGTAGG-------CTTCATGGACG-CCCCGCCTCA---G----GCAGAAGCGCAAT-
TTG---TGC--TGCGCTCCAAAACCAATA-GGCCGGCTGC--
CAATCGTTTTAAGGCGAGTCTTTTC---------A---GACAA--------ACGCCCAACACCA-----------
-----AGCAAAG---CTTGAGGG-
TACAAATGACGCTCGAACAGGCATGCCCTTTGGAATACCAAAGGGCGCAATGTGCGTTC
AAAGATTCGATGATTCACTGAATTCTGCAATTCACACTACTTATCGCATTTCGCTGCGTT
CTTCATCGATGCCAGAACCAAGAGATCCGTTGTTGAAAGTTGTA-ATAATTTTTTGTTA----
TACTGA---------CGCTGACTGCAATTACAA----AAGGTTTATTGGT-
TTGTCCTTTTGGTGGGCG--AGCCC-ACCAAGGAA--ACAAGAAGTACGCAAAAGA-
CATGGGTGAAATATTCAGACAAGCTGGGCAAACACACGCCGACCCGACTTGCTTCAACG

CCGTGAGGCGGAGGCAAGCGGCAAAGCAATGTGTCTGANGCCCCAGCCTGCAGTCATAT
TG-GTAATGATCCTTCCGCAGGTTCACCTACGGAAACCTTGTTACGACTTTT-ACTTC-------
----

>Brunneiperidium gracilentum isolate AN44R

-------------TGCAAT-TTG---TGC--TGCGCTTCAAAACCAGTA-TGCCGGCTGC--
CAATTGTTTTAAGGCGAGTCTCGCA---------A---GACAA-G------ACGCCCAACACCA---------
-------AGCAAGG---CTTGAGGG-
TACAAATGACGCTCGAACAGGCATGCCCTTTGGAATACCATAGGGCGCAATGTGCGTTC
AAAGATTCGATGATTCACTGAATTCTGCAATTCACACTACTTATCGCATTTCGCTGCGTT
CTTCATCGATGCCAGAACCAAGAGATCCGTTGTTGAAAGTTGTA-ATAATTTTGT-TTT----
TACTGA---------CGCTGACTGCAATTACAA----AAGGTTTATGGGG-
GTGTCCTGTTGGTGGGCG--AACCC-ACCCAGGAA--ACAAAAAGTACGCAAAAGA-
CATGGGTGAA-TATTCAAACAGGCTGGGAAAAAGCACGCCGGACCG-
CTGCCTACAACGC---

>gi|89276701|gb|DQ411539.1|_Dendryphiella_arenaria_strain_CBS_181.58_18S_ribosomal_RNA_
gene_partial_sequence_internal_transcribed_spacer_1_5.8S_ribosomal_RNA_gene_and_internal_tra
nscribed_spacer_2_come

--CCCTACCTGATCCGAGGTCAA-
AAGTTAAAAAAAAATTTATGTCTTGATGGATG-CTCAACCATG---
GCTGATCAGAAGTGCAAGATTG---TGC--TGCGCTCCGAAACCAGTA-GGCCGGCTGC--
CAATCATTTTAAGGCGAGTCTCGTG-------AGA---GACAAAG------ACGCCCAACACCA-------
----------AGCAAAG---CTTGAGGG-
TACAAATGACGCTCGAACAGGCATGCCCTTTGGAATACCAAAGGGCGCAATGTGCGTTC
AAAGATTCGATGATTCACTGAATTCTGCAATTCACACTACGTATCGCATTTCGCTGCGTT
CTTCATCGATGCCAGAACCAAGAGATCCGTTGTTGAAAGTTGTA-ATAATTACATTGTG---
-TACTGA---------CGCTGATTGCAATTACAAAAA-AAGGTTTATGGTT-
GGGTCCTGGTGGCGGGCG--AACCC-GCCCAGGAA--ACAAGAAGTGCGCAAAAGA-
CATGGGTGAATAATTCAGACAAGCTGGAG-----------------CCCCCACCGAGAT-----------------
-----------------GAGGTCCCAACCCGCTTTCATATTGTGTAATGATCCCTCCGCAGGTC---------
----

>Paradendryphiella arenaria isolate LD40H

---GAAAAATGTG-
GTCTTGATGGATG-CTCAACCATG---GCTGATCAGAAGTGCAAGATTG---TGC--
TGCGCTCCGAAACCAGTA-GGCCGGCTGC--CAATCATTTTAAGGCGAGTCTCGTG-------
AGA---GACAAAG------ACGCCCAACACCA----------------AGCAAAG---CTTGAGGG-
TACAAATGACGCTCGAACAGGCATGCCCTTTGGAATACCAAAGGGCGCAATGTGCGTTC
AAAGATTCGATGATTCACTGAATTCTGCAATTCACACTACGTATCGCATTTCGCTGCGTT
CTTCATCGATGCCAGAACCAAGAGATCCGTTGTTGAAAGTTGTA-ATAATTACATTGTT---

-TACTGA---------CGCTGATTGCAATTACAAAAA-AAGGTTTATGGTT-
GGGTCCTGGTGGCGGGCG--AACCC-GCCCAGGAA--ACAAGAAGTGCGCAAAAGA-
CATGGGTGAATAATTCAGACAAGCTGGAG-----------------CCCCCACCGAGAT-----------------
----
GAGGTCCCAACCCGCTTTCATATTGTGTAATGATCCCTCCGCAGGTTCACCTACGGA------
----

>gi|92430229|gb|DQ491491.1|_Botryotinia_fuckeliana_isolate_AFTOL-ID_59_internal_transcribed_spacer_1_partial_sequence_5.8S_ribosomal_RNA_gene_complete_sequence_and_internal_transcribed_spacer_2_partiae

---TCCCTACCTGATCCGAGGTCAA-
CCATAGAAAAATTTG-GGTT-TTGGCAGAAG--CA----CACC---GAGAACCTGTAACGAG---
AGATATTAC--TACGT-TCAG-GACCCAG-C-GGCGCCGC--CACTGATTTTAGA---
GCCTGCCATTACT---------GACATAG--------ACTCAATACCA-----------------AGCTAA---
GCTTGAGGG-
TTGAAATGACGCTCGAACAGGCATGCCCCCCGGAATACCAAGGGGCGCAATGTGCGTTC
AAAGATTCGATGATTCACTGAATTCTGCAATTCACATTACTTATCGCATTTCGCTGCGTT
CTTCATCGATGCCAGAACCAAGAGATCCGTTGTTGAAAGTTTTA-ACTATTATATAG------
TA-------------------CTCAGACGACATT------AATAAAA-----------AGAGTTTTGGT----
ATTCTCTGGCGAGCATACA---AGGCCCGAAGGCAG-----
CTCGCCAAAGCAACAAA--GTAATAATACACAAGGGTGGGA------GGTCTACCCTTT-
CGGGC-
ATGAACTCTGTAATGATCCTTCCGCAGGTTCACCTACGGAAACCTTGTTACAACTTTTTA
CTTCCAT---

>gi|557913966|gb|KF467107.1|:1-483_Fungal_sp._E13606A_internal_transcribed_spacer_1_partial_sequence_5.8S_ribosomal_RNA_gene_complete_sequence_and_internal_transcribed_spacer_2_partial_sequence

---AA-------
GCTTTTGGAGAGCCGAGCCGCC---GGGTTCCACAAGCGCGTC--TG---TGC--
TGCGCTCACGGCCGGCGATTGCGGGCTGC--CAATGACTTTGAGGTGAGTCGCCGCGCA-
GG-AGGCGGGACAA--------GCACCCAATACCA-----------------AGCTGGG---CTTGAGGG-
GTTAAATGACGCTCGAACAGGCATGCCCTACAGAATACTATAGGGCGCAATGTGCGTTC
AAAGATTCGATGATTCACTGAATTCTGCAATTCACACTACTTATCGCATTTCGCTGCGTT
CTTCATCGATGCCAGAACCAAGAGATCCATTGTTGAAAGTTTTG-ATTATTATGTTTGT----
TTTCAGAC-------AAATACTGCAAACTGCAA----AAGGTTTTTTGGGG-
GTCCTCGCTGGCAGGCG---TCCCTGCCGAGGAAACATGAA-GGTGCTCAAAATC-
AAGGGTGGTAAGAACGCG---TGTGGCCGCCCTAACCCCCCAGCAATTGGAGAAGG---------
------------------------GGGGATCACGGGGCGA--

>Arthopyrenia salicis isolate AN120R

---ACCTGATCCGAGGTCAA-
TTGTGGTTGAATAA-------GCTTTTGGAGAGCCGAGCCGCC---GGGGTCCACAAGCGCGTT-
-TG---TGC--TGCGCTCACGGCCGGCGATTGCGGGCTGC--
CAATGACTTTGAGGTGAGTCGCCGCGCG-AG-AGGCGGGACAA--------
GCACCCAATACCA----------------AGCTGGG---CTTGAGGG-
GTTAAATGACGCTCGAACAGGCATGCCCTACAGAATACTATAGGGCGCAATGTGCGTTC
AAAGATTCGATGATTCACTGAATTCTGCAATTCACACTACTTATCGCATTTCGCTGCGTT
CTTCATCGATGCCAGAACCAAGAGATCCATTGTTGAAAGTTTTG-ATTATTATGTTTGT----
TTTCAGAC-------AAATACTGCAAACTGCAA----AAGGTTTTTTGGGG-
GTCCTCGCTGGCAGGCG---TCCCTGCCGAGGAAACATGAA-GGTGCTCAAAATC-
AAGGGTGGTAAGAACGCG---TGTGGCCGCCCTAACCCCCCAGCAATTGGAGAAGG---------
------------------------GGGGATCACGGGGCGACCGCGTT-----
ATGGTAATGATCCTTCCGAAGGTTCACCTACGGAAACCTTGTTACGATTTTT-----------------
----

>Embellisia_sp._REF150_JN859370.1

---TCCTACCTGATCCGAGGTCAA-
AAGTTGAAAAAAGG-------CTTGTTGGACG-CT-GACCTTG---GCTGGAAAAGAGTGCGAC-
TTG---TGC--TGCGCTCCGAAACCAGTA-GGCCGGCTGC--
CAATGACTTTAAGGCGAGTCTCCAGCGGACT-GGA---GACAA-G------ACGCCCAACACCA-
---------------AGCAAAG---CTTGAGGG-
TACAAATGACGCTCGAACAGGCATGCCCTTTGGAATACCAAAGGGCGCAATGTGCGTTC
AAAGATTCGATGATTCACTGAATTCTGCAATTCACACTACGTATCGCATTTCGCTGCGTT
CTTCATCGATGCCAGAACCAAGAGATCCGTTGTTGAAAGTTGTA-ATTATTATGTTGT-----
TACTGA---------CGCTGATTGCTATTACAA----AAGGTTTATGAGT-TG-
TCCTTGTGGCGGGCG--AGCCCCACCAAGGAA--ACAAGAAGTACGCAAAAGA-
CACGGGTGAATAATTCAGCAAGGCTAGGC----------------TCTCCAGCAGCGCAC---------
GCCGTAAAGCAATGCACTACTACAGAGTATCCAGCCCGCTTTCATATTGTGTAATGATCC
CTCCGCAGGTTCACCTACGGAGACCTTGTTACGACTTTT-ACTTC-------------------------------
----

>Embellisia sp. isolate SL468T

---CGAGGTCAA-AAGTTGAAAAAAGG-----
--CTTGTTGGACG-CT-GACCTTG---GCTGGAAAAGAGTGCGAC-TTG---TGC--
TGCGCTCCGAAACCAGTA-GGCCGGCTGC--
CAATGACTTTAAGGCGAGTCTCCAGCGGACT-GGA---GACAA-G------ACGCCCAACACCA-
---------------AGCAAAG---CTTGAGGG-
TACAAATGACGCTCGAACAGGCATGCCCTTTGGAATACCAAAGGGCGCAATGTGCGTTC
AAAGATTCGATGATTCACTGAATTCTGCAATTCACACTACGTATCGCATTTCGCTGCGTT
CTTCATCGATGCCAGAACCAAGAGATCCGTTGTTGAAAGTTGTA-ATTATTATGTTGT-----
TACTGA---------CGCTGATTGCTATTACAA----AAGGTTTATGAGT-TG-
TCCTTGTGGCGGGCG--AGCCC-ACCAAGGAA--ACAAGAAGTACGCAAAAGA-
CACGGGTAAATAATTCAGCAAGGCTAGGC----------------TCTCCAGCAGCGCAC---------
GCCGTAAAGCAATGCACTACTACAGAGTATCCAGCCCGCTTTCATATTGTGTAATGATCC
CTCCGCAGGTTCACCTACGGAGACCTTGTTACGACTTTT-ACTTCCA---------------------------
----

>Paraphaeosphaeria_sporulosa_strain_CBS_317.81_JX496074.1

--CCCTACCTGATCCGAGGTCAA-
AGACGGTAATGTTG-------CTTCGTGGACG--CGGGCCACG---CCCCCCCGCAGACGCAAT--
TG---TGC--TGCGCGAGAGGA-GGCAAGGACCG-CTGC--
CAATGAATTTGGGGCGAGTCCGCGCGC--GA-AGGCGGGACAG--------
ACGCCCAACACCA-----------------AGCAGAG---CTTGAGGG-
TGTAGATGACGCTCGAACAGGCATGCCCCATGGAATACCAAGGGGCGCAATGTGCGTTC
AAAGATTCGATGATTCACTGAATTCTGCAATTCACACTACTTATCGCATTTCGCTGCGTT
CTTCATCGATGCCAGAGCCAAGAGATCCATTGTTGAAAGTTGTA-ACGATT--GTTTGT----
ATCAGAAC-------AGGTAATGCTAGATGCAA----AAAAGGTTTTGTTA-
AGTTCCAGCGGCAGGTT---GCCCCGCCGAAGGAGAACGAAAGGTGCTCGTAAAA-
AAAGGATGCAGGAATGCGGCGCGTGAGGGTGTTACCCCTACCACCCGGGAGAGAACCC
CC------------------------------GAGGGCCGCGACCGCACCTGGTT-
GAGATGGATAATGATCCTTCCGCAGGTTCACCTACGGAAACCTTGTTACGACTTTTACT
TCC--

>Parasphaeosphaeria sporulosa isolate LD53H

--GTAATGTTG-------
CTTCGTGGACG--CGGGCCACG---CCCCCCCGCAGACGCAAT--TG---TGC--
TGCGCGAGAGGA-GGCAAGGACCG-CTGC--CAATGAATTTGGGGCGAGTCCGCGCGC--
GA-AGGCGGGACAG--------ACGCCCAACACCA-----------------AGCAGAG---CTTGAGGG-
TGTAGATGACGCTCGAACAGGCATGCCCCATGGAATACCAAGGGGCGCAATGTGCGTTC
AAAGATTCGATGATTCACTGAATTCTGCAATTCACACTACTTATCGCATTTCGCTGCGTT
CTTCATCGATGCCAGAGCCAAGAGATCCATTGTTGAAAGTTGTA-ACGATT--GTTTGT----
ATCAGAAC-------AGGTAATGCTAGATGCAA----AAAAGGTTTTGTTA-
AGTTCCAGCGGCAGGTT---GCCCCGCCGAAGGAGAACGAAAGGTGCTCGTAAAA-
AAAGGATGCAGGAATGCGGCGCGTGAGGGTGTTACCCCTACCACCCGGGAGAGAACCC
CC------------------------------GAGGGCCGCGACCGCACCTGGTT-
GAGATGGATAATGATCCTTCCGCAGGTTCACCTACGGA---

>Paraphaeosphaeria_neglecta_strain_CBS_434.71B_JX496204.1

--CCCTACCTGATCCGAGGTCAA-
AGACGGTAATGTTG-------CTTCGTGGACG--CGGGCCACG---CCCCCCCGCAGACGCAAT--
TG---TGC--TGCGCGAGAGGA-GGCAAAGACCG-CTGC--
CAATGAATTTGGGGCGAGTCCGCGCGC--AG-AGGCGGGACAG--------
ACGCCCAACACCA-----------------AGCAGAG---CTTGAGGG-
TGTAGATGACGCTCGAACAGGCATGCCCCATGGAATACCAAGGGGCGCAATGTGCGTTC
AAAGATTCGATGATTCACTGAATTCTGCAATTCACACTACTTATCGCATTTCGCTGCGTT
CTTCATCGATGCCAGAGCCAAGAGATCCATTGTTGAAAGTTGTA-ACGATT--ATTTGT----
ATCAGAAC-------AGGTAATGCTAGATGCAA----
AAAAGGTTTTGTTTTGGTTCCAGCGGCAGGTT---
GCCCCGCCGAAGGAGAACGAAAGGTGCTCGTAAAA-
AGAGGGTACAGGCAGGCGGCGCGTGAGGGTGTTACCCCTACCACCCGGGAGCAAGCCC
CC------------------------------

GGGGGCCGCGACCGCACCTGGTTTGAGATGGATAATGATCCTTCCGCAGGTTCACCTAC
GGAAACCTTGTTACG---

>gi|663232088|gb|KJ869114.1|_Phaeosphaeria_poagena_strain_CBS_136771_18S_ribosomal_RNA_gene_partial_sequence_internal_transcribed_spacer_1_5.8S_ribosomal_RNA_gene_and_internal_transcribed_spacer_2_c(2)

---CCCTACCTGATCCGAGGTCAA-
AAGTGAAAAAGAGG-------CTTTGTGGATG-CC--ACTGTT---TAGAGACTAAGACGCAAA-
ATG---TGC--TGCGCTT-ACTACCAAAAACACTGGCTGC--
CAATTACTTTAAGGCGAGTCTAATCACTAAA-GAAAAAGACAAG-------
ACGCCCAACACCA-----------------AGCAGAG---CTTGAGGG-
TACAAATGACGCTCGAACAGGCATGCCCCATGGAATACCAAGGGGCGCAATGTGCGTTC
AAAGATTCGATGATTCACTGAATTCTGCAATTCACACTACTTATCGCATTTCGCTGCGTT
CTTCATCGATGCCAGAACCAAGAGATCCGTTGTTGAAAGTTGTA-ATTATTATAAATAA---
-TTCAGA---------CGCTGATTGAAGATTTAA----AAAGGTTATA-
GTTTTGTCCAACCGGCGAGCA--AGCCC-GCCGAGGAA--ACAATAGGTACGCAAAAAA-
CAAGGGTATAAA---CAAGTAGCT---
---------AGGCTA-CTGAATGTAATGATCCTTCCGCAGGTTCACCTACGGAAACCTTGTT------
----

>Phaeosphaeria poagena isolate SL470T

---CTGATCCGAGGTCAA-A-
GTGAAAAAGAGG-------CTTTGTGGATG-CC--ACTGTT---TAGAGACTAAGACGCAAA-
ATG---TGC--TGCGCTT-ACTACCAAAAACACTGGCTGC--
CAATTACTTTAAGGCGAGTCTAATCACTAAA-GAAAAAGACAAG-------
ACGCCCAACACCA-----------------AGCAGAG---CTTGAGGG-
TACAAATGACGCTCGAACAGGCATGCCCCATGGAATACCAAGGGGCGCAATGTGCGTTC
AAAGATTCGATGATTCACTGAATTCTGCAATTCACACTACTTATCGCATTTCGCTGCGTT
CTTCATCGATGCCAGAACCAAGAGATCCGTTGTTGAAAGTTGTA-ATTATTATAAATAA---
-TTCAGA---------CGCTGATTGAAGATTTAA----AAAGGTTATA-
GTTTTGTCCAACCGGCGAGCA--AGCCC-GCCGAGGAA--ACAATAGGTACGCAAAAAA-
CAAGGGTATAAA---CAAGTAGCT---
---------AGGCTA-
CTGAATGTAATGATCCTTCCGCAGGTTCACCTACGGAAACCTTGTTACGATTTTTACTTC
CA--

>Phaeosphaeria sp. isolate AN596H

--CCTGATCCGAGGTCAA-A-
GTGAGAAAGAGG-------CTTTGTGGATG-CC--ACTGTT---TCCAGACTAAGACGCAAA-ATG-
--TGC--TGCGCTT-ACTACCAAAAACACTGGCTGC--
CAATTACTTTAAGGCGAGTCTAATCACTAAA-GAAAAAGACAAG-------
ACGCCCAACACCA-----------------AGCAAAG---CTTGAGGG-

TACAAATGACGCTCGAACAGGCATGCCCCATGGAATACCAAGGGGCGCAATGTGCGTTC
AAAGATTCGATGATTCACTGAATTCTGCAATTCACACTACTTATCGCATTTCGCTGCGTT
CTTCATCGATGCCAGAACCAAGAGATCCGTTGTTGAAAGTTGTA-ATTATTATGTATAA---
-TTCAGA---------CGCTGATTGAAGATTTAA----AAAGGTTATA-
GTTTTGTCCAATCGGCGAGCA--AGCCC-GCCGAGGAA--ACAATAGGTACGCAAAAAA-
CAAGGGTATAAA---CAAGTAGCT---
---------AGGCTA-
CTGAATGTAATGATCCTTCCGCAGGTTCACCTACGGAAACCTTGTTACGACTTTTTACTT
CC--

>gi|530746624|gb|KF251193.1|_Phaeosphaeria_vagans_strain_CBS_604.86_18S_ribosomal_RNA_gene_partial_sequence_internal_transcribed_spacer_1_5.8S_ribosomal_RNA_gene_and_internal_transcribed_spacer_2_compe

--CCCTACCTGATCCGAGGTCAA-AAGTTAAAAA-
AGG-------CT-TATGGACG-CA--AGTATT---ATCGGCTAGAATCGCAAA-ATG---TGC--
TGCGCTTCAATACCAAAA-CACTGGCTGC--
CAATTGCTTTAAGGCGAGTCCAAACGCAAAG-GAGAG-GACAA--------
ACACCCAACACCA-----------------AGCAGAG---CTTGAGGG-
TACAAATGACGCTCGAACAGGCATGCCCCATGGAATACCAAGGGGCGCAATGTGCGTTC
AAAGATTCGATGATTCACTGAATTCTGCAATTCACACTACTTATCGCATTTCGCTGCGTT
CTTCATCGATGCCAGAACCAAGAGATCCGTTGTTGAAAGTTGTA-ATTATTAAGTTTT-----
TTCAGA---------CGCTGATTGAAAATT-AA----AAAGGTTATA-
GTTTTGTCCAATCGGCGGGCA--AGCCC-GCCGAGGAA--ACA-TGAGTGCGCAAAAGA-
CAAGGGTACAGA---
CAGAGGGCCTGCCGCTCATCAGTAAATTAATACTATGCGGAGTTACA--------------------------
--GCGCCTCCCGACCAGTAGCAAGCTA-
CTGAATGTAATGATCCTTCCGCAGGTTCACCTACGGAA---

>Phaeosphaeria vagans isolate SL539T

---CCTGATCCGAGGTCAA-AAGTTAAAAA-
AGG-------CT-TATGGACG-CA--AGTATT---ATCGGTTAGAATCGCAAA-ATG---TGC--
TGCGCTTCAATACCAAAA-CACTGGCTGC--
CAATTGCTTTAAGGCGAGTCCAAACGCAAAG-GAGAG-GACAA--------
ACACCCAACACCA-----------------AGCAGAG---CTTGAGGG-
TACAAATGACGCTCGAACAGGCATGCCCCATGGAATACCAAGGGGCGCAATGTGCGTTC
AAAGATTCGATGATTCACTGAATTCTGCAATTCACACTACTTATCGCATTTCGCTGCGTT
CTTCATCGATGCCAGAACCAAGAGATCCGTTGTTGAAAGTTGTA-ATTATTAAGTTTT-----
TTCAGA---------CGCTGATTGAAAATT-AA----AAAGGTTATA-
GTTTTGTCCAATCGGCGGGCA--AGCCC-GCCGAGGAA--ACA-TGAGTGCGCAAAAGA-
CAAGGGTACAGA---
CAGAGGGCCTGCCGCTCATCAGTAAATTAATACTATGCGGAGTTACA--------------------------
--GCGCCTCCCGACCAGTAGCAAGCTA-
CTGAATGTAATGATCCTTCCGCAGGTTCACCTACGGAAACCTTGTTACGATTTTTT---------
----

>Cf._Stagonospora_sp._CPC_22155_KF251268.1

---CCCTACCTGATCCGAGGTCAA-
AAGTGAAAAAGTAG-------CTATATGGATG-CC--GCCATT---TAAGGACTAAGACGCAAA-
ATG---TGC--TGCGCTT-ACTACCAAAAACACTGGCTGC--
CAATGACTTTAAGGCGAGTCTAATCACAAGA-GAAAA-GACAAA-------
ACGCCCAACACCA-----------------AGCAAAG---CTTGAAGG-
TACAAATGACGCTCGAACAGGCATGCCCCATGGAATACCAAGGGGCGCAATGTGCGTTC
AAAGATTCGATGATTCACTGAATTCTGCAATTCACACTACTTATCGCATTTCGCTGCGTT
CTTCATCGATGCCAGAACCAAGAGATCCGTTGTTGAAAGTTGTA-ATTATTATGTTTAA---
-TTCAGA---------CGCTGATTGAAGATTTAA----
AAAGGTTATAGGTTTTGTCCAACCGGCAGGCA--AGCCC-ACCGAGGAA--
ACAATAGGTACGCAAAAAAACAAGGGTGTAAA---CAAGTAGCT----------------------------------
--AGGCTA-
CTGAATGTAATGATCCTTCCGCAGGTTCACCTACGGAA---

>Phaeosphaeria sp. isolate SL472T

--GAGGTCAA-A-GTGAAAAAGTAG-------
CTATATGGATG-CC--GCTATT---TAAGGACTAAGACGCAAA-ATG---TGC--TGCGCTT-
ACTACCAAAAACACTGGCTGC--CAATGACTTTAAGGCGAGTCTAATCAGAAGA-GAAAA-
GACAAA-------ACGCCCAACACCA-----------------AGCAAAG---CTTGAAGG-
TACAAATGACGCTCGAACAGGCATGCCCCATGGAATACCAAGGGGCGCAATGTGCGTTC
AAAGATTCGATGATTCACTGAATTCTGCAATTCACACTACTTATCGCATTTCGCTGCGTT
CTTCATCGATGCCAGAACCAAGAGATCCGTTGTTGAAAGTTGTA-ATTATTATGTATAA---
-TTCAGA---------CGCTGATTGAAGATTTAA----
AAAGGTTATAAGTTTTGTCCAACCGGCAGGCA--AGCCC-ACCGAGGAA--
ACAATAGGTACGCAAAAAA-CAAGGGTGTAAA---CAAGTAGCT-----------------------------------
---AGGCTA-
CTGAATGTAATGATCCTTCCGCAGGTTCACCTACGGAAACCTTGTTACGACTTTTTACTT
C---
--

>Pilidium_concavum_voucher_BPI_1107274_AY487097.1

--ATATGCTT-
AAGTTCAGCGGGTATCCCTACCTGATCCGAGGTCAAA--CAAG---
AAGTTTTCAGTGTTTACCTATC---CACC-TTCGCAGAGATTACTATG--CGTGATTGAT-
AGTTACCACCAAG--GGTTTTAGACAAGGCG----TAAGCCACTG----------TCAATACTC-------
------------CGAAG------AATAG--
TGTAATGACGCTCGAACAGGCATGCCCTTCGGAATACCAAAGGGCGCAATGTGCGTTCA
AAGATTCGATGATTCACGGTGT-
CTGCAATTCACATTACTTATCGCATTTCGCTGCGTTCTTCATCGATGCCAGAACCAAGAG
ATCCGTTGTTGAAAGTTTTATAGTATTATAAAAGT------------------------
ACTCAGACGACAC---------TGATT-----------CAGAAT-------
AATCCTGCCCGAAGGCAAGGCA---
ACCGGTCAAACAACAAG-----------------AAAGGGAGATTGTGT--------------

TTCCACAACTCGGTAATGATCCCTCCGCAGGTTCACCTACGGAGACCTTGTTACGAC------
----

>Pilidium concavum isolate LD482H

CTGATCCGAGGTCAA---CAAG---AAGTTTTCAGTGTTTACCTATC---CACC-
TTCGCAGAGATTACTATG--CGTGATTGAT-AGTTACCACCAAG--
GGTTTTAGACAAGGCG----TAAGCCACTG----------TCAATACTC-------------------CGAAG------
AATAG--
TGTAATGACGCTCGAACAGGCATGCCCTTCGGAATACCAAAGGGCGCAATGTGCGTTCA
AAGATTCGATGATTCACGGTGT-
CTGCAATTCACATTACTTATCGCATTTCGCTGCGTTCTTCATCGATGCCAGAACCAAGAG
ATCCGTTGTTGAAAGTTTTATAGTATTATAAAAGT-----------------------
ACTCAGACGACAC---------TGATT-----------CAGAAT-------
AATCCTGCCCGAAGGCAAGGCA--
ACCGGTCAAACAACAAG-----------------AAAGGGAGATTGTGT--------------
TTCCACAACTCGGTAATGATCCCTCCGCAGGTTCACCTACGGAGACCTTGTTACGATTTT
TTA---

>gi|95107043|gb|DQ494689.1|_Psathyrella_candolleana_isolate_AFTOL-ID_1507_internal_transcribed_spacer_1_5.8S_ribosomal_RNA_gene_and_internal_transcribed_spacer_2_complete_sequence

--ATTGG-
TCAAGTAAAKTGTCCTTGCGGACGGTTAGAAGCAAGCATGAG----
TCCAATCCACGGCGTAGATAATTATCACACCAATAGACGGAAGCTCARTATGAGCTCG--
CTAATGCATTTCAGGGGAGCAGACCAGCACTGAGGCAGCCTGCAAAACCCCCACATCCA
AGCCTTCACCTGTCTCGTTACAAAACTGGTGAGGTTGAGAA--
TTTAATGACACTCAAACAGGCATGCTCCTCGGAATACCAAGGAGCGCAAGGTGCGTTCA
AAGATTCGATGATTCACTGAATTCTGCAATTCACATTACTTATCGCATTTCGCTGCGTTCT
TCATCGATGCGAGAGCCAAGAGATCCGTTGCTGAAAGTTGTATAGTTTTTATAGGC------
-------------------ATGAAAGCCCAT------
TGACTACATTCTAAATCATTCGAATGGGGTGTGTAAAAGACATAGAAC--
CTGGAAATTCAAAGAGAG---------
CCGGCCTTGTCGACGCAGCAATCCTTGCATCCGCTTTGT-TGCCAAAGCGAGGGG----
TATCCAGGCCTACAGATGRTTCACAGGTGGAAAGATAT----
GAATGACGGGCGTGCACAATGCTCCTAGGAGC-CAGCTACAACCAACGCCATA-
GATATTCGT---

>Psathyrella candolleana isolate SL563F

---ACCTGATTTGAGGTCAAATTGG-
TCAAGTAAATTGTCCTTGCGGACGGTTAGAAGCAAGCATGAG----
TCCAATCCACGGCGTAGATAATTATCACACCAATAGACGGAAGCTCAGTATGAGCTCG--
CTAATGCATTTCAGGGGAGCAGACCAGCACTGAGGCAGCCTGCAAAACCCCCACATCCA

AGCCTTCACCTGTCTCGTTACAAAACTGGTGAGGTTGAGAA--
TTTAATGACACTCAAACAGGCATGCTCCTCGGAATACCAAGGAGCGCAAGGTGCGTTCA
AAGATTCGATGATTCACTGAATTCTGCAATTCACATTACTTATCGCATTTCGCTGCGTTCT
TCATCGATGCGAGAGCCAAGAGATCCGTTGCTGAAAGTTGTATAGTTTTTATAGGC------
-------------------ATGAAAGCCCAT------
TGACTACATTCTAAATCATTCGAATGGGGTGTGTAAAAGACATAGAAC--
CTGGAAATTCAAAGAGAG---------
CCGGCCTTGTCGACGCAGCAATCCTTGCATCCGCTTTGT-TGCCAAAGCGAGGGG----
TATCCAGGCCTACAAATGGTTCACAGGTGGAAAGATAT----
GAATGACGGGCGTGCACAATGCTCCTAGGAGC-CAGCTACAACCAACGCCATA-
GATATTCGTTAATGATCCTTCCGCAGGTTCACCTACGGAAACCTTGTTACGATTTTT-------
----

>Trametes_versicolor_voucher_HHB12282sp_JN164974.1_

---CCTACCTGATTTGAGGTCAGAT--G-TTAAA-
AAGTTGTCCTAATGGACGGTTAGAAGCTCGCCAAAACACTTCACGGTCACAGCGTAGAC
AATTATCACACTGAGAGCCGA----
TCCGTACGGAATCGAGCTAATGCATTCAAGAGGAGCCGACCAACAA-GGGCCAGC-----
AAGCCTCCAAGTCCAAGCTTATAG-------ATCACAAGGATTTATAAGTTGAGAA--
TTCCATGACACTCAAACAGGCATGCTCCTCGGAATACCAAGGAGCGCAAGGTGCGTTCA
AAGATTCGATGATTCACTGAATTCTGCAATTCACATTACTTATCGCATTTCGCTGCGTTCT
TCATCGATGCGAGAGCCAAGAGATCCGTTGCTAAAAGTTGTA-------TTATAGAT------------
-------------GCGTTAGACG--------CGTTTACATTCTGATACTTTAAAGTG---
TTTGTAGTATACATAGGCCGGCAGAATGCTCCCGTTAAG---------
GAGCCCACGCCAACCTA-CAGTAAGTGCACAGGGGTAGAGTGGATGAGCAGAGCG----
TGCACATGCCT-CGGAAGGC---CAGCTACAACTCGTT----TCAAAACT--
CGTTAATGATCCTTCCGCAGGTTCACCTACGGAAACCTTGTTACGACTTTTACTTCC-------
----

>Trametes versicolor isolate AN131R

--ACCTGATTTGAGGTCAGAT--G-TTAAA-
AATTTGTCCTAATGGACGGTTAGAAGCTCGCCAAAACACTTCACGGTCACAGCGTAGAC
AATTATCACACTGAGAGCCGA----
TCCGTACGGAATCGAGCTAATGCATTCAAGAGGAGCCGACCAACGA-GGGCCAGC-----
AAGCCTCCAAGTCCAAGCTTATAG-------ATCACAAGGATTTATAAGTTGAGAA--
TTCCATGACACTCAAACAGGCATGCTCCTCGGAATACCAAGGAGCGCAAGGTGCGTTCA
AAGATTCGATGATTCACTGAATTCTGCAATTCACATTACTTATCGCATTTCGCTGCGTTCT
TCATCGATGCGAGAGCCAAGAGATCCGTTGCTAAAAGTTGTA-------TTATAGAT------------
-------------GCGTTAGACG--------CGTTTACATTCTGATACTTTAAAGTG---
TTTGTAGTATACATAGGCCGGCAGAATGCTCCCGTTAAG---------
GAGCCCACGCCAACCTA-CAGTAAGTGCACAGGGGTAGAGTGGATGAGCAGAGCG----
TGCACATGCCT-CGGAAGGC---CAGCTACAACTCGTT----TCAAAACT--
CGTTAATGATCCTTCCGCAGGTTCACCTACGGAAACCTTGTTACGCT--------------------------
----

>gi|358001551|gb|JN942878.1|_Mucor_hiemalis_strain_DAOM_225705_internal_transcribed_space r_1_partial_sequence_5.8S_ribosomal_RNA_gene_complete_sequence_and_internal_transcribed_sp acer_2_partial_sequene

----
TCCCGCCTGATTTCAGATCAAATTTAAGAAAGTTTTATTTGGGAGGCCCCAGAGATAGTC
TTAATACTAGAGCATTCCTTTATATTAAAAAAATGTT-
CAGGCAGAAAGAACAATAGTTCAGGCC----TAATAGGTTTAAA--GAATTCAAACA----------
-AGTCGAAA-------TTCTCAATTCCATTC-------ACAATAAAATTATGA---
ATGTGGGGTGTTTTTGATACTGAAACAGGCGTGCTCAATGGAATACCATTGAGCGCAAG
TTGCGTTCAAAGACTCGATGATTCACTGAATA-
TGCAATTCACACTAGTTATCGCACTTTGCTACGTTCTTCATCGATGCGAGAACCAAGAGA
TCCGTTGTTAAAAGTTGTT---TTATAAGTTTTTTACGCTTATG---------------
TTACAATAATAA-------
TACTGAATTCTTTTGGTAAATAATTAATAGGATACCAGGTCTAAACCTGACTTCAGCTCG
GTTAACA-
TTCTAATAGTCTATCCCTATGACTAAAAGACATTCCTCAAACGCTGAAAATTAAAACAGT
TCA---
CAGTAAAAAAGAATGAACCTAACTAGAACAAGTTCTAGGAGGTCATCTAAATTATTTAA
TGATCC--

>Mucor hiemalis isolate LD450H

---TCAGATCAA-
TTTAAGAAAGTTTTATTTGGGAGGCCCCAGAGATAGTCTTAATACTAGAGCATTCCTTTA
TATTAAAAAAATGTT-CAGGCAGAAAGAACAATAGTTCAGGCC----TAATAGGTTTAAA--
GAATTCAAACA-----------AGTCGAAA-------TTCTCAATTCCATTC-------
ACAATAAAATTATGA---
ATGTGGGGTGTTTTTGATACTGAAACAGGCGTGCTCAATGGAATACCATTGAGCGCAAG
TTGCGTTCAAAGACTCGATGATTCACTGAATA-
TGCAATTCACACTAGTTATCGCACTTTGCTACGTTCTTCATCGATGCGAGAACCAAGAGA
TCCGTTGTTAAAAGTTGTT---TTATAAGTTTTTTACGCTTATG---------------
TTACAATAATAA-------
TACTGAATTCTTTTGGTAAATAATTAATAGGATACCAGGTCTAAACCTGACTTCAGCTCG
GTTAACA-
TTCTAATAGTCTATCCCTATGACTAAAAGACATTCCTCAAACGCTGAAAATTAAAACAGT
TCA---
CAGTAAAAAAGAATGAACCTAACTAGAACAAGTTCTAGGAGGTCATCTAAATTATTTAA
TGATCCTTCCGCAGGTTCACCTACGGAAACCTTGTTACGATTTTT---------------------------------
----
